# Maternal B-vitamin and vitamin D status before, during, and after pregnancy and the influence of supplementation preconception and during pregnancy: Prespecified secondary analysis of the NiPPeR double-blind randomized controlled trial

**DOI:** 10.1371/journal.pmed.1004260

**Published:** 2023-12-05

**Authors:** Keith M. Godfrey, Philip Titcombe, Sarah El-Heis, Benjamin B. Albert, Elizabeth Huiwen Tham, Sheila J. Barton, Timothy Kenealy, Mary Foong-Fong Chong, Heidi Nield, Yap Seng Chong, Shiao-Yng Chan, Wayne S. Cutfield, NiPPeR Study Group

**Affiliations:** 1 MRC Lifecourse Epidemiology Centre, University of Southampton, University Hospital Southampton, Southampton, United Kingdom; 2 NIHR Southampton Biomedical Research Centre, University of Southampton and University Hospital Southampton, NHS Foundation Trust, Southampton, United Kingdom; 3 Liggins Institute, University of Auckland, Auckland, New Zealand; 4 Singapore Institute for Clinical Sciences, Agency for Science, Technology and Research, Singapore; 5 Saw Swee Hock School of Public Health, National University of Singapore and National University Health System, Singapore; 6 Department of Obstetrics and Gynaecology, Yong Loo Lin School of Medicine, National University of Singapore, National University Health System, Singapore; 7 Department of Obstetrics and Gynaecology, National University Hospital, Singapore; 8 A Better Start, New Zealand National Science Challenge, Auckland, New Zealand; Cambridge University, UNITED KINGDOM

## Abstract

**Background:**

Maternal vitamin status preconception and during pregnancy has important consequences for pregnancy outcome and offspring development. Changes in vitamin status from preconception through early and late pregnancy and postpartum have been inferred from cross-sectional data, but longitudinal data on vitamin status from preconception throughout pregnancy and postdelivery are sparse. As such, the influence of vitamin supplementation on vitamin status during pregnancy remains uncertain. This study presents one prespecified outcome from the randomized controlled NiPPeR trial, aiming to identify longitudinal patterns of maternal vitamin status from preconception, through early and late pregnancy, to 6 months postdelivery, and determine the influence of vitamin supplementation.

**Methods and findings:**

In the NiPPeR trial, 1,729 women (from the United Kingdom, Singapore, and New Zealand) aged 18 to 38 years and planning conception were randomized to receive a standard vitamin supplement (control; *n* = 859) or an enhanced vitamin supplement (intervention; *n* = 870) starting in preconception and continued throughout pregnancy, with blinding of participants and research staff. Supplement components common to both treatment groups included folic acid, β-carotene, iron, calcium, and iodine; components additionally included in the intervention group were riboflavin, vitamins B6, B12, and D (in amounts available in over-the-counter supplements), myo-inositol, probiotics, and zinc. The primary outcome of the study was glucose tolerance at 28 weeks’ gestation, measured by oral glucose tolerance test. The secondary outcome reported in this study was the reduction in maternal micronutrient insufficiency in riboflavin, vitamin B6, vitamin B12, and vitamin D, before and during pregnancy. We measured maternal plasma concentrations of B-vitamins, vitamin D, and markers of insufficiency/deficiency (homocysteine, hydroxykynurenine-ratio, methylmalonic acid) at recruitment, 1 month after commencing intervention preconception, in early pregnancy (7 to 11 weeks’ gestation) and late pregnancy (around 28 weeks’ gestation), and postdelivery (6 months after supplement discontinuation). We derived standard deviation scores (SDS) to characterize longitudinal changes among participants in the control group and measured differences between the 2 groups. At recruitment, the proportion of patients with marginal or low plasma status was 29.2% for folate (<13.6 nmol/L), 7.5% and 82.0% for riboflavin (<5 nmol/L and ≤26.5 nmol/L, respectively), 9.1% for vitamin B12 (<221 pmol/L), and 48.7% for vitamin D (<50 nmol/L); these proportions were balanced between the groups. Over 90% of all participants had low or marginal status for one or more of these vitamins at recruitment. Among participants in the control group, plasma concentrations of riboflavin declined through early and late pregnancy, whereas concentrations of 25-hydroxyvitamin D were unchanged in early pregnancy, and concentrations of vitamin B6 and B12 declined throughout pregnancy, becoming >1 SDS lower than baseline by 28 weeks gestation. In the control group, 54.2% of participants developed low late-pregnancy vitamin B6 concentrations (pyridoxal 5-phosphate <20 nmol/L). After 1 month of supplementation, plasma concentrations of supplement components were substantially higher among participants in the intervention group than those in the control group: riboflavin by 0.77 SDS (95% CI 0.68 to 0.87, *p* < 0.0001), vitamin B6 by 1.07 SDS (0.99 to 1.14, *p* < 0.0001), vitamin B12 by 0.55 SDS (0.46 to 0.64, *p* < 0.0001), and vitamin D by 0.51 SDS (0.43 to 0.60, *p* < 0.0001), with higher levels in the intervention group maintained during pregnancy. Markers of vitamin insufficiency/deficiency were reduced in the intervention group, and the proportion of participants with vitamin D insufficiency (<50 nmol/L) during late pregnancy was lower in the intervention group (35.1% versus 8.5%; *p* < 0.0001). Plasma vitamin B12 remained higher in the intervention group than in the control group 6 months postdelivery (by 0.30 SDS (0.14, 0.46), *p* = 0.0003). The main limitation is that generalizability to the global population is limited by the high-resource settings and the lack of African and Amerindian women in particular.

**Conclusions:**

Over 90% of the trial participants had marginal or low concentrations of one or more of folate, riboflavin, vitamin B12, or vitamin D during preconception, and many developed markers of vitamin B6 deficiency in late pregnancy. Preconception/pregnancy supplementation in amounts available in over-the-counter supplements substantially reduces the prevalence of vitamin deficiency and depletion markers before and during pregnancy, with higher maternal plasma vitamin B12 maintained during the recommended lactational period.

**Trial registration:**

ClinicalTrials.gov NCT02509988; U1111-1171-8056.

## Abstract 2

In this pre-specified secondary analysis of the NiPPeR double-blind randomized controlled trial, Keith M Godfrey and colleagues investigate maternal B-vitamin and vitamin D status before, during and after pregnancy, and the influence of supplementation preconception and during pregnancy

## Author summary

**Why was this study done?:**

- A mother’s vitamin status preconception and during pregnancy are important for healthy pregnancy outcomes and offspring development.
- Truly longitudinal data on changes in vitamin status from preconception to early and late pregnancy and postpartum are sparse, with changes generally inferred from cross-sectional data and lower pregnancy concentrations often ascribed to plasma volume expansion.
- Few studies have evaluated the influence of vitamin supplementation preconception and during pregnancy.

**What did the researchers do and find?:**

- As prespecified secondary outcomes of a randomized trial, this study characterized longitudinal patterns of maternal vitamin status from preconception, through early and late pregnancy, to 6 months postdelivery, and determined the influence of supplementation.
- Significant proportions of preconception women living in high-income countries had marginal or low status of folate, riboflavin, vitamin B12, and vitamin D, and many developed markers of vitamin B6 deficiency in late pregnancy.
- In the absence of intervention supplementation, maternal plasma concentrations of different vitamins showed differing longitudinal patterns from preconception to early and late pregnancy, and markers of functional B6 and B12 insufficiency increased during pregnancy.
- Our trial indicates that preconception and pregnancy supplementation in amounts available in over-the-counter supplements substantially reduced the prevalence of deficiency/depletion markers before and during pregnancy, with a higher maternal plasma vitamin B12 maintained during the recommended lactational period.

**What do these findings mean?:**

- The study findings are inconsistent with plasma volume expansion wholly accounting for lower gestational vitamin concentrations.
- In the setting of advocacy for more sustainable diets that are likely to be less nutrient dense, the findings suggest a need to reappraise dietary recommendations for preconception and pregnancy and to consider further the role of multiple micronutrient supplements in women living in higher-income countries.

## Introduction

There is an increasing consensus that multiple micronutrient supplementation of pregnant women living in low-middle-income countries is beneficial for pregnancy outcomes [1]. In high-income countries, there are few large-scale trials of gestational micronutrient supplementation, resulting in less consensus about the need for individual or multiple micronutrient supplements to be taken. The principal exception relates to preconception and early pregnancy folic acid supplementation and fortification programs, underpinned by the landmark Medical Research Council trial [2]. Vitamin D supplementation is also generally recommended, in part based on the recent Maternal Vitamin D Osteoporosis Study (MAVIDOS) trial [3]; MAVIDOS used a higher dose of vitamin D than is recommended in many settings (25 μg of cholecalciferol daily from early pregnancy until delivery versus 10 μg daily recommended in countries such as the United Kingdom [4]), which reduced the incidence of infantile atopic eczema in the offspring [5] and improved measures of bone health in the children at age 4 years [6].

Recent evidence from animal studies demonstrates that maternal nutritional status prior to conception can have lasting effects on the offspring. This highlights a critical knowledge gap with regard to the importance of maternal micronutrient status before and during human pregnancy [7]. Evidence from human studies supporting a role for preconception micronutrient status is largely observational but does point to important implications for pregnancy outcomes and long-term offspring health [8]. Examples include the associations of suboptimal vitamin B12 and B6 status with an increased risk of preterm birth [9] and of maternal preconception iodine deficiency with lower child IQ [10].

To date, maternal micronutrient status preconception has largely been inferred from pregnancy data, and truly longitudinal studies describing changes from preconception to early and late pregnancy and postpartum have not previously been conducted. The ongoing significant prevalence of micronutrient insufficiencies among adolescent girls and women of reproductive age in high-income countries highlights the importance of documenting such changes [11]. Moreover, lower pregnancy concentrations are often ascribed to plasma volume expansion [12,13], without consideration of the longitudinal pattern or measurement of insufficiency/deficiency markers.

Human trials of micronutrient supplementation commencing before pregnancy remain relatively few in number; while they have not always shown benefits for maternal and offspring outcomes [14], a recent small trial did, however, report that maternal vitamin B12 supplementation from preconception until delivery improved offspring neurodevelopment at age 2 years [15]. Development of evidence-based guidelines for micronutrient intake before/during pregnancy is additionally hindered by the paucity of randomized trial data on the change in maternal micronutrient status resulting from preconception and pregnancy supplementation using amounts available in over-the-counter supplements.

The NiPPeR trial [16] is a multicenter, double-blind, randomized controlled trial of a nutritional supplement containing micronutrients, myo-inositol, and probiotics, whose primary outcome was the maintenance of euglycemia during pregnancy. The trial found no difference in gestational glycemia between study arms, but there was a significant reduction in preterm delivery, preterm prelabor rupture of membranes and major postpartum hemorrhage with the intervention compared with controls, who received a standard micronutrient supplement [17]. Longitudinal blood sampling in the NiPPeR trial provided an opportunity to determine the changes in maternal plasma concentrations of B-vitamins, vitamin D, and insufficiency/deficiency markers (homocysteine, hydroxykynurenine-ratio, methylmalonic acid) from preconception, through early and late pregnancy and to 6 months postpartum (notably in the control group), and to examine the influence of maternal supplementation preconception and during pregnancy on mean concentrations and markers of low vitamin status as prespecified secondary outcomes.

## Participants and methods

### Trial study design

The study protocol has been previously published [16]. Briefly, women planning a pregnancy were recruited from the community across 3 study sites in the UK, Singapore, and New Zealand, between 2015 and 2017. Trial exclusion criteria were pregnancy/lactation at recruitment, assisted conception (apart from taking clomiphene or letrozole alone), serious food allergy, preexisting diabetes mellitus, use of hormonal contraception, or taking metformin, systemic steroids, anticonvulsants, or treatment for HIV, hepatitis B or C in the past month. With stratification by site and ethnicity to ensure balanced allocation, 1,729 women were randomly assigned by an electronic database to receive intervention (*n* = 870) or control (*n* = 859) nutritional supplements from preconception until delivery. Maternal blood samples were collected from 870 intervention and 857 control women at preconception (at recruitment and 1 month after commencing supplementation), and then in early and late pregnancy, and 6 months postdelivery in those who became pregnant. Singleton pregnancies fulfilling the study criteria and reaching 28 weeks’ gestation with data on plasma vitamins at baseline and in late pregnancy were achieved in 580 women (CONSORT diagram shown in S1 Fig), with 512 followed up at 6 months postdelivery. Analyses were based on modified intention-to-treat principles, whereby we excluded individuals who did not have outcome data (i.e., were lost to follow-up from the study).

Intervention and control supplements with similar sensory characteristics and packaged as a powder in sachets labeled with one of 4 nonspeaking codes were stored at 2 to 6°C until made up in 250 ml water and taken twice daily. Ingredients common to control and intervention formulations were folic acid 400 μg/day, iron 12 mg/day, calcium 150 mg/day, iodine 150 μg/day, and β-carotene 720 μg/day; the intervention additionally included riboflavin 1.8 mg/day, vitamin B6 2.6 mg/day, vitamin B12 5.2 μg/day, vitamin D 10 μg/day, zinc 10 mg/day, myo-inositol 4 g/day, and probiotics (*Lactobacillus rhamnosus* and *Bifidobacterium animalis* sp. *lactis*). Quantities were either UK-recommended daily allowances for pregnant women (vitamin D, zinc, folic acid, iodine), minimal amounts for micronutrients linked with potential detrimental effects at higher doses (iron, β-carotene, calcium), or amounts enhanced above those typical in over-the-counter products (vitamins B6, B12, riboflavin) or used in previous trials (myo-inositol, probiotics) [18,19]. Following randomization, supplements were consumed from preconception until delivery of the baby. Participants and all study personnel remained blinded to treatment allocation until all pregnancy, delivery, and neonatal data had been collected, and analysis of the primary outcome completed. At enrolment preconception, sociodemographic characteristics, menstrual, obstetric, and health histories, and lifestyle habits were collected via interviewer-administered questionnaires. Weight and height were measured to derive body mass index (BMI). Adherence to the trial formulation ascertained by sachet counting was similar in the control and intervention groups; overall, 80.6% had 80% to 100% adherence averaged from recruitment to delivery, 15.8% had 60% to 80% adherence, and only 3.1% adherence below 60%, with similar adherence in the control and intervention groups (80.8% versus 80.4%, 15.8% versus 15.9%, and 2.4% versus 3.7%, respectively). Women were recommended to refrain from taking other supplements unless advised by their healthcare practitioner (e.g., significant iron deficiency anemia).

The trial was approved by the UK, Singapore, and New Zealand research ethics services (Southampton: Health Research Authority NRES Committee South Central Research Ethics Committee, reference 15/SC/0142l; Singapore: the National Healthcare Group Domain Specific Review Board, reference 2015/00205; New Zealand: the Health and Disability Ethics Committee, reference 15/NTA/21). The relevant regulatory authorities confirmed that the formulation was not an investigational medicinal product. All participants gave written informed consent. Trial oversight and monitoring were provided by an independent data and safety monitoring committee. This trial was prospectively registered at ClinicalTrials.gov NCT02509988, UTN U1111-1171-8056.

### Vitamin, vitamer, and metabolite measurements and status markers

EDTA plasma was obtained by centrifugation of peripheral venous blood samples at 1,600*g* at 4°C for 10 minutes and stored at −80°C. Aliquots were available for measurement of plasma vitamins and related vitamers/metabolites at the following 5 time points: preconception at recruitment (*n* = 1,721) and 1 month after commencing intervention (*n* = 1,454), in early (7 to 11 weeks’ gestation, *n* = 634) and late pregnancy (around 28 weeks’ gestation, *n* = 580), and postdelivery, 6 months after discontinuation of supplementation (*n* = 512). Using a targeted method based on liquid chromatography–tandem mass spectrometry (Bevital, Bergen, Norway) [20], we measured plasma concentrations of vitamins present in the control and intervention groups, related vitamers and metabolites selected as those that reflect vitamin status: homocysteine (reflecting 1-carbon status and other physiological states, and an indicator of folate and B-vitamin deficiency), riboflavin, flavin mononucleotide (reflecting riboflavin status), pyridoxal 5-phosphate (vitamin B6), 3-hydroxykynurenine (HK), kynurenic acid (KA), anthranilic acid (AA), 3-xanthurenic acid (XA), hydroxyanthranilic acid (HAA), cystathionine, cysteine, methylmalonic acid, and 25-hydroxyvitamin D3. Plasma folate and cobalamin (vitamin B12) were measured by microbiological assay, using a microtiter plate format on a robotic workstation employing a chloramphenicol-resistant strain of *Lactobacillus casei* (folate) and a colistin sulfate-resistant strain of *Lactobacillus leichmannii* (cobalamin) (Bevital, Bergen, Norway). Detailed quality control data for all analytes has been described previously, documenting coefficients of variation <10% [21]. Values below the assay limit of detection were set to half the limit of detection value. Any hemolysis was visually graded from zero to 4+; anthranilic acid, 3-hydroxyanthranilic acid, and 3-hydroxykynurenine values were set to missing for samples with a hemolysis score ≥2 (approximately equivalent to Hb 250 mg/dL), and those for folate set to missing for samples with 4+ hemolysis (approximately equivalent to Hb 1,000 mg/dL). Each analyte was checked for outliers (both statistically and clinically), and implausible values were set to missing.

As functional markers of vitamin B6 status, we calculated the plasma 3′-hydroxykynurenine ratio (HK ratio [HK:KA+AA+XA+HAA], a higher ratio reflecting effects of vitamin B6 insufficiency on tryptophan catabolism), along with the cystathionine/cysteine ratio (a higher ratio reflecting effects of vitamin B6 insufficiency on transsulfuration pathway regulation) [22]. There is inconsistency in the literature regarding thresholds for vitamin deficiency or insufficiency markers based on plasma measurements: For this study, we used plasma folate 13.6 nmol/L to define “marginal folate status” [23]; homocysteine ≥15 μmol/L to define “high plasma homocysteine” [24]; riboflavin <5 nmol/L (the lower limit of the reference range for the laboratory) and ≤26.5 nmol/L (the change-point of plasma riboflavin with erythrocyte glutathione reductase activation coefficient [25]) to define low riboflavin and “marginal riboflavin status,” respectively; pyridoxal 5-phosphate <20 nmol/L to define low B6 status; cobalamin <148 and <221 pmol/L to define B12 “deficiency” and “depletion,” respectively [26]; and plasma 25-hydroxyvitamin D3 <50 and <75 nmol/L to define “deficiency” and “insufficiency,” respectively [27].

### Statistics

The sample size was based on the trial primary outcome of gestational glycemia, as described previously [17]. Using mean and standard deviation values for 25-hydroxyvitamin D3 as an example, with alpha 0.05, we had 80% power to detect 0.12 SD and 0.18 standard deviation scores (SDS) differences between control and intervention groups 1 month after supplementation commencement (*n* = 707 control/*n* = 747 intervention) and in early pregnancy (*n* = 305 control/*n* = 329 intervention), respectively. Following log_e_ (natural logarithm) transformation where necessary, standardization was applied to all values of each analyte (i.e., approximately 4,902 samples) to derive SDS before being split into separate variables by time point/visit; the advantage of this approach is that for each analyte, the effect sizes are comparable between time points.

For each of the vitamins and related vitamers and metabolites, analyses focused on (i) describing the longitudinal changes in maternal concentrations from preconception through pregnancy and to 6 months postdelivery in the control group, (ii) the pattern of longitudinal change in the intervention group, and (iii) differences in concentration and in sufficiency markers between the control and intervention groups at different time points, including differences in proportions analyzed using chi-squared tests. Longitudinal plots are shown as means and 95% confidence intervals (CI; calculated using the exact method for continuous outcomes and binomial exact method for proportions) in SDS by intervention group on y-axis and time point/visit on x-axis, with statistical significance considered when the 95% CI did not overlap (two-tailed *p* < 0.05). Differences given in the text are statistically significant unless otherwise stated. Plots and differences in SDS between time points are based on all available data at each time point unless otherwise specified. Linear regression sensitivity analyses of the control versus intervention group differences were undertaken with adjustment for site and ethnicity (trial randomization stratification factors) and parity (not fully balanced across control and intervention groups and potentially influential on status measurements). Further sensitivity analyses were used to determine whether differences between time points reflected differences between those who did and did not become pregnant (resulting in differing numbers of participants with preconception, pregnancy, and postdelivery measurements). Analyses were performed using Stata software v15.1 (StataCorp, College Station, Texas, United States of America).

## Results

Characteristics of the participants with preconception data (*n* = 1,727) and of those who had baseline analyte data and were followed up at late pregnancy (*n* = 580) are shown in Table 1. Characteristics were balanced across the control and intervention groups, except for somewhat more nulliparity among controls; characteristics for those followed up through pregnancy to postdelivery (*n* = 512) were similar to those with preconception data, both overall and across the 3 sites (S1 Table).

**Table 1:** Participant characteristics.

		Preconception		Baseline and late pregnancy^+^	
Characteristic		Control (*n* = 857)	Intervention (*n* = 870)	Control (*n* = 287)	Intervention (*n* = 293)
*Sociodemographic*					
Study site	UK	229 (26.7%)	232 (26.7%)	92 (32.1%)	97 (33.1%)
	Singapore	328 (38.3%)	332 (38.2%)	82 (28.6%)	84 (28.7%)
	New Zealand	300 (35.0%)	306 (35.2%)	113 (39.4%)	112 (38.2%)
Age, years	Mean (SD)	30.6 (3.7)	30.6 (3.7)	30.2 (3.3)	30.5 (3.4)
Ethnicity	White	396 (46.2%)	413 (47.5%)	165 (57.5%)	179 (61.1%)
	Chinese	220 (25.7%)	238 (27.4%)	73 (25.4%)	72 (24.6%)
	South Asian	60 (7.0%)	61 (7.0%)	15 (5.2%)	15 (5.1%)
	Malay	85 (9.9%)	80 (9.2%)	12 (4.2%)	11 (3.8%)
	Other	96 (11.2%)	78 (9.0%)	22 (7.7%)	16 (5.5%)
Household income^#^	Low income	74 (9.3%)	76 (9.4%)	11 (4.0%)	12 (4.3%)
	Middle income	344 (43.2%)	354 (43.7%)	114 (41.2%)	114 (40.9%)
	High income	378 (47.5%)	380 (46.9%)	152 (54.9%)	153 (54.8%)
*Gynecological*					
Parity	Nulliparous	593 (69.2%)	578 (66.4%)	196 (68.3%)	169 (57.7%)
	Parous	264 (30.8%)	292 (33.6%)	91 (31.7%)	124 (42.3%)
*Lifestyle*					
Alcohol intake (per week)	None	271 (31.6%)	265 (30.5%)	61 (21.3%)	65 (22.2%)
	>0 to ≤2.5 units	312 (36.4%)	301 (34.6%)	115 (40.1%)	109 (37.2%)
	>2.5 units	274 (32.0%)	304 (34.9%)	111 (38.7%)	119 (40.6%)
Smoking status	Never	664 (77.7%)	662 (76.3%)	225 (78.7%)	237 (80.9%)
	Previous	131 (15.3%)	136 (15.7%)	49 (17.1%)	44 (15.0%)
	Active	60 (7.0%)	70 (8.1%)	12 (4.2%)	12 (4.1%)
Instances of moderate/vigorous physical activity in past 7 days	Median (IQR)	3 (2, 5)	3 (1, 5)	4 (2, 6)	3 (2, 5)
BMI category^##^	Not overweight or obese	400 (46.7%)	432 (49.8%)	158 (55.1%)	163 (55.8%)
	Overweight	240 (28.0%)	224 (25.8%)	69 (24.0%)	89 (30.5%)
	Obese	216 (25.2%)	212 (24.4%)	60 (20.9%)	40 (13.7%)
Days between preconception baseline and post-supplementation sampling	Median (IQR)	28 (23, 31)	28 (23, 32)	27 (22, 31)	28 (23, 31)
Preconception baseline: Taking a multiple micronutrient supplement	No	583 (68.6%)	609 (70.2%)	198 (69.0%)	195 (66.6%)
	Yes	267 (31.4%)	259 (29.8%)	89 (31.0%)	98 (33.4%)

Data presented as number (%) unless otherwise stated. Sample sizes do not always equal to 857/251 for control group and 870/261 for intervention group due to missing values.

^+^Based on all available data for individuals with any analyte at both baseline and late pregnancy.

^#^Low 1st–3rd decile, Middle 4th–7th decile, High 8th–10th decile.

^##^BMI categories: Not overweight or obese, Overweight, and Obese <25, 25–<30, and ≥30 kg/m^2^, respectively.

BMI, body mass index; IQR, interquartile range; SD, standard deviation.

**Fig 1:**
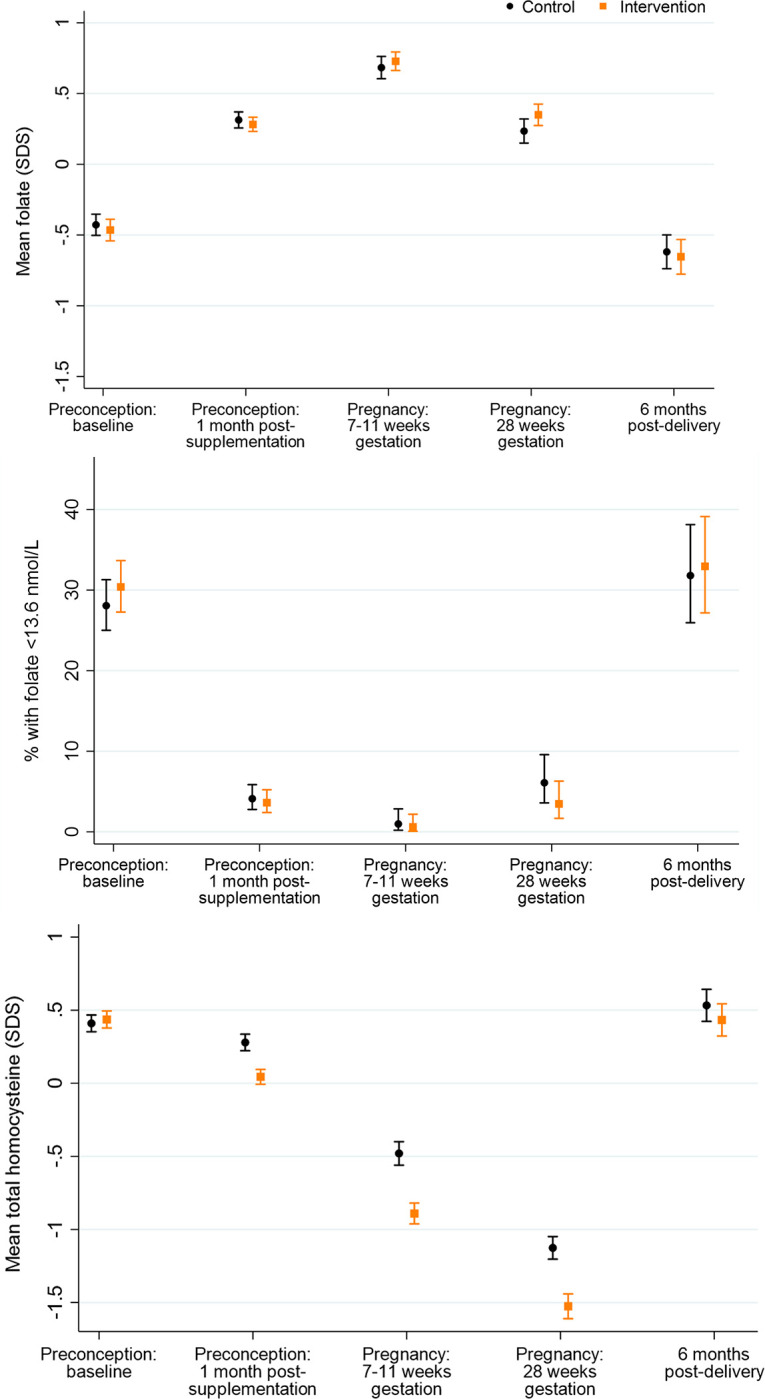
Plasma folate, marginal folate status, and plasma homocysteine according to time point and control/intervention group. Footnote to Fig 1: Folate *n* = 816/826, 705/746, 305/328, 279/288, 239/252 and homocysteine *n* = 854/867, 707/747, 305/329, 288/293, 251/261, for preconception baseline, preconception 1 month post-supplementation, early pregnancy, late pregnancy, and 6 months postdelivery, respectively.

**Fig 2:**
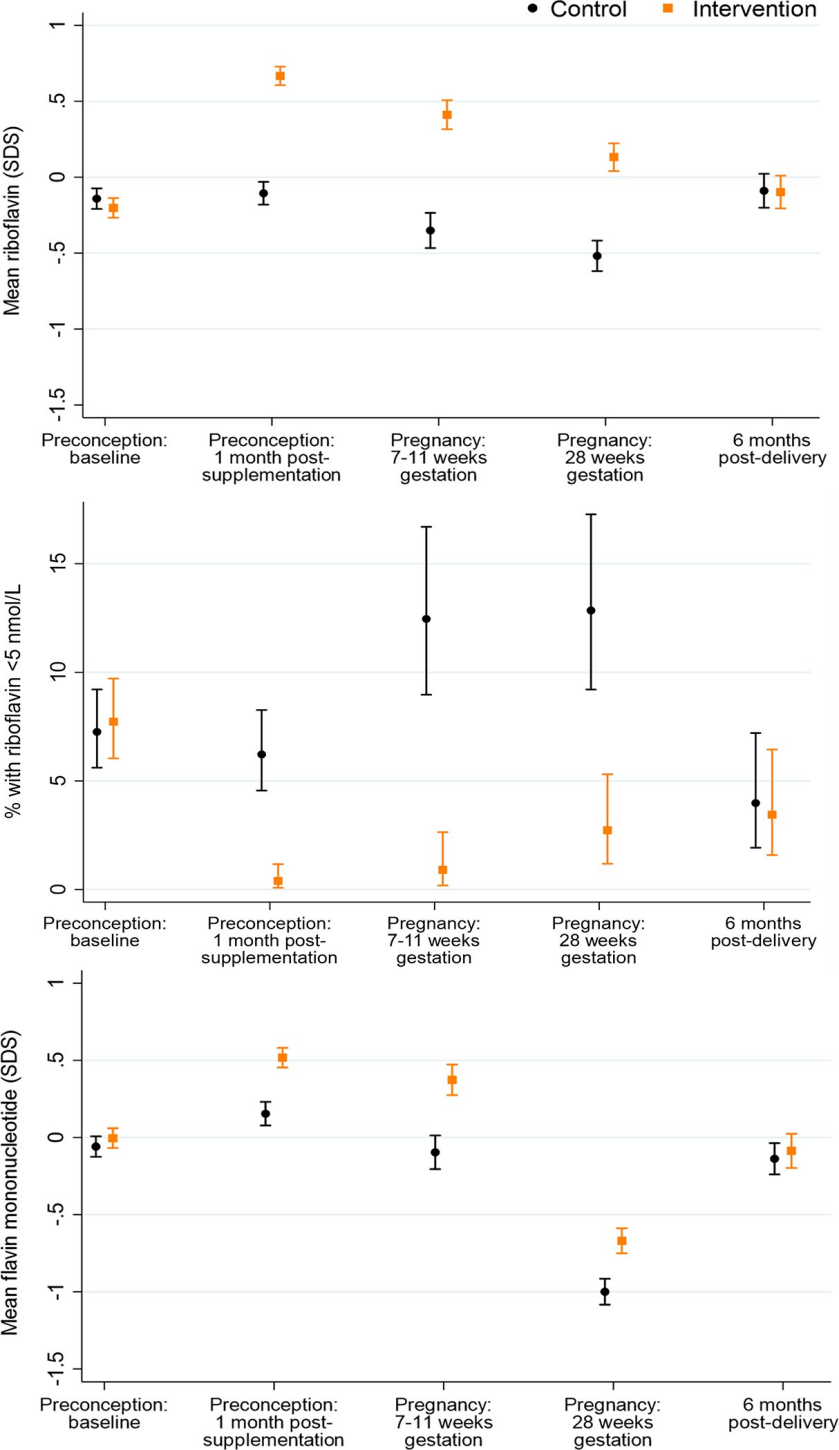
Plasma riboflavin, low riboflavin, and plasma flavin mononucleotide according to time point and control/intervention group. Footnote to Fig 2: Riboflavin and flavin mononucleotide *n* = 854/867, 707/747, 305/329, 288/293, 251/261 for preconception baseline, preconception 1 month post-supplementation, early pregnancy, late pregnancy, and 6 months postdelivery, respectively.

**Fig 3:**
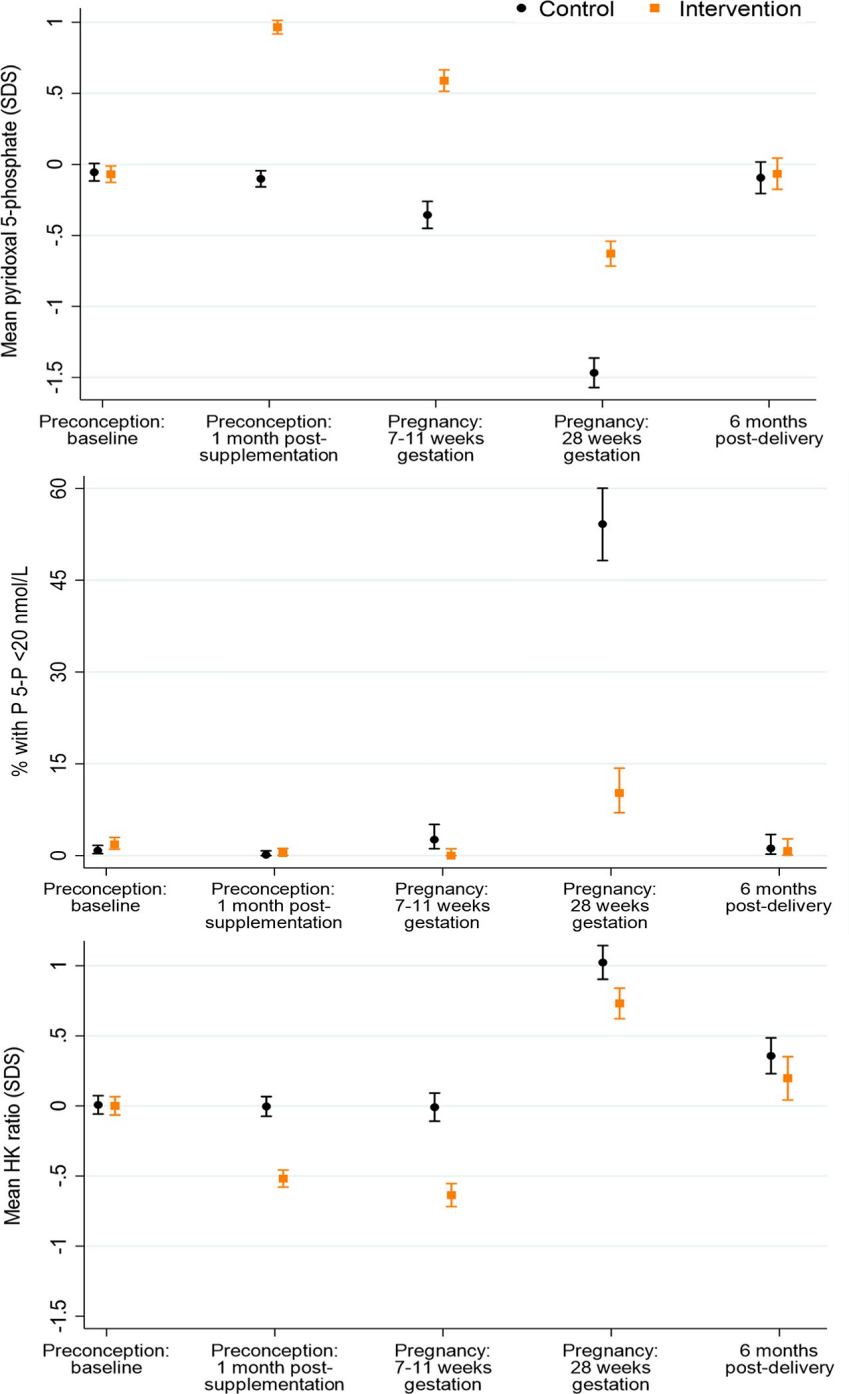
Plasma pyridoxal 5-phosphate (P-5-P), low pyridoxal 5-phosphate and, plasma 3-hydroxyknurenine (HK) ratio according to time point and control/intervention group. Footnote to Fig 3: P-5-P *n* = 854/867, 707/747, 305/329, 288/293, 251/261 and HK ratio *n* = 735/746, 692/724, 303/322, 261/270, 217/231, for preconception baseline, preconception 1 month post-supplementation, early pregnancy, late pregnancy, and 6 months postdelivery, respectively.

**Fig 4:**
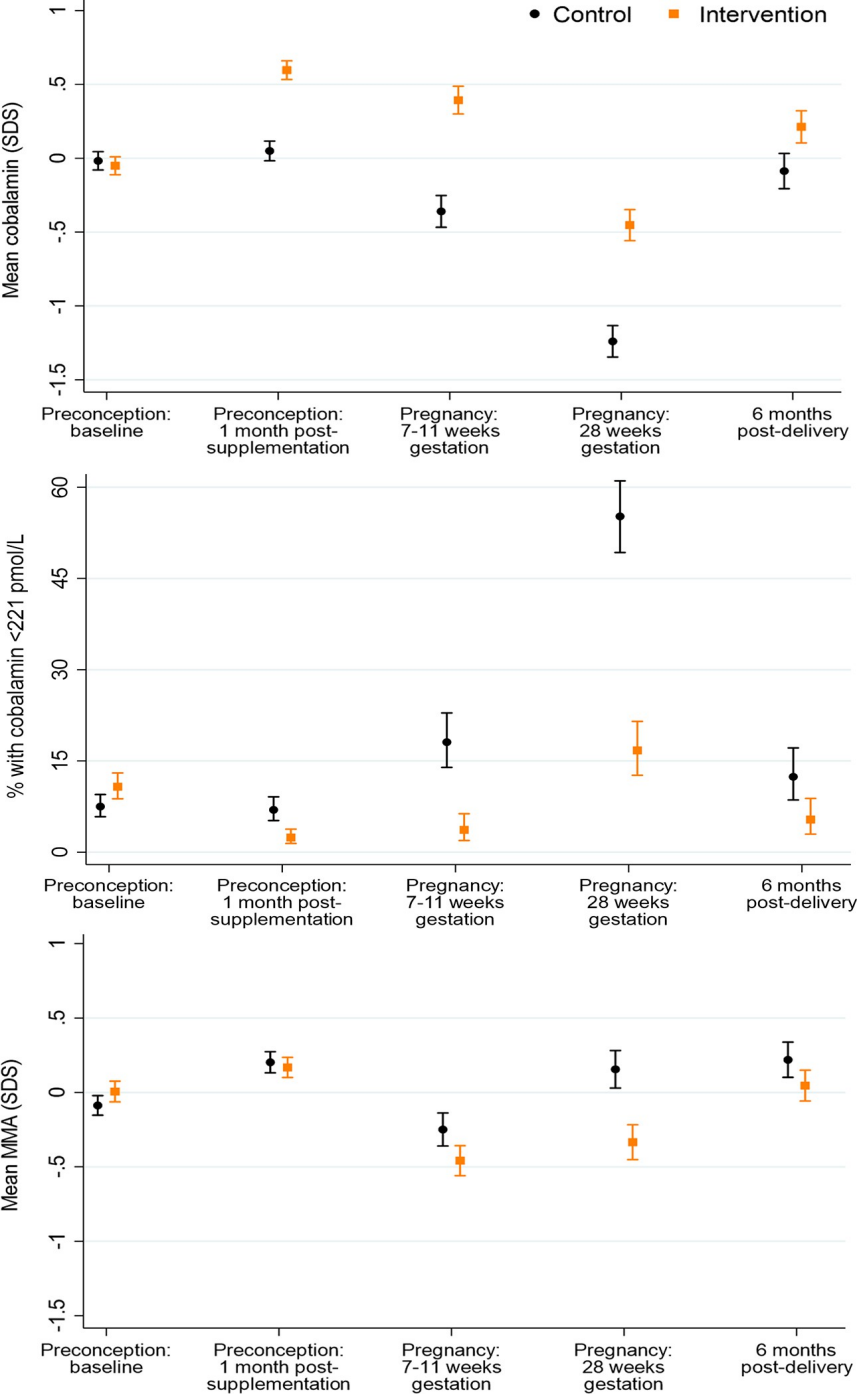
Plasma cobalamin, cobalamin depletion, and plasma methylmalonic acid (MMA) according to time point and control/intervention group. Footnote to Fig 4: cobalamin *n* = 853/864, 704/746, 304/327, 288/293, 250/261) and MMA *n* = 854/867, 707/747, 305/329, 288/293, 251/261 for preconception baseline, preconception 1 month post-supplementation, early pregnancy, late pregnancy, and 6 months postdelivery, respectively.

**Fig 5:**
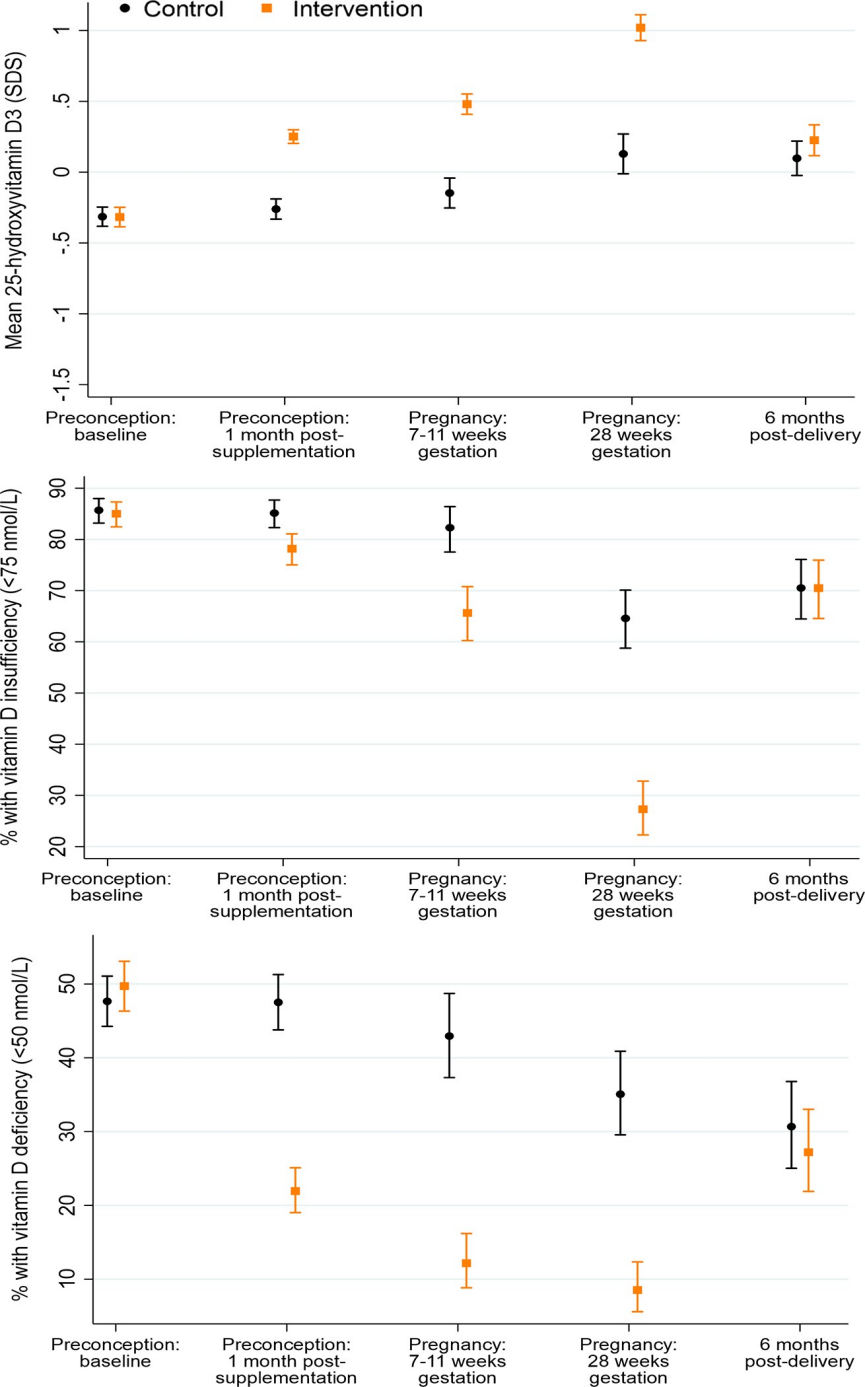
Plasma 25-hydroxyvitamin D, vitamin deficiency, and insufficiency according to time point and control/intervention group. Footnote to Fig 5: 25-hydroxyvitamin D *n* = 854/867, 707/747, 305/329, 288/293, 251/261 for preconception baseline, preconception 1 month post-supplementation, early pregnancy, late pregnancy, and 6 months postdelivery, respectively.

Table 2 shows median and interquartile range values for the maternal plasma vitamins and related vitamers/metabolites in their original units, and Table 3 the percentages of participants below vitamin and vitamer deficiency/insufficiency marker thresholds, according to time point and control/intervention group, with the longitudinal changes (as SDS and proportions with marginal or low status, depletion, deficiency, and insufficiency) shown graphically in Figs 1–5. Among all participants at recruitment preconception, significant proportions had marginal or low plasma status for folate (29.2% <13.6 nmol/L), riboflavin (7.5% <5 nmol/L, 82.0% ≤26.5 nmol/L), vitamin B12 (9.1% <221 pmol/L), and vitamin D (48.7% <50 nmol/L); at recruitment, over 91% of participants had low or marginal status of one or more of these vitamins. Only 1.3% had a low pyridoxal 5-phosphate (<20 nmol/L).

**Table 2:** Median (IQR) plasma concentrations in original units according to control or intervention group at each time point.

	Preconception baseline		Preconception 1 month post-supplementation		Early pregnancy (7–11 weeks gestation)		Late pregnancy (28 weeks gestation)		6 months postdelivery	
	Controls (*n* = 735–854)	Intervention (*n* = 746–867)	Controls (*n* = 692–707)	Intervention (*n* = 724–747)	Controls (*n* = 303–305)	Intervention (*n* = 322–329)	Controls (*n* = 261–288)	Intervention (*n* = 270–293)	Controls (*n* = 217–251)	Intervention (*n* = 231–261)
Folate (nmol/L)	22.5 (12.7, 41.3)	20.5 (12.1, 41.3)	41.9 (29.3, 55.0)	40.3 (29.0, 52.8)	49.8 (40.5, 60.0)	52.2 (43.2, 63.0)	41.5 (30.6, 51.6)	44.1 (34.3, 54.5)	19.2 (11.9, 32.7)	18.9 (12.3, 32.2)
Homocysteine (μmol/L)	7.0 (6.1, 8.4)	7.1 (6.1, 8.5)	6.9 (6.0, 8.0)	6.4 (5.6, 7.4)	5.5 (4.8, 6.4)	4.9 (4.2, 5.6)	4.6 (3.9, 5.2)	3.9 (3.5, 4.6)	7.4 (6.4, 9.0)	7.2 (6.1, 8.5)
Riboflavin (nmol/L)	12.7 (7.7, 21.7)	12.0 (7.6, 21.2)	12.7 (8.3, 22.3)	24.7 (16.7, 36.8)	10.7 (6.8, 18.0)	20.0 (13.0, 30.3)	10.3 (6.6, 15.1)	16.9 (12.2, 23.3)	12.9 (8.8, 21.1)	12.7 (8.4, 22.6)
FMN (nmol/L)	14.4 (11.5, 18.7)	14.6 (11.9, 18.7)	15.6 (12.4, 20.1)	18.4 (15.0, 23.4)	14.5 (11.4, 18.2)	17.2 (14.3, 21.6)	10.1 (8.4, 12.0)	11.5 (9.8, 13.4)	14.2 (11.8, 17.0)	14.4 (11.2, 18.7)
Pyridoxal 5-phosphate (nmol/L)	55.8 (40.3, 88.4)	57.1 (41.1, 84.3)	56.9 (43.0, 80.1)	140.0 (102.0, 184.0)	47.6 (34.3, 64.1)	106.0 (76.0, 139.0)	19.1 (14.7, 28.1)	41.9 (30.3, 56.4)	56.3 (40.0, 88.2)	56.1 (40.5, 86.6)
HK ratio (no units)	0.35 (0.29, 0.43)	0.36 (0.29, 0.42)	0.35 (0.29, 0.42)	0.29 (0.24, 0.35)	0.35 (0.28, 0.43)	0.29 (0.24, 0.33)	0.51 (0.41, 0.60)	0.44 (0.37, 0.54)	0.40 (0.32, 0.49)	0.39 (0.32, 0.47)
Cobalamin (pmol/L)	355.2 (283.1, 434.1)	350.9 (278.8, 437.7)	358.9 (284.0, 449.3)	438.2 (353.3, 552.1)	307.4 (240.2, 384.0)	411.1 (332.0, 509.3)	214.6 (177.3, 270.0)	298.7 (247.9, 379.5)	340.4 (264.5, 437.8)	385.6 (312.3, 465.7)
MMA (μmol/L)	0.13 (0.11, 0.17)	0.13 (0.11, 0.17)	0.15 (0.12, 0.19)	0.15 (0.12, 0.19)	0.12 (0.10, 0.15)	0.11 (0.10, 0.14)	0.14 (0.11, 0.20)	0.12 (0.10, 0.15)	0.15 (0.12, 0.20)	0.14 (0.12, 0.17)
Vitamin D3 (nmol/L)	51.0 (36.0, 65.3)	50.1 (36.1, 65.8)	51.7 (37.0, 65.5)	61.1 (51.1, 73.3)	53.3 (41.8, 69.5)	69.6 (57.3, 81.2)	62.9 (41.0, 87.5)	92.8 (73.0, 106.9)	62.5 (45.3, 77.6)	64.6 (48.5, 77.6)

FMN, flavin mononucleotide; HK ratio, 3′-hydroxykynurenine ratio; IQR, interquartile range; MMA, methylmalonic acid.

**Table 3:** Percentages of participants meeting vitamin/vitamer deficiency/insufficiency thresholds according to time point and control/intervention group.

	Preconception baseline		Preconception 1 month post supplementation		Early pregnancy (7–11 weeks gestation)		Late pregnancy (28 weeks gestation)		6 months postdelivery	
	Controls (*n* = 816–854)	Intervention (*n* = 826–867)	Controls (*n* = 704–707)	Intervention (*n* = 746–747)	Controls (*n* = 304–305)	Intervention (*n* = 327–329)	Controls (*n* = 279–288)	Intervention (*n* = 288–293)	Controls (*n* = 239–251)	Intervention (*n* = 252–261)
Folate <13.6 nmol/L	28.1% [25.0, 31.3]	30.4% [27.3, 33.7]	4.1% [2.8, 5.9]	3.6% [2.4, 5.2]	1.0% [0.2, 2.8]	0.6% [0.1, 2.2]	6.1% [3.6, 9.6]	3.5% [1.7, 6.3]	31.8% [25.9, 38.1]	32.9% [27.2, 39.1]
Homocysteine ≥15 μmol/L	0.9% [0.4, 1.8]	0.8% [0.3, 1.7]	0.7% [0.2, 1.6]	0.1% [0.0, 0.7]	0.0% [0.0, 1.2]	0.0% [0.0, 1.1]	0.0% [0.0, 1.3]	0.0% [0.0, 1.3]	0.4% [0.0, 2.2]	2.3% [0.8, 4.9]
Riboflavin <5 nmol/L	7.3% [5.6, 9.2]	7.7% [6.0, 9.7]	6.2% [4.6, 8.3]	0.4%* [0.1, 1.2]	12.5% [9.0, 16.7]	0.9%* [0.2, 2.6]	12.8% [9.2, 17.3]	2.7%* [1.2, 5.3]	4.0% [1.9, 7.2]	3.4% [1.6, 6.4]
Riboflavin ≤26.5 nmol/L	81.1% [78.4, 83.7]	82.8% [80.1, 85.3]	80.3% [77.2, 83.2]	56.1%* [52.4, 59.7]	85.6% [81.1, 89.3]	66.9%* [61.5, 71.9]	92.4% [88.7, 95.2]	81.9%* [77.0, 86.1]	82.5% [77.2, 87.0]	82.8% [77.6, 87.1]
Pyridoxal 5-phosphate <20 nmol/L	0.8% [0.3, 1.7]	1.8% [1.1, 3.0]	0.1% [0.0, 0.8]	0.4% [0.1, 1.2]	2.6% [1.1, 5.1]	0.0%* [0.0, 1.1]	54.2% [48.2, 60.0]	10.2%* [7.0, 14.3]	1.2% [0.2, 3.5]	0.8% [0.1, 2.7]
Cobalamin <148 pmol/L	0.6% [0.2, 1.4]	0.5% [0.1, 1.2]	0.6% [0.2, 1.4]	0.0% [0.0, 0.5]	1.6% [0.5, 3.8]	0.3% [0.0, 1.7]	11.1% [7.7, 15.3]	4.8%* [2.6, 7.9]	0.8% [0.1, 2.9]	0.4% [0.0, 2.1]
Cobalamin <221 pmol/L	7.5% [5.8, 9.5]	10.8%* [8.8, 13.0]	7.0% [5.2, 9.1]	2.4%* [1.4, 3.8]	18.1% [13.9, 22.9]	3.7%* [1.9, 6.3]	55.2% [49.3, 61.0]	16.7%* [12.6, 21.5]	12.4% [8.6, 17.1]	5.4%* [3.0, 8.8]
MMA >0.26 μmol/L	6.2% [4.7, 8.0]	8.7% [6.9, 10.7]	8.5% [6.5, 10.8]	8.6% [6.7, 10.8]	7.5% [4.8, 11.1]	3.6%* [1.9, 6.3]	11.8% [8.3, 16.1]	5.8%* [3.4, 9.1]	10.0% [6.5, 14.4]	5.4% [3.0, 8.8]
Vitamin D3 <50 nmol/L	47.7% [44.3, 51.1]	49.7% [46.3, 53.1]	47.5% [43.8, 51.3]	22.0%* [19.0, 25.1]	43.0% [37.3, 48.7]	12.2%* [8.8, 16.2]	35.1% [29.6, 40.9]	8.5%* [5.6, 12.3]	30.7% [25.0, 36.8]	27.2% [21.9, 33.0]
Vitamin D3 <75 nmol/L	85.7% [83.2, 88.0]	85.0% [82.5, 87.3]	85.1% [82.3, 87.7]	78.2%* [75.0, 81.1]	82.3% [77.5, 86.4]	65.7%* [60.2, 70.8]	64.6% [58.8, 70.1]	27.3%* [22.3, 32.8]	70.5% [64.5, 76.1]	70.5% [64.6, 76.0]

Values in parentheses are 95% confidence intervals.

*Chi-squared *p* < 0.05 for intervention vs. control difference at that time point.

MMA, methylmalonic acid.

### Plasma folate and homocysteine

In the control group (who received 400 μg/day folic acid, as in the intervention group), from preconception baseline to 1 month after supplementation commencement mean maternal plasma folate increased by 0.74 SDS (95% CI: 0.64 to 0.84, *p* < 0.0001), followed by a further 0.37 (0.27 to 0.47, *p* < 0.0001) SDS increase in early pregnancy, and then declined 0.45 (0.33 to 0.56, *p* < 0.0001) SDS from early to late pregnancy and 0.85 (0.71 to 1.00, *p* < 0.0001) SDS from late pregnancy to 6 months postdelivery, resulting in a postdelivery concentration lower than at preconception baseline (Fig 1); marginal folate status (<13.6 nmol/L) was present in 28.1% of control group participants at preconception baseline, falling to 4.1% 1 month after supplementation commencement (95% CI for difference in proportions 20.5% to 27.4%, *p* < 0.0001) and 1.0% in early pregnancy, before rising to 6.1% in late pregnancy and 31.8% 6 months postdelivery (Fig 1). The longitudinal pattern was similar in the intervention group.

Plasma homocysteine concentrations, an indicator of folate and B-vitamin deficiency, in the control group decreased by 0.13 (0.05 to 0.21, *p* = 0.002) SDS from preconception baseline to 1 month after supplementation commencement, followed by further 0.76 (0.66 to 0.86, *p* < 0.0001) and 0.65 (0.53 to 0.76, *p* < 0.0001) SDS falls in early and late pregnancy, respectively, and then a 1.66 (1.53 to 1.79, *p* < 0.0001) SDS increase 6 months postdelivery, resulting in a postdelivery concentration marginally higher than at recruitment (Fig 1); plasma homocysteine was ≥15 μmol/L in 0.9% of participants at preconception baseline, falling to 0% in early pregnancy and late pregnancy, and 0.4% 6 months postdelivery. The intervention group showed a similar longitudinal pattern, but plasma homocysteine concentrations were 0.24 (0.16 to 0.31, *p* < 0.0001), 0.41 (0.30 to 0.52, *p* < 0.0001), and 0.40 (0.29 to 0.51, *p* < 0.0001) SDS lower than in the control group 1 month after supplementation commencement and in early and late pregnancy, respectively.

### Plasma riboflavin and flavin mononucleotide

Among control group participants (taking a supplement without riboflavin), as expected, plasma riboflavin was similar at preconception baseline and 1 month after supplementation commencement, then decreased by 0.24 (0.11 to 0.38, *p* = 0.0005) SDS in early pregnancy, with a further 0.17 (0.01 to 0.32, *p* = 0.03) SDS decrease in late pregnancy, followed by an increase of 0.43 (0.28 to 0.58, *p* < 0.0001) SDS at 6 months postdelivery, similar to the level at preconception (Fig 2). Marginal riboflavin status (≤26.5 nmol/L) was present in 81.1% of control participants at preconception baseline and 80.3% 1 month after supplementation commencement, increasing to 85.6% and 92.4% in early and late pregnancy, respectively, before returning to 82.5% 6 months postdelivery (S2 Fig). Low riboflavin status (<5 nmol/L) in the control group showed a similar longitudinal pattern at the expected lower prevalence (7.3%, 6.2%, 12.5%, 12.8%, and 4.0%, across the 5 time points) (Fig 2). Compared with the control group, plasma riboflavin concentrations in the intervention group 1 month after supplementation commencement, in early pregnancy, and in late pregnancy were higher (by 0.77 (0.68 to 0.87, *p* < 0.0001), 0.76 (0.61 to 0.91, *p* < 0.0001), and 0.65 (0.51 to 0.79, *p* < 0.0001) SDS, respectively), with lower prevalences of marginal (56.1%, 66.9%, and 81.9%, respectively) and low (0.4%, 0.9%, and 2.7%, respectively) riboflavin status.

Plasma flavin mononucleotide, a marker of riboflavin sufficiency, in the control group showed a similar longitudinal pattern to plasma riboflavin but increased by 0.21 (0.11 to 0.31, *p* < 0.0001) SDS from preconception baseline to 1 month after supplementation commencement, before showing a decline during pregnancy (Fig 2). Plasma flavin mononucleotide concentrations were 0.36 (0.26 to 0.46, *p* < 0.0001), 0.47 (0.32 to 0.62, *p* < 0.0001), and 0.33 (0.21 to 0.45, *p* < 0.0001) SDS higher in the intervention versus the control group 1 month after supplementation commencement, in early pregnancy, and in late pregnancy, respectively.

### Plasma vitamin B6 and B6 insufficiency markers

In the control group (taking a supplement without vitamin B6), plasma pyridoxal 5-phosphate was similar at preconception baseline and 1 month after supplementation commencement, then decreased by 0.25 (0.15 to 0.36, *p* < 0.0001) SDS between 1 month after supplementation commencement and early pregnancy, before a substantial further decrease of 1.11 (0.97 to 1.25, *p* < 0.0001) SDS from early pregnancy to late pregnancy, then a return to preconception concentrations 6 months postdelivery (Fig 3); preconception, low pyridoxal 5-phosphate (<20 nmol/L) was present in 0.8% of participants at baseline and 0.1% 1 month after supplementation commencement, increasing to 2.6% in early pregnancy and a steep rise to 54.2% in late pregnancy, before returning to 1.2% 6 months postdelivery (Fig 3). Compared with the control group, the intervention group had higher plasma pyridoxal 5-phosphate concentrations at 1 month after supplementation commencement, and in early and late pregnancy, by 1.07 (0.99 to 1.14, *p* < 0.0001), 0.95 (0.83 to 1.07, *p* < 0.0001), and 0.84 (0.70 to 0.97, *p* < 0.0001) SDS, respectively.

Among control participants, the plasma HK ratio was unchanged from the preconception baseline through 1 month after supplementation commencement and in early pregnancy, but then rose sharply by 1.03 (0.88 to 1.19, *p* < 0.0001) SDS between early and late pregnancy, reflecting reduced activity of vitamin B6–dependent pathways; between late pregnancy and 6 months postdelivery the HK ratio fell by 0.67 (0.49 to 0.84, *p* < 0.0001) SDS, to a level higher than the preconception baseline (Fig 3). Compared with the control group, in the intervention group, the plasma HK ratio was lower at 1 month after supplementation commencement, in early pregnancy, and in late pregnancy, by 0.51 (0.42 to 0.61, *p* < 0.0001), 0.63 (0.50 to 0.76, *p* < 0.0001), and 0.29 (0.13 to 0.45, *p* = 0.0004) SDS, respectively. In the control group, the plasma cystathionine/cysteine ratio increased from preconception baseline to 1 month after supplementation commencement, decreasing to a level slightly lower than baseline in early pregnancy, followed by a 1.36 (1.20 to 1.51, *p* < 0.0001) SDS rise between early and late pregnancy, and then a fall 6 months postdelivery to a level higher than the preconception baseline (S2 Fig). Similar to the HK ratio, compared with the control group, the plasma cystathionine/cysteine ratio was lower in the intervention group 1 month after supplementation commencement, in early pregnancy, and in late pregnancy, by 0.35 (0.25 to 0.45, *p* < 0.0001), 0.37 (0.21 to 0.53, *p* < 0.0001), and 0.23 (0.09 to 0.36, *p* = 0.001) SDS, respectively.

### Plasma vitamin B12 and methylmalonic acid

In the control group (taking a supplement without vitamin B12), plasma cobalamin was similar at preconception baseline and 1 month after supplementation commencement, then decreased by 0.41 (0.29 to 0.53, *p* < 0.0001) SDS in early pregnancy, further decreased by 0.88 (0.73 to 1.03, *p* < 0.0001) SDS in late pregnancy, then returning to baseline preconception concentrations 6 months postdelivery (Fig 4); preconception, vitamin B12 deficiency (<148 pmol/L) was present in 0.6% of participants at baseline and 1 month after supplementation commencement, increasing to 1.6% and 11.1% in early and late pregnancy, respectively, before returning to 0.8% 6 months postdelivery (S4 Fig). Vitamin B12 depletion (<221 pmol/L) in the control group showed a similar longitudinal pattern at the expected higher prevalence (7.5%, 7.0%, 18.1%, 55.2%, and 12.4%, across the 5 time points) (Fig 4). Compared with the control group, in the intervention group, plasma cobalamin concentrations 1 month after supplementation commencement, in early pregnancy, and in late pregnancy were higher (by 0.55 (0.46 to 0.64, *p* < 0.0001), 0.75 (0.61 to 0.90, *p* < 0.0001), and 0.79 (0.64 to 0.94, *p* < 0.0001) SDS, respectively), with lower prevalences of vitamin B12 deficiency (0%, 0.3%, and 4.8%, respectively) and of vitamin B12 depletion (2.4%, 3.7%, and 16.7%, respectively). Notably, plasma vitamin B12 was 0.3 (0.14 to 0.46, *p* = 0.0003) SDS higher in the intervention group than in the control group 6 months postdelivery, with a lower prevalence of vitamin B12 depletion (5.4% versus 12.4%).

Plasma methylmalonic acid, a metabolic indicator of vitamin B12 insufficiency, in the control group increased by 0.29 (0.19 to 0.39, *p* < 0.0001) SDS from preconception baseline to 1 month after supplementation commencement, decreasing by 0.45 (0.32 to 0.58, *p* < 0.0001) SDS in early pregnancy before increasing 0.40 (0.24 to 0.57, *p* < 0.0001) SDS in late pregnancy and a similar level 6 months postdelivery, 0.31 (0.17 to 0.44, *p* < 0.0001) SDS above the original preconception baseline value (Fig 4). Plasma methylmalonic acid concentrations in the intervention group were similar to those in the control group 1 month after supplementation commencement, but 0.21 (0.06 to 0.36, *p* = 0.006) and 0.49 (0.32 to 0.66, *p* < 0.0001) SDS lower in early pregnancy and in late pregnancy, respectively; 6 months postdelivery, there was a 0.17 (0.02 to 0.33, *p* = 0.03) SDS lower concentration in the intervention group.

### Plasma 25-hydroxyvitamin D

In the control group, plasma 25-hydroxyvitamin D changed little from preconception baseline to 1 month after supplementation commencement and early pregnancy, then increased by 0.28 (0.10 to 0.45, *p* = 0.002) SDS between early and late pregnancy, remaining similar to late pregnancy concentrations 6 months postdelivery (Fig 5). Vitamin D deficiency <50 nmol/L in the control group showed a similar pattern, with high prevalences at preconception baseline, 1 month after supplementation commencement and in early pregnancy (47.7%, 47.5%, and 43.0%, respectively), and rather lower prevalences in late pregnancy (35.1%) and 6 months postdelivery (30.7%) (Fig 5); vitamin D insufficiency <75 nmol/L showed a similar longitudinal pattern at high prevalence levels (85.7%, 85.1%, 82.3%, 64.6%, and 70.5%, across the 5 time points) (Fig 5). Compared with the control group, in the intervention group, plasma 25-hydroxyvitamin D concentrations 1 month after supplementation commencement, in early pregnancy, and in late pregnancy were higher (by 0.51 (0.43 to 0.60, *p* < 0.0001), 0.63 (0.50 to 0.75, *p* < 0.0001), and 0.89 (0.72 to 1.06, *p* < 0.0001) SDS, respectively), with prevalences of vitamin D deficiency (22.0%, 12.2%, and 8.5%, respectively) and insufficiency (78.2%, 65.7%, and 27.3%, respectively) substantially lower than the control group; 6 months postdelivery plasma 25-hydroxyvitamin D concentrations were similar in the intervention and control groups (Fig 5).

### Sensitivity analyses

Sensitivity analyses, performing linear regressions of globally standardized analytes against control/intervention group, showed that group differences were robust to adjustment for site, ethnicity, and parity (Table 4). Further sensitivity analyses of participants with complete preconception, pregnancy, and postdelivery data the patterns described above for each of the vitamins and vitamers/metabolites were confirmed as little different, with no substantive differences from the analyses of all participants with measurements at any time point (S2 Table) and with similar patterns across the 3 study sites (S3 Table).

**Table 4:** Linear regressions of globally standardized analytes against intervention/control group, adjusted for site, ethnicity, and parity.

Analyte	Preconception baseline				Preconception 1 month post-supplementation				Early pregnancy (7–11 weeks gestation)				Late pregnancy (28 weeks gestation)				6 months postdelivery			
	n	beta	*p*-value	95% CI	n	beta	*p*-value	95% CI	n	beta	*p*-value	95% CI	n	beta	*p*-value	95% CI	n	beta	*p*-value	95% CI
Folate	1,642	−0.048	0.357	(−0.15, 0.05)	1,451	−0.033	0.381	(−0.11, 0.04)	633	0.059	0.243	(−0.04, 0.16)	567	0.123	0.029	(0.01, 0.23)	491	−0.045	0.594	(−0.21, 0.12)
Homocysteine	1,721	0.033	0.419	(−0.05, 0.11)	1,454	−0.232	<0.0001	(−0.30, −0.16)	634	−0.406	<0.0001	(−0.51, -0.30)	581	−0.401	<0.0001	(−0.52, −0.29)	512	−0.101	0.188	(−0.25, 0.05)
Riboflavin	1,721	−0.063	0.181	(−0.16, 0.03)	1,454	0.775	<0.0001	(0.68, 0.87)	634	0.783	<0.0001	(0.64, 0.93)	581	0.656	<0.0001	(0.52, 0.79)	512	−0.012	0.877	(−0.17, 0.14)
FMN	1,721	0.055	0.232	(−0.04, 0.15)	1,454	0.368	<0.0001	(0.27, 0.47)	634	0.485	<0.0001	(0.34, 0.63)	581	0.336	<0.0001	(0.22, 0.45)	512	0.065	0.399	(−0.09, 0.22)
Pyridoxal 5-phosphate	1,721	−0.018	0.667	(−0.10, 0.06)	1,454	1.072	<0.0001	(1.00, 1.14)	634	0.974	<0.0001	(0.86, 1.09)	581	0.863	<0.0001	(0.73, 0.99)	512	0.036	0.649	(−0.12, 0.19)
HK ratio	1,481	−0.018	0.694	(−0.11, 0.07)	1,416	−0.52	<0.0001	(−0.61, −0.43)	625	−0.648	<0.0001	(−0.77, −0.53)	531	−0.339	<0.0001	(−0.49, −0.19)	448	−0.183	0.073	(−0.38, 0.02)
Cobalamin	1,717	−0.035	0.393	(−0.12, 0.05)	1,450	0.549	<0.0001	(0.46, 0.63)	631	0.776	<0.0001	(0.65, 0.91)	581	0.809	<0.0001	(0.67, 0.95)	511	0.303	<0.0001	(0.16, 0.45)
MMA	1,721	0.086	0.066	(−0.01, 0.18)	1,454	−0.04	0.4	(−0.13, 0.05)	634	−0.228	0.002	(−0.37, −0.09)	581	−0.509	<0.0001	(−0.68, −0.34)	512	−0.185	0.016	(−0.34, −0.03)
Vitamin D3	1,721	−0.028	0.49	(−0.11, 0.05)	1,454	0.496	<0.0001	(0.42, 0.57)	634	0.601	<0.0001	(0.49, 0.71)	581	0.877	<0.0001	(0.72, 1.04)	512	0.126	0.087	(−0.02, 0.27)

Positive beta values represent an xx higher SD in intervention group compared to control group, adjusting for site, ethnicity, and parity.

CI, confidence interval; FMN, flavin mononucleotide; HK ratio, 3′-hydroxykynurenine ratio; MMA, methylmalonic acid.

## Discussion

Among UK, Singapore, and New Zealand women attempting to become pregnant in this multicenter randomized controlled trial, significant proportions had marginal or low status of folate, riboflavin, vitamin B12, and vitamin D at recruitment preconception, and a high proportion developed markers of functional vitamin B6 deficiency in late pregnancy despite only a small proportion having a low vitamin B6 status preconception. Plasma concentrations for the above vitamins, related vitamers, and metabolic insufficiency markers showed differing patterns of change from preconception to pregnancy and 6 months postdelivery. In prespecified secondary analyses of the trial, a formulation enriched with micronutrients in amounts available in over-the-counter supplements commenced preconception and continued throughout pregnancy substantially increased plasma folate, riboflavin, and vitamins B6, B12, and D concentrations and reduced the prevalence of their deficiency/depletion markers before and during pregnancy, with a persisting benefit of higher plasma vitamin B12 to 6 months postdelivery.

As routinely recommended for preconception and pregnancy, supplementation with 400 μg folic acid daily in both the control and intervention groups raised plasma folate by >1 SDS from baseline to early pregnancy and reduced the prevalence of marginal folate status from 28.1% at baseline to 1.0% in early pregnancy. Our findings show that even with 400 μg, the lowest commonly marketed dose, there are appreciable improvements in folate status after 1 month and continued improvement into pregnancy. Plasma homocysteine in the control group fell substantially from preconception baseline to early pregnancy and again from early to late pregnancy, with greater falls in plasma homocysteine in the intervention group taking a supplement containing other micronutrients. While some have proposed that vitamin B6, riboflavin, and zinc might reduce homocysteine, there are data pointing away from this [28,29]. One previous review concluded that dietary folic acid reduced homocysteine levels by 25% and vitamin B12 produced an additional 7% reduction in blood homocysteine levels, whereas vitamin B6 had no significant additive effect [30]. Our findings support effects of both the folic acid (albeit based on pre-post comparison for folic acid) and the other micronutrients in the intervention group acting to improve 1-carbon status and reduce plasma homocysteine, with potential benefit for pregnancy and offspring outcomes [31,32].

In the control group, plasma riboflavin and flavin mononucleotide showed modest falls from preconception to early pregnancy, and again from early to late pregnancy. We considered measuring the gold standard erythrocyte glutathione reductase activation coefficient as a specific riboflavin deficiency marker, but resource to support this could not be secured. Detailed studies have identified ≤26.5 nmol/L as the change-point of plasma riboflavin below which the erythrocyte glutathione reductase activation coefficient becomes elevated [25]. Using this 26.5 nmol/L threshold, 82.0% of participants had a “marginal riboflavin status” at baseline, with 7.5% having a riboflavin concentration <5 nmol/L. In the intervention group, supplementation with 1.8 mg/day riboflavin, as in many prenatal supplements, substantially raised plasma riboflavin and reduced the prevalences of marginal and low riboflavin status before and during pregnancy. The implications of marginal or low riboflavin status before and during pregnancy have rarely been studied, but in a large cohort of pregnant women in Ireland and Northern Ireland, biomarker analysis showed that 68% had low or deficient riboflavin status, which was associated with a higher risk of anemia during pregnancy [33]; a similar association with anemia was also found among Malaysian and Canadian women [34].

Plasma pyridoxal 5-phosphate in the control group showed a modest fall from preconception to early pregnancy, but then a substantial fall from early to late pregnancy, accompanied by a sharp rise in plasma HK and cystathionine:cysteine ratios, reflecting impairment of vitamin B6–dependent pathways in late pregnancy. For vitamin B6, pyridoxal 5-phosphate is the most commonly used status marker [35]; the HK ratio, composed of HK and the 4 kynurenines that are products of the pyridoxal 5-phosphate–dependent enzymes kynurenine transaminase and kynureninase, has been developed as a marker of tryptophan catabolism regulation by vitamin B6 that rises in B6 deficiency [35,36]. Evaluation has also found that the ratio of cystathionine:cysteine (transsulfuration pathway regulation) has merit as a B6 intake marker, while being more subject to other influences than the HK ratio [22]. The marked late gestation rises in HK ratio is consistent with evidence of kynurenine pathway enzyme inhibition by the more than 10-fold physiological increase in estrogen with advancing gestation from mid to late pregnancy [37,38]. This late gestation inhibition of the kynurenine pathway may be partly physiological but nonetheless has implications for B6 nutrient supply to the fetus and potential impacts on later offspring health. Notably, there is increasing evidence linking childhood kynurenine pathway perturbations with metabolic health risk [39]. In our study, the intervention, providing 2.6 mg vitamin B6 daily, substantially increased plasma pyridoxal 5-phosphate levels and lowered the plasma HK ratio in late pregnancy, with a lowering of the cystathionine:cysteine ratio also supporting a lesser impairment of vitamin B6–dependent pathways. While the US Institute of Medicine Recommended Dietary Allowance for vitamin B6 in pregnancy is 1.9 mg/day [40], European Community recommendations for pregnancy are that vitamin B6 supplements of 2.5 to 4 mg/day are required to maintain the plasma concentration of vitamin B6 at pre-pregnancy levels [41].

In the control group, plasma vitamin B12 showed modest falls from preconception to early pregnancy, and a larger fall from early to late pregnancy, accompanied by increases in the late pregnancy prevalences of vitamin B12 deficiency and depletion (11.1% and 55.2%, respectively) and a rise in plasma methylmalonic acid as a marker of vitamin B12 insufficiency; this is in keeping with previous reports of raised vitamin B12 deficiency markers in late pregnancy [42]. Supplementation with 5.2 μg/day vitamin B12 led to higher plasma cobalamin and lower methylmalonic acid during pregnancy in the intervention group. Recommended daily allowance amounts for vitamin B12 vary internationally. Meta-analysis of randomized trials in nonpregnant individuals concluded that a doubling of vitamin B12 intake increases vitamin B12 concentrations by 14%, with a consistent benefit of amounts similar to the 5.2 μg/day used in this trial [43], whereas smaller amounts are less consistent in their effect on B12 status [44]. Systematic review has linked lower maternal vitamin B12 status with a variety of adverse pregnancy outcomes, including a higher risk of neural tube defects, recurrent pregnancy losses, gestational diabetes, pre-eclampsia, and lower birth weight [45], and meta-analysis has shown that lower plasma concentrations associate with a higher risk of preterm delivery [46]. Lower maternal concentrations are also associated with adverse cardiometabolic and neurocognitive outcomes in the offspring [45,47]. While randomized trials of vitamin B12 supplementation in pregnancy have been inconclusive in relation to an effect on birth weight, they support a beneficial effect on offspring neurocognitive development [45]. In our study, maternal plasma cobalamin was still higher in the intervention group postdelivery, 6 months after discontinuation of supplementation, most likely reflecting repletion of hepatic stores. It is likely this would increase breast milk vitamin B12 supply to the infant given the strong correlation between maternal plasma and breast milk concentrations [48]. This is potentially of importance as postdelivery maternal vitamin B12 supplementation with the low doses found in most over-the-counter supplements (1 to 10 μg/day) is widely thought to result in only modest increases in human milk levels [49].

Plasma 25-hydroxyvitamin D in the control group taking a supplement without vitamin D was remarkably unchanged from preconception baseline to early pregnancy, then increased modestly between early and late pregnancy, with high prevalences of vitamin D deficiency and insufficiency at baseline preconception (47.7% and 85.7%, respectively) and through pregnancy. Supplementation with 10 μg of vitamin D in the intervention group led to progressive increases in plasma 25-hydroxyvitamin D and a substantial reduction in the prevalences of vitamin D deficiency and insufficiency during pregnancy (e.g., 8.5% and 27.3% in late pregnancy, respectively, versus 35.1% and 64.6% in the control group). The dose of vitamin D recommended for routine use in pregnancy varies internationally, with some advocating 100 μg daily or higher [50], while the World Health Organization/Food and Agriculture Organization of the United Nations recommends 5 μg daily [51]; in the UK, it is recommended that pregnant women should consider taking a supplement containing 10 μg between September and March [4]. In the MAVIDOS randomized trial supplementation, 25 μg of vitamin D daily from 14 weeks gestation until delivery lowered the incidence of infantile atopic eczema [5] and increased childhood areal bone mineral density in the offspring [6]. Vitamin D is stored in adipose tissue, and our trial suggests that low dose (10 μg) supplementation over a long period starting preconception can support improved gestational vitamin D status.

The finding of significant prevalences of vitamin insufficiencies in women living in high-income countries who are attempting to become pregnant is a serious concern. This is particularly the case given increasing advocacy to reduce meat and dairy intakes to achieve “net-zero” carbon emissions, as these foods are micronutrient dense and support nutritional adequacy [52,53]. The high prevalence of vitamin insufficiencies and increasing move toward plant-based diets, which lack vitamin B12 and are low in other micronutrients, is likely to result in more women choosing over-the-counter supplements.

Local policy in high- and low-income settings can introduce the investigated supplement amounts as the amounts incorporated into the intervention supplement are available in over-the-counter multivitamin and single-vitamin supplements. As such, the effects we report on vitamin status before and during pregnancy are generalizable and fill an important gap in the literature. With the supplement used in our trial, some participants nonetheless had persisting evidence of low or marginal vitamin status, and our findings point to a need to build an evidence base to inform food fortification and biofortification policymaking beyond the current principal focus on folic acid for prevention of neural tube defects.

To date, changes in vitamin status from preconception to early and late pregnancy and postpartum have largely been inferred from cross-sectional data, with lower concentrations during pregnancy often ascribed to plasma volume expansion [12,13]. Our findings show, however, that the magnitude and pattern of change varies between nutrients, inconsistent with an effect wholly due to physiological hemodilution and that markers of functional B6 (HK ratio) and B12 (methylmalonic acid) insufficiency increase during pregnancy. Variations between individual vitamins in maternal metabolism, intake, renal loss, and feto-placental demands during pregnancy may all contribute to these varying patterns. Our randomized trial has shown for the first time that preconception and pregnancy supplementation including riboflavin, folic acid, and vitamins B6, B12, and D in amounts available in over-the-counter supplements can contribute to the reduction in vitamin insufficiencies during the preconception, pregnancy, and lactational periods. The potential benefits for pregnancy outcome and offspring health remain to be characterized. Randomized trial evidence that continued folic acid supplementation throughout pregnancy beyond the first trimester can have beneficial effects on child cognitive development points to the possibility of lasting health benefits for the offspring [32].

### Strengths and limitations

Strengths of our study include longitudinal plasma samples from preconception through pregnancy to 6 months postdelivery, an interventional component, a relatively large sample size, and inclusion of multiple ethnic groups. The robust conduct of this double-blind randomized controlled trial, which included prospectively collected data and minimization of residual confounding through randomization, with external oversight by an independent data monitoring and trial steering committee, is a strength. Over 96% of women showed good adherence defined *a priori* as supplement intake greater than 60% averaged from recruitment to delivery. A limitation is that the study was based on (prespecified) secondary outcomes of the trial. Even though recruitment occurred across 3 different countries with inclusion of multiple ethnicities, generalizability to the global population is limited by the lack of African and Amerindian women in particular. A further limitation, inherent in all preconception studies, is that the women who became pregnant are a subsample of those recruited and unmeasured aspects of their characteristics may differ from the original group; in our study, measured characteristic remained balanced between the control and intervention groups in those who became pregnant. The recruited population were generally healthy and well nourished, yet evidence recognized as indicating micronutrient insufficiency was widespread; whether some changes represent a normal physiological change in pregnancy or a true insufficiency awaits characterization of relations with pregnancy and offspring outcomes. Another strength is the use of an accredited clinical laboratory for analyses of clinical biomarkers. In this study, the entire sample set was analyzed for all analytes in a single laboratory including authentic labeled internal standards for each analyte providing high analytical precision. The analyses were carried out in continuous sample batches, with small variability between each batch. Finally, standardized scores were used in the regression models so that the strength of associations were comparable.

## Conclusions

Significant proportions of preconception women living in high-income countries have marginal or low status of folate, riboflavin, vitamin B12, and vitamin D, and many develop markers of vitamin B6 deficiency in late pregnancy. In the absence of the intervention supplement, maternal plasma concentrations show differing longitudinal patterns between vitamins from preconception to early and late pregnancy, inconsistent with plasma volume expansion wholly accounting for lower gestational concentrations, and markers of functional B6 and B12 insufficiency increase during pregnancy. Preconception and pregnancy supplementation in amounts available in over-the-counter supplements substantially reduced the prevalence of deficiency/depletion markers before and during pregnancy, and a higher maternal plasma vitamin B12 was maintained during the recommended lactational period. In the setting of increasing advocacy for more diets that are likely to be less nutrient dense, the findings suggest a need to reappraise dietary recommendations for preconception and pregnancy and to consider further the role of multiple micronutrient supplements in women living in higher-income countries.

## Supporting information

S1 CONSORT Checklist CONSORT 2010 checklist of information to include when reporting a randomized trial. (DOC)

S1 Table Participant characteristics split by site. (DOCX)

S2 Table Median (IQR) values in original units according to control or intervention group at each time point, for participants with measurements at each of preconception baseline and late pregnancy. (DOCX)

S3 Table Median (IQR) plasma concentrations in original units according to control or intervention group at each time point for each site analyzed separately. (DOCX)

S1 Fig Flowchart of study participants from assessment of eligibility, through randomization, conception, pregnancy, and postdelivery. Abbreviation: Cesarean, cesarean section delivery.
(TIF)

S2 Fig Marginal riboflavin status (plasma riboflavin ≤26.5 nmol/L) by control/intervention group from preconception baseline to 6 months postdelivery. Footnote to S2 Fig: *n* = 854/867, 707/747, 305/329, 288/293, 251/261 for preconception baseline, preconception 1 month post-supplementation, early pregnancy, late pregnancy, and 6 months postdelivery, respectively.
(TIF)

S3 Fig Plasma cystathionine/cysteine ratio by control/intervention group from preconception baseline to 6 months postdelivery. Footnote to S3 Fig: *n* = 851/864, 707/747, 305/329, 288/293, 251/261 for preconception baseline, preconception 1 month post-supplementation, early pregnancy, late pregnancy, and 6 months postdelivery, respectively.
(TIF)

S4 Fig Vitamin B12 deficiency (<148 pmol/L) by control/intervention group from preconception baseline to 6 months postdelivery. Footnote to S4 Fig: *n* = 853/864, 704/746, 304/327, 288/293, 250/261 for preconception baseline, preconception 1 month post-supplementation, early pregnancy, late pregnancy, and 6 months postdelivery, respectively.
(TIF)

S1 Statistical Analyses Plan NiPPeR–Maternal Micronutrients. (PDF)

S1 Study protocol Nutritional Intervention Preconception and During Pregnancy to Maintain Healthy Glucose Metabolism and Offspring Health (“NiPPeR”): Study protocol for a randomized controlled trial. (PDF)

## Abbreviations

AA: anthranilic acid
BMI: body mass index
CI: confidence interval
HAA: hydroxyanthranilic acid
HK: 3-hydroxykynurenine
KA: kynurenic acid
MAVIDOS: Maternal Vitamin D Osteoporosis Study
SDS: standard deviation score
XA: 3-xanthurenic acid
