# Supplementary material for: Maternal B-vitamin and vitamin D status before, during, and after pregnancy and the influence of supplementation preconception and during pregnancy: Prespecified secondary analysis of the NiPPeR double-blind randomized controlled trial
Source: PLoS Med. 2023 Dec 5;20(12):e1004260. doi: 10.1371/journal.pmed.1004260 (PMC10697591; doi:10.1371/journal.pmed.1004260)
Supplement: S1 Table — (DOCX) [file pmed.1004260.s002.docx]

**Supplementary Table 1. Participant characteristics split by site**

| **UK only** |  | Preconception | | Baseline and late pregnancy^+^ | |
| --- | --- | --- | --- | --- | --- |
| Characteristic |  | Control (n=229) | Intervention (n=232) | Control (n=92) | Intervention (n=97) |
| *Sociodemographic* |  |  |  |  |  |
| Age, years | Mean (SD) | 30.1 (4.0) | 30.1 (4.0) | 29.6 (3.9) | 29.9 (3.7) |
|  |  |  |  |  |  |
| Ethnicity | White | 213 (93.0%) | 220 (94.8%) | 86 (93.5%) | 93 (95.9%) |
|  | Chinese | 1 (0.4%) | 0 (0%) | 0 (0%) | 0 (0%) |
|  | South Asian | 5 (2.2%) | 6 (2.6%) | 3 (3.3%) | 1 (1.0%) |
|  | Malay | 1 (0.4%) | 0 (0%) | 1 (1.1%) | 0 (0%) |
|  | Other | 9 (3.9%) | 6 (2.6%) | 2 (2.2%) | 3 (3.1%) |
|  |  |  |  |  |  |
| Household income^#^ | Low income | 8 (3.6%) | 10 (4.4%) | 3 (3.4%) | 3 (3.1%) |
|  | Middle income | 69 (30.9%) | 72 (31.7%) | 27 (30.3%) | 27 (27.8%) |
|  | High income | 146 (65.5%) | 145 (63.9%) | 59 (66.3%) | 67 (69.1%) |
| *Gynecological* |  |  |  |  |  |
| Parity | Nulliparous | 152 (66.4%) | 149 (64.2%) | 63 (68.5%) | 58 (59.8%) |
|  | Parous | 77 (33.6%) | 83 (35.8%) | 29 (31.5%) | 39 (40.2%) |
|  |  |  |  |  |  |
| *Lifestyle* |  |  |  |  |  |
| Alcohol intake (per week) | None | 40 (17.5%) | 33 (14.2%) | 13 (14.1%) | 16 (16.5%) |
|  | >0 to ≤2.5 units | 74 (32.3%) | 73 (31.5%) | 31 (33.7%) | 28 (28.9%) |
|  | >2.5 units | 115 (50.2%) | 126 (54.3%) | 48 (52.2%) | 53 (54.6%) |
|  |  |  |  |  |  |
| Smoking status | Never | 154 (67.5%) | 150 (64.9%) | 64 (69.6%) | 61 (62.9%) |
|  | Previous | 48 (21.1%) | 62 (26.8%) | 22 (23.9%) | 30 (30.9%) |
|  | Active | 26 (11.4%) | 19 (8.2%) | 6 (6.5%) | 6 (6.2%) |
|  |  |  |  |  |  |
| Instances of moderate | Median (IQR) | 3 (1,5) | 3 (2,5) | 4 (2,5.5) | 3 (1,5) |
| /vigorous physical activity in past 7 days |  |  |  |  |  |
|  |  |  |  |  |  |
| Body mass index (BMI) category^##^ | Not overweight or obese | 99 (43.2%) | 105 (45.3%) | 44 (47.8%) | 46 (47.4%) |
|  | Overweight | 68 (29.7%) | 72 (31.0%) | 23 (25.0%) | 32 (33.0%) |
|  | Obese | 62 (27.1%) | 55 (23.7%) | 25 (27.2%) | 19 (19.6%) |
|  |  |  |  |  |  |
| Days between preconception baseline and post-supplementation sampling | Median (IQR) | 29 (28, 34) | 29 (28,34) | 29 (28,34) | 29 (28,34) |
|  |  |  |  |  |  |
| Preconception baseline: Taking a multiple micronutrient supplement | No | 148 (64.9%) | 146 (62.9%) | 64 (69.6%) | 58 (59.8%) |
|  | Yes | 80 (35.1%) | 86 (37.1%) | 28 (30.4%) | 39 (40.2%) |

| **Singapore only** |  | Preconception | | Baseline and late pregnancy^+^ | |
| --- | --- | --- | --- | --- | --- |
| Characteristic |  | Control (n=328) | Intervention (n=332) | Control (n=82) | Intervention (n=84) |
| *Sociodemographic* |  |  |  |  |  |
| Age, years | Mean (SD) | 30.7 (3.6) | 30.8 (3.5) | 30.2 (3.0) | 30.5 (3.1) |
|  |  |  |  |  |  |
| Ethnicity | White | 0 (0%) | 0 (0%) | 0 (0%) | 0 (0%) |
|  | Chinese | 203 (61.9%) | 212 (63.9%) | 65 (79.3%) | 65 (77.4%) |
|  | South Asian | 28 (8.5%) | 27 (8.1%) | 5 (6.1%) | 7 (8.3%) |
|  | Malay | 84 (25.6%) | 80 (24.1%) | 11 (13.4%) | 11 (13.1%) |
|  | Other | 13 (4.0%) | 13 (3.9%) | 1 (1.2%) | 1 (1.2%) |
|  |  |  |  |  |  |
| Household income^#^ | Low income | 51 (16.7%) | 53 (16.6%) | 5 (6.4%) | 7 (8.6%) |
|  | Middle income | 175 (57.4%) | 179 (55.9%) | 45 (57.7%) | 51 (63.0%) |
|  | High income | 79 (25.9%) | 88 (27.5%) | 28 (35.9%) | 23 (28.4%) |
| *Gynecological* |  |  |  |  |  |
| Parity | Nulliparous | 222 (67.7%) | 214 (64.5%) | 49 (59.8%) | 37 (44.0%) |
|  | Parous | 106 (32.3%) | 118 (35.5%) | 33 (40.2%) | 47 (56.0%) |
|  |  |  |  |  |  |
| *Lifestyle* |  |  |  |  |  |
| Alcohol intake (per week) | None | 182 (55.5%) | 173 (52.1%) | 36 (43.9%) | 34 (40.5%) |
|  | >0 to ≤2.5 units | 118 (36.0%) | 127 (38.3%) | 38 (46.3%) | 45 (53.6%) |
|  | >2.5 units | 28 (8.5%) | 32 (9.6%) | 8 (9.8%) | 5 (6.0%) |
|  |  |  |  |  |  |
| Smoking status | Never | 287 (87.5%) | 276 (83.4%) | 76 (92.7%) | 79 (94.0%) |
|  | Previous | 21 (6.4%) | 29 (8.8%) | 5 (6.1%) | 1 (1.2%) |
|  | Active | 20 (6.1%) | 26 (7.9%) | 1 (1.2%) | 4 (4.8%) |
|  |  |  |  |  |  |
| Instances of moderate | Median (IQR) | 3 (1,5) | 2 (1, 4) | 3 (2,5) | 2 (1,4) |
| /vigorous physical activity in past 7 days |  |  |  |  |  |
|  |  |  |  |  |  |
| Body mass index (BMI) category^##^ | Not overweight or obese | 159 (48.5%) | 171 (51.8%) | 48 (58.5%) | 50 (60.2%) |
|  | Overweight | 82 (25.0%) | 82 (24.8%) | 18 (22.0%) | 29 (34.9%) |
|  | Obese | 87 (26.5%) | 77 (23.3%) | 16 (19.5%) | 4 (4.8%) |
|  |  |  |  |  |  |
| Days between preconception baseline and post-supplementation sampling | Median (IQR) | 28 (24, 32) | 28 (24, 34) | 27 (23, 31) | 27 (23, 33) |
|  |  |  |  |  |  |
| Preconception baseline: Taking a multiple micronutrient supplement | No | 234 (71.6%) | 257 (77.4%) | 61 (74.4%) | 63 (75.0%) |
|  | Yes | 93 (28.4%) | 75 (22.6%) | 21 (25.6%) | 21 (25.0%) |

| **New Zealand only** |  | Preconception | | Baseline and late pregnancy^+^ | |
| --- | --- | --- | --- | --- | --- |
| Characteristic |  | Control (n=300) | Intervention (n=306) | Control (n=113) | Intervention (n=112) |
| *Sociodemographic* |  |  |  |  |  |
| Age, years | Mean (SD) | 31.0 (3.5) | 30.8 (3.6) | 30.6 (3.0) | 31.0 (3.3) |
|  |  |  |  |  |  |
| Ethnicity | White | 183 (61.0%) | 193 (63.1%) | 79 (69.9%) | 86 (76.8%) |
|  | Chinese | 16 (5.3%) | 26 (8.5%) | 8 (7.1%) | 7 (6.3%) |
|  | South Asian | 27 (9.0%) | 28 (9.2%) | 7 (6.2%) | 7 (6.3%) |
|  | Malay | 0 (0%) | 0 (0%) | 0 (0%) | 0 (0%) |
|  | Other | 74 (24.7%) | 59 (19.3%) | 19 (16.8%) | 12 (10.7%) |
|  |  |  |  |  |  |
| Household income^#^ | Low income | 15 (5.6%) | 13 (4.9%) | 3 (2.7%) | 2 (2.0%) |
|  | Middle income | 100 (37.3%) | 103 (39.2%) | 42 (38.2%) | 36 (35.6%) |
|  | High income | 153 (57.1%) | 147 (55.9%) | 65 (59.1%) | 63 (62.4%) |
| *Gynecological* |  |  |  |  |  |
| Parity | Nulliparous | 219 (73.0%) | 215 (70.3%) | 84 (74.3%) | 74 (66.1%) |
|  | Parous | 81 (27.0%) | 91 (29.7%) | 29 (25.7%) | 38 (33.9%) |
|  |  |  |  |  |  |
| *Lifestyle* |  |  |  |  |  |
| Alcohol intake (per week) | None | 49 (16.3%) | 59 (19.3%) | 12 (10.6%) | 15 (13.4%) |
|  | >0 to ≤2.5 units | 120 (40.0%) | 101 (33.0%) | 46 (40.7%) | 36 (32.1%) |
|  | >2.5 units | 131 (43.7%) | 146 (47.7%) | 55 (48.7%) | 61 (54.5%) |
|  |  |  |  |  |  |
| Smoking status | Never | 223 (74.6%) | 236 (77.1%) | 85 (75.9%) | 97 (86.6%) |
|  | Previous | 62 (20.7%) | 45 (14.7%) | 22 (19.6%) | 13 (11.6%) |
|  | Active | 14 (4.7%) | 25 (8.2%) | 5 (4.5%) | 2 (1.8%) |
|  |  |  |  |  |  |
| Instances of moderate | Median (IQR) | 4 (2,6) | 3 (2,6) | 4 (3,6) | 4 (3,6) |
| /vigorous physical activity in past 7 days |  |  |  |  |  |
|  |  |  |  |  |  |
| Body mass index (BMI) category^##^ | Not overweight or obese | 142 (47.5%) | 156 (51.0%) | 66 (58.4%) | 67 (59.8%) |
|  | Overweight | 90 (30.1%) | 70 (22.9%) | 28 (24.8%) | 28 (25.0%) |
|  | Obese | 67 (22.4%) | 80 (26.1%) | 19 (16.8%) | 17 (15.2%) |
|  |  |  |  |  |  |
| Days between preconception baseline and post-supplementation sampling | Median (IQR) | 23 (21, 28) | 24 (21, 28) | 22 (21, 27) | 24 (21, 28) |
|  |  |  |  |  |  |
| Preconception baseline: Taking a multiple micronutrient supplement | No | 201 (68.1%) | 206 (67.8%) | 73 (64.6%) | 74 (66.1%) |
|  | Yes | 94 (31.9%) | 98 (32.2%) | 40 (35.4%) | 38 (33.9%) |

Data presented as number (%) unless otherwise stated. Sample sizes do not always equal to 857/251 for control group and 870/261 for intervention group due to missing values; + based on all available data for individuals with any analyte at both baseline and late pregnancy. #Low 1st-3rd decile, Middle 4th-7th decile, High 8th-10th decile; ##BMI categories - Not overweight or obese, Overweight and Obese <25, 25-<30 and ≥30 kg/m2, respectively. Abbreviations: IQR, interquartile range; SD, standard deviation
