# Supplementary material for: Maternal B-vitamin and vitamin D status before, during, and after pregnancy and the influence of supplementation preconception and during pregnancy: Prespecified secondary analysis of the NiPPeR double-blind randomized controlled trial
Source: PLoS Med. 2023 Dec 5;20(12):e1004260. doi: 10.1371/journal.pmed.1004260 (PMC10697591; doi:10.1371/journal.pmed.1004260)
Supplement: S2 Table — (DOCX) [file pmed.1004260.s003.docx]

**Supplementary Table 2: Median (IQR) values in original units according to control or intervention group at each time-point, for participants with measurements at each of preconception baseline and late pregnancy**

|  | Preconception baseline | | Preconception 1-month post supplementation | | Early pregnancy  (7-11 weeks gestation) | | Late pregnancy  (28 weeks gestation) | | 6 months post-delivery | |
| --- | --- | --- | --- | --- | --- | --- | --- | --- | --- | --- |
|  | Controls  (n=245-287) | Intervention  (n=257-293) | Controls  (n=281-286) | Intervention  (n=283-292) | Controls  (n=274-276) | Intervention  (n=284-289) | Controls  (n=260-287) | Intervention  (n=270-293) | Controls  (n=213-247) | Intervention  (n=230-260) |
| Folate  (nmol/L) | 23.1  (13.5, 41.3) | 23.8  (13.6, 42.6) | 44.4  (30.9, 56.3) | 42.9  (31.4, 54.2) | 49.9  (40.6, 60.2) | 52.2  (43.7, 63.0) | 41.5  (30.6, 51.4) | 44.1  (34.3, 54.5) | 19.3  (11.9, 33.2) | 19.0  (12.4, 32.4) |
| Homocysteine (µmol/L) | 7.0  (6.1, 8.3) | 7.1  (6.0, 8.4) | 6.8  (6.0, 7.9) | 6.3  (5.5, 7.3) | 5.5  (4.8, 6.4) | 4.8  (4.2, 5.5) | 4.6  (3.9, 5.2) | 3.9  (3.5, 4.6) | 7.4  (6.4, 9.0) | 7.2  (6.1, 8.5) |
| Riboflavin  (nmol/L) | 12.7  (7.5, 19.6) | 13.7  (8.6, 21.3) | 12.6  (8.4, 21.5) | 25.1  (16.8, 38.0) | 10.8  (6.7, 18.4) | 19.7  (13.0, 29.8) | 10.2  (6.6, 15.1) | 16.9  (12.2, 23.3) | 13.0  (8.9, 21.1) | 12.8  (8.4, 22.6) |
| FMN  (nmol/L) | 14.7  (11.4, 18.7) | 15.0  (12.0, 19.6) | 15.6  (12.7, 19.5) | 19.0  (15.1, 25.1) | 14.3  (11.4, 18.3) | 17.4  (14.2, 21.6) | 10.1  (8.4, 12.0) | 11.5  (9.8, 13.4) | 14.2  (11.8, 17.0) | 14.4  (11.2, 18.7) |
| Pyridoxal 5-phosphate (nmol/L) | 59.4 (42.4, 92.1) | 61.0  (44.9, 99.0) | 57.2  (44.7, 79.3) | 144  (107, 187) | 47.6  (34.7, 64.2) | 104.0  (75.8, 138.0) | 19.2  (14.6, 28.3) | 41.9  (30.3, 56.4) | 55.9  (40.0, 88.1) | 56.4  (40.5, 86.8) |
| HK ratio  (no units) | 0.35  (0.30, 0.43) | 0.36  (0.30, 0.41) | 0.36  (0.29, 0.42) | 0.29  (0.26, 0.34) | 0.35  (0.28, 0.42) | 0.29  (0.24, 0.33) | 0.51  (0.41,0.61) | 0.44 (0.37,0.54) | 0.40  (0.32, 0.49) | 0.39  (0.32, 0.47) |
| Cobalamin  (pmol/L) | 355  (276, 444) | 354  (278, 434) | 355  (278, 449) | 447  (357, 553) | 307  (240, 385) | 411  (332, 511) | 214  (176, 270) | 299  (248, 380) | 342  (265, 438) | 386  (312, 466) |
| MMA  (µmol/L) | 0.13  (0.11, 0.17) | 0.14  (0.11, 0.18) | 0.15  (0.12, 0.21) | 0.15  (0.12, 0.19) | 0.12  (0.10, 0.15) | 0.11  (0.10, 0.14) | 0.14 (0.11,0.20) | 0.12 (0.10,0.15) | 0.15  (0.12, 0.20) | 0.14  (0.12, 0.17) |
| Vitamin D3 (nmol/L) | 53.5  (40.3, 68.0) | 54.4  (39.6, 70.0) | 54.4  (40.5, 69.4) | 64.8  (54.5, 76.4) | 52.8  (41.5, 69.4) | 70.0  (57.6, 81.8) | 62.9  (41.0, 87.5) | 92.8 (73.0,106.9) | 62.3  (45.3, 77.6) | 65.1  (48.4, 78.2) |

HK ratio – 3´-hydroxykynurenine ratio, FMN – flavin mononucleotide, MMA - methylmalonic acid
