# Supplementary material for: Maternal B-vitamin and vitamin D status before, during, and after pregnancy and the influence of supplementation preconception and during pregnancy: Prespecified secondary analysis of the NiPPeR double-blind randomized controlled trial
Source: PLoS Med. 2023 Dec 5;20(12):e1004260. doi: 10.1371/journal.pmed.1004260 (PMC10697591; doi:10.1371/journal.pmed.1004260)
Supplement: S3 Table — (DOCX) [file pmed.1004260.s004.docx]

**Supplementary Table 3: Median (IQR) plasma concentrations in original units according to control or intervention group at each time-point for each site analysed separately**

| **Analyte (UK)** | Preconception baseline | | Preconception 1-month post supplementation | | Early pregnancy (7-11 weeks gestation) | | Late pregnancy (28 weeks gestation) | | 6 months post-delivery | |
| --- | --- | --- | --- | --- | --- | --- | --- | --- | --- | --- |
|  | Control  (n=197-229) | Intervention (n=209-231) | Control  (n=175-177) | Intervention (n=194-198) | Control  (n=97) | Intervention (n=107-110) | Control  (n=81-92) | Intervention (n=89-97) | Control  (n=60-71) | Intervention (n=69-79) |
|  | Median (IQR) | Median (IQR) | Median (IQR) | Median (IQR) | Median (IQR) | Median (IQR) | Median (IQR) | Median (IQR) | Median (IQR) | Median (IQR) |
| Folate  (nmol/L) | 17.6  (9.7, 33.4) | 18.3  (11.2, 30.0) | 35.6  (25.5, 49.5) | 39.1  (27.0, 49.7) | 48.4  (38.0, 56.8) | 51.4  (40.2, 61.7) | 34.7  (23.5, 44.4) | 37.0  (27.5, 50.6) | 16.1  (10.0, 25.4) | 16.1  (10.4, 27.5) |
| Homocysteine (µmol/L) | 7.7  (6.4, 9.1) | 7.5  (6.3, 9.2) | 7.5  (6.4, 8.7) | 7.0  (6.0, 8.0) | 5.9  (5.2, 6.7) | 5.2  (4.5, 5.7) | 4.6  (3.9, 5.3) | 3.8  (3.4, 4.5) | 8.2  (6.7, 10.1) | 7.5  (6.4, 8.8) |
| Riboflavin  (nmol/L) | 10.3  (6.5, 17.8) | 11.7  (7.2, 19.2) | 10.3  (6.6, 16.3) | 23.9  (15.1, 33.0) | 7.7  (5.6, 12.4) | 19.6  (13.0, 27.5) | 8.9  (5.3, 13.5) | 16.1  (11.5, 22.1) | 10.4  (8.0, 17.9) | 12.4  (8.9, 16.9) |
| FMN  (nmol/L) | 13.1  (10.3, 16.4) | 13.5  (10.9, 17.7) | 13.8  (10.6, 17.8) | 18.1  (13.8, 22.0) | 13.4  (9.9, 17.1) | 16.8  (14.2, 20.9) | 9.7  (8.0, 11.3) | 11.5  (9.5, 13.2) | 13.5  (11.4, 16.5) | 14.0  (11.6, 18.4) |
| Pyridoxal 5-phosphate (nmol/L) | 52.2  (38.6, 82.6) | 52.8  (39.8, 77.5) | 50.2  (40.5, 70.0) | 128.5  (93.9, 163.0) | 41.5  (32.1, 53.3) | 90.8  (68.0, 124.0) | 16.6  (12.8, 21.3) | 36.9  (26.1, 49.1) | 51.6  (38.0, 74.6) | 54.1  (39.1, 88.3) |
| HK ratio  (no units) | 0.4  (0.3, 0.4) | 0.4  (0.3, 0.4) | 0.4  (0.3, 0.5) | 0.3  (0.3, 0.4) | 0.4  (0.3, 0.4) | 0.3  (0.3, 0.4) | 0.5  (0.4, 0.6) | 0.5  (0.4, 0.6) | 0.4  (0.3, 0.5) | 0.4  (0.3, 0.5) |
| Cobalamin  (pmol/L) | 299.6  (249.8, 368.9) | 302.2  (242.7, 365.6) | 300.0  (249.4, 364.5) | 393.8  (320.6, 457.3) | 254.9  (202.6, 344.4) | 352.9  (298.8, 437.2) | 204.5  (153.8, 249.1) | 270.3  (220.6, 315.3) | 260.8  (208.5, 387.9) | 321.3  (270.1, 416.9) |
| MMA  (µmol/L) | 0.1  (0.1, 0.2) | 0.2  (0.1, 0.2) | 0.2  (0.1, 0.2) | 0.2  (0.1, 0.2) | 0.1  (0.1, 0.2) | 0.1  (0.1, 0.1) | 0.2  (0.1, 0.2) | 0.1  (0.1, 0.2) | 0.2  (0.1, 0.2) | 0.1  (0.1, 0.2) |
| Vitamin D3  (nmol/L) | 46.6  (34.2, 62.0) | 48.1  (34.4, 59.8) | 47.5  (34.3, 63.3) | 61.0  (52.1, 73.5) | 57.0  (42.5, 72.3) | 71.3  (59.2, 81.8) | 69.6  (45.5, 91.2) | 87.2  (70.1, 105.3) | 62.3  (37.6, 75.9) | 62.9  (42.7, 81.5) |

| **Analyte (SG)** | Preconception baseline | | Preconception 1-month post supplementation | | Early pregnancy (7-11 weeks gestation) | | Late pregnancy (28 weeks gestation) | | 6 months post-delivery | |
| --- | --- | --- | --- | --- | --- | --- | --- | --- | --- | --- |
|  | Control  (n=262-328) | Intervention (n=261-332) | Control  (n=276-279) | Intervention (n=289-292) | Control  (n=88-89) | Intervention (n=91-92) | Control  (n=69-82) | Intervention (n=71-84) | Control  (n=56-70) | Intervention (n=64-76) |
|  | Median (IQR) | Median (IQR) | Median (IQR) | Median (IQR) | Median (IQR) | Median (IQR) | Median (IQR) | Median (IQR) | Median (IQR) | Median (IQR) |
| Folate  (nmol/L) | 22.3  (12.1, 47.1) | 19.6  (11.3, 46.3) | 45.6  (32.3, 59.8) | 41.7  (29.8, 56.6) | 54.7  (42.5, 67.8) | 55.8  (45.5, 65.0) | 46.9  (30.5, 56.1) | 50.1  (36.2, 61.7) | 19.4  (12.0, 33.6) | 19.3  (13.4, 35.1) |
| Homocysteine (µmol/L) | 6.8  (5.9, 8.1) | 6.9  (6.0, 8.3) | 6.5  (5.8, 7.6) | 6.1  (5.4, 7.0) | 5.3  (4.7, 6.0) | 4.5  (4.1, 5.2) | 4.5  (3.9, 5.0) | 3.9  (3.5, 4.5) | 6.9  (5.6, 8.0) | 6.8  (5.7, 7.9) |
| Riboflavin  (nmol/L) | 13.7  (8.6, 24.5) | 11.8  (8.0, 20.8) | 13.7  (8.9, 25.3) | 25.8  (17.6, 36.8) | 12.9  (7.8, 25.3) | 18.8  (11.3, 29.6) | 11.6  (7.6, 16.7) | 16.9  (11.5, 22.2) | 14.8  (10.1, 21.1) | 11.8  (7.8, 23.1) |
| FMN  (nmol/L) | 14.2  (11.5, 18.0) | 14.4  (11.7, 18.1) | 16.0  (12.9, 20.9) | 18.4  (15.4, 23.1) | 16.3  (12.8, 20.5) | 17.8  (14.3, 22.8) | 11.1  (9.2, 12.6) | 11.2  (9.6, 13.8) | 14.3  (12.2, 17.1) | 14.2  (11.1, 18.6) |
| Pyridoxal 5-phosphate (nmol/L) | 52.3  (38.1, 83.3) | 52.5  (38.5, 78.8) | 57.0  (42.5, 76.5) | 144.5  (104.0, 198.5) | 54.6  (39.4, 70.6) | 117.5  (86.3, 166.5) | 27.7  (18.7, 40.7) | 53.8  (35.2, 71.1) | 61.3  (45.6, 98.1) | 63.8  (46.8, 92.6) |
| HK ratio  (no units) | 0.3  (0.3, 0.4) | 0.3  (0.3, 0.4) | 0.3  (0.3, 0.4) | 0.3  (0.2, 0.3) | 0.3  (0.3, 0.4) | 0.2  (0.2, 0.3) | 0.5  (0.3, 0.6) | 0.4  (0.3, 0.4) | 0.4  (0.3, 0.5) | 0.3  (0.3, 0.4) |
| Cobalamin  (pmol/L) | 379.4  (313.1, 459.8) | 373.1  (303.3, 462.5) | 389.4  (323.0, 480.8) | 487.5  (393.8, 606.3) | 353.9  (301.0, 429.9) | 493.2  (382.2, 584.5) | 234.7  (192.8, 279.3) | 336.0  (260.8, 412.2) | 402.3  (332.8, 494.8) | 459.9  (385.8, 555.9) |
| MMA  (µmol/L) | 0.1  (0.1, 0.2) | 0.1  (0.1, 0.2) | 0.1  (0.1, 0.2) | 0.1  (0.1, 0.2) | 0.1  (0.1, 0.1) | 0.1  (0.1, 0.1) | 0.1  (0.1, 0.2) | 0.1  (0.1, 0.1) | 0.1  (0.1, 0.2) | 0.1  (0.1, 0.2) |
| Vitamin D3  (nmol/L) | 44.1  (32.1, 55.3) | 42.0  (32.0, 54.2) | 45.9  (32.7, 56.9) | 56.7  (48.0, 67.1) | 47.9  (40.6, 57.6) | 60.6  (54.6, 75.5) | 55.8  (40.9, 75.7) | 87.0  (61.7, 103.4) | 54.8  (44.3, 65.2) | 61.3  (44.9, 68.7) |

| **Analyte (NZ)** | Preconception baseline | | Preconception 1-month post supplementation | | Early pregnancy (7-11 weeks gestation) | | Late pregnancy (28 weeks gestation) | | 6 months post-delivery | |
| --- | --- | --- | --- | --- | --- | --- | --- | --- | --- | --- |
|  | Control  (n=276-297) | Intervention (n=276-304) | Control  (n=241-251) | Intervention (n=241-257) | Control  (n=118-119) | Intervention (n=124-127) | Control  (n=111-114) | Intervention (n=110-112) | Control  (n=101-110) | Intervention (n=98-106) |
|  | Median (IQR) | Median (IQR) | Median (IQR) | Median (IQR) | Median (IQR) | Median (IQR) | Median (IQR) | Median (IQR) | Median (IQR) | Median (IQR) |
| Folate  (nmol/L) | 28.1  (15.4, 44.2) | 25.5  (14.0, 45.4) | 41.9  (30.2, 53.3) | 40.0  (29.9, 51.1) | 48.6  (41.1, 58.3) | 51.6  (42.9, 62.0) | 43.1  (37.1, 50.0) | 44.1  (37.5, 52.3) | 21.5  (12.7, 36.4) | 21.2  (13.5, 29.8) |
| Homocysteine (µmol/L) | 7.0  (6.1, 8.3) | 6.9  (6.0, 8.3) | 6.8  (6.0, 8.0) | 6.4  (5.5, 7.2) | 5.5  (4.7, 6.4) | 4.8  (4.1, 5.6) | 4.6  (4.1, 5.3) | 4.1  (3.6, 4.6) | 7.4  (6.5, 8.7) | 7.1  (6.2, 8.6) |
| Riboflavin  (nmol/L) | 13.4  (8.2, 22.0) | 12.1  (7.8, 22.1) | 14.1  (8.8, 22.7) | 24.1  (16.7, 38.4) | 11.7  (6.7, 18.8) | 21.4  (14.4, 36.3) | 10.4  (6.7, 15.1) | 18.1  (12.9, 24.1) | 13.4  (9.2, 22.9) | 14.1  (8.4, 24.0) |
| FMN  (nmol/L) | 16.3  (13.0, 20.0) | 15.6  (12.6, 20.1) | 16.5  (13.1, 21.3) | 18.6  (15.5, 23.9) | 14.0  (11.5, 17.9) | 17.5  (14.2, 22.2) | 10.1  (8.5, 12.0) | 11.6  (10.0, 13.7) | 14.7  (11.8, 18.1) | 15.3  (10.8, 18.8) |
| Pyridoxal 5-phosphate (nmol/L) | 62.0  (44.8, 113.0) | 65.2  (47.3, 99.8) | 60.7  (46.0, 90.1) | 146.0  (109.0, 186.0) | 46.9  (34.3, 63.4) | 108.0  (79.2, 139.0) | 17.9  (14.4, 24.0) | 40.5  (31.6, 54.4) | 53.4  (39.3, 91.3) | 54.0  (40.5, 78.6) |
| HK ratio  (no units) | 0.3  (0.3, 0.4) | 0.3  (0.3, 0.4) | 0.3  (0.3, 0.4) | 0.3  (0.2, 0.3) | 0.3  (0.3, 0.4) | 0.3  (0.2, 0.3) | 0.5  (0.4, 0.6) | 0.5  (0.4, 0.5) | 0.4  (0.3, 0.5) | 0.4  (0.3, 0.5) |
| Cobalamin  (pmol/L) | 363.1  (287.0, 448.5) | 362.9  (282.5, 442.4) | 369.9  (287.0, 454.1) | 448.6  (362.9, 548.7) | 299.7  (244.1, 374.2) | 418.1  (346.9, 509.3) | 214.9  (188.7, 270.1) | 316.9  (257.6, 395.7) | 335.4  (271.1, 417.6) | 379.0  (323.9, 460.0) |
| MMA  (µmol/L) | 0.1  (0.1, 0.2) | 0.1  (0.1, 0.2) | 0.2  (0.1, 0.2) | 0.2  (0.1, 0.2) | 0.1  (0.1, 0.2) | 0.1  (0.1, 0.1) | 0.1  (0.1, 0.2) | 0.1  (0.1, 0.1) | 0.2  (0.1, 0.2) | 0.1  (0.1, 0.2) |
| Vitamin D3  (nmol/L) | 61.6  (49.7, 77.1) | 63.3  (44.8, 78.2) | 61.1  (46.2, 77.3) | 67.2  (55.4, 80.2) | 59.7  (43.3, 74.9) | 70.8  (60.6, 82.5) | 62.9  (40.8, 89.4) | 96.9  (82.2, 112.0) | 69.1  (53.5, 84.8) | 70.8  (57.5, 86.1) |
