# Supplementary figures and images for: Maternal B-vitamin and vitamin D status before, during, and after pregnancy and the influence of supplementation preconception and during pregnancy: Prespecified secondary analysis of the NiPPeR double-blind randomized controlled trial
Source: PLoS Med. 2023 Dec 5;20(12):e1004260. doi: 10.1371/journal.pmed.1004260 (PMC10697591; doi:10.1371/journal.pmed.1004260)

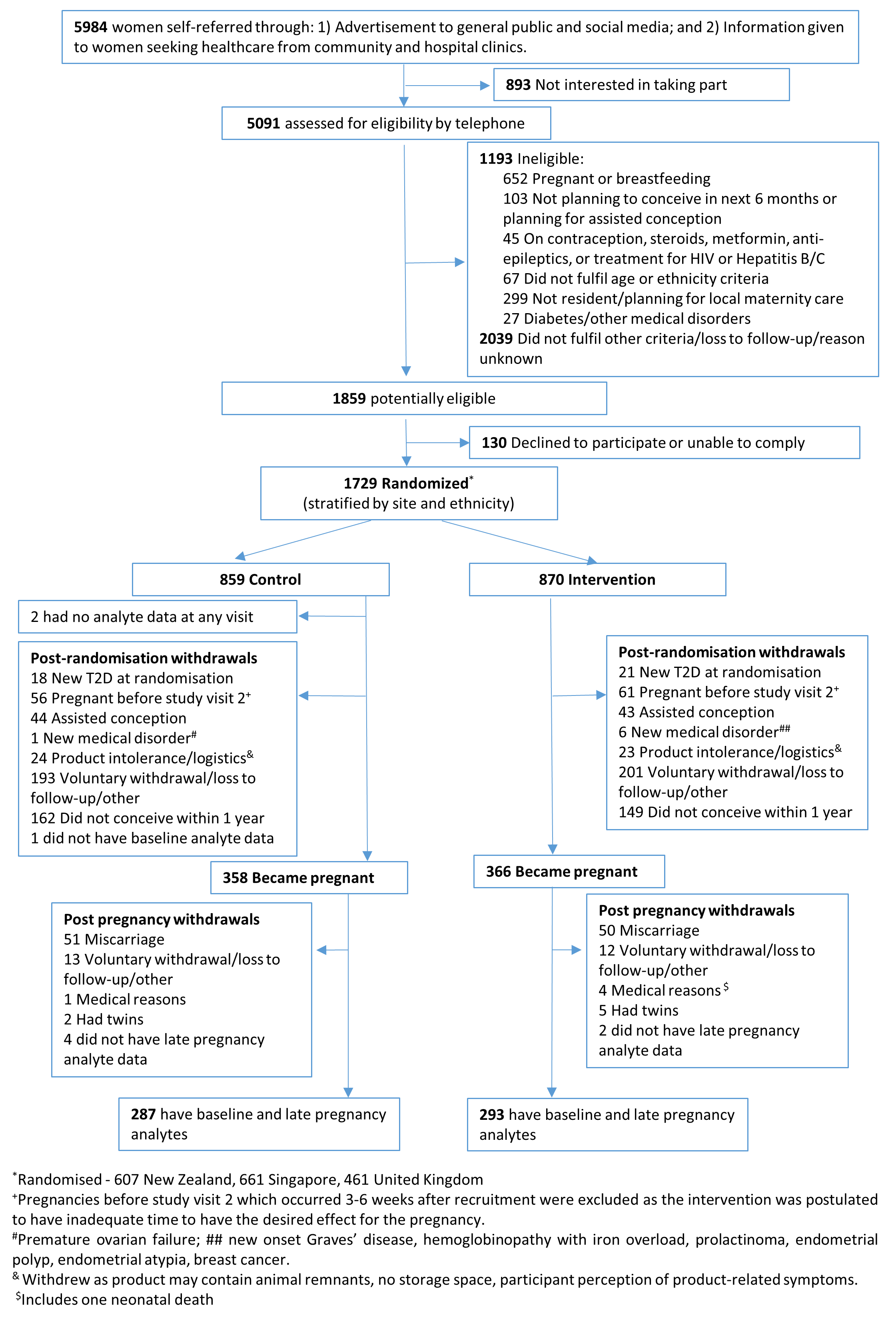

Supplement: S1 Fig — Abbreviation: Cesarean, cesarean section delivery. (TIF) [file pmed.1004260.s005.tif]

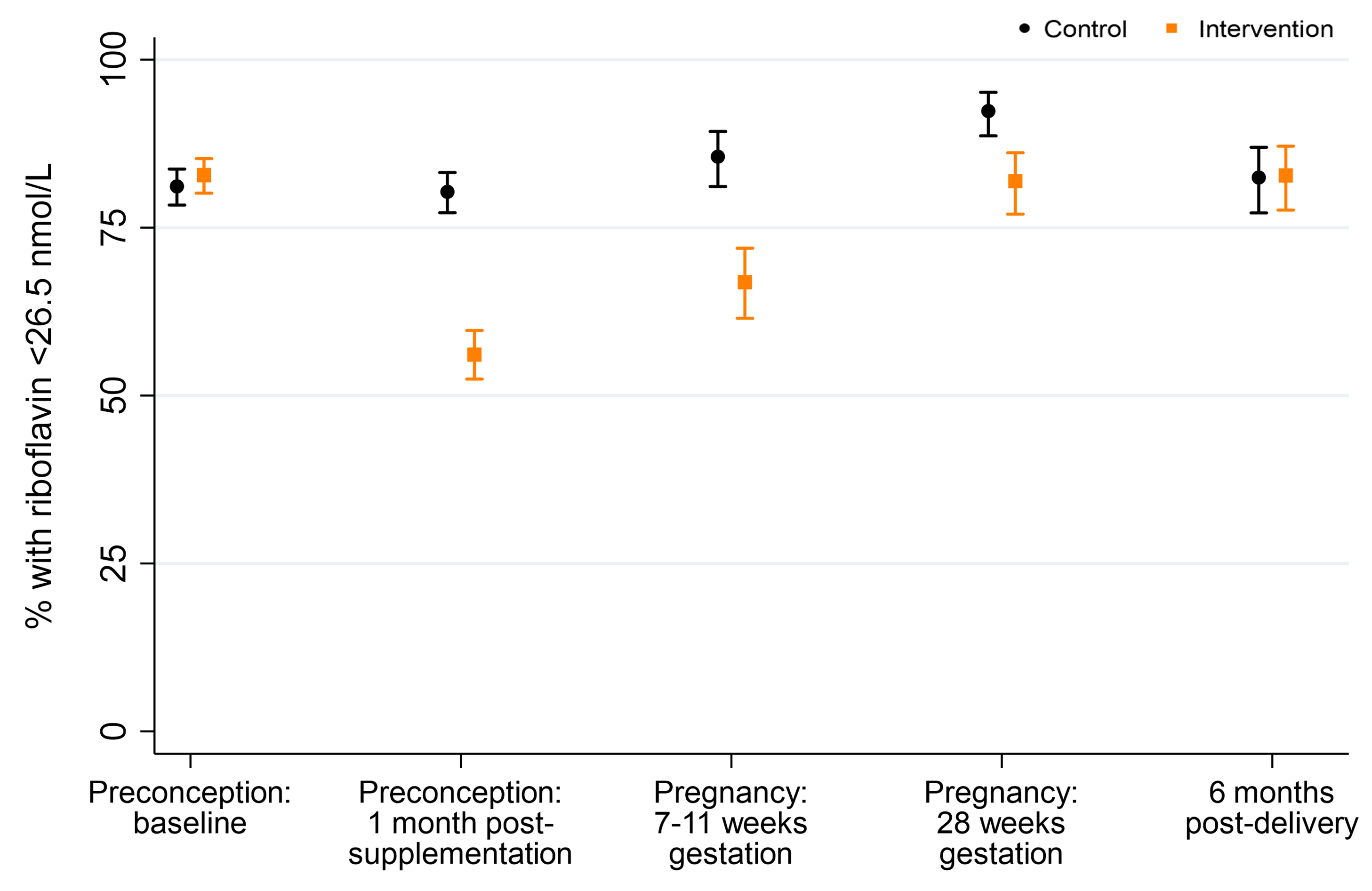

Supplement: S2 Fig — Footnote to S2 Fig: n = 854/867, 707/747, 305/329, 288/293, 251/261 for preconception baseline, preconception 1 month post-supplementation, early pregnancy, late pregnancy, and 6 months postdelivery, respectively. (TIF) [file pmed.1004260.s006.tif]

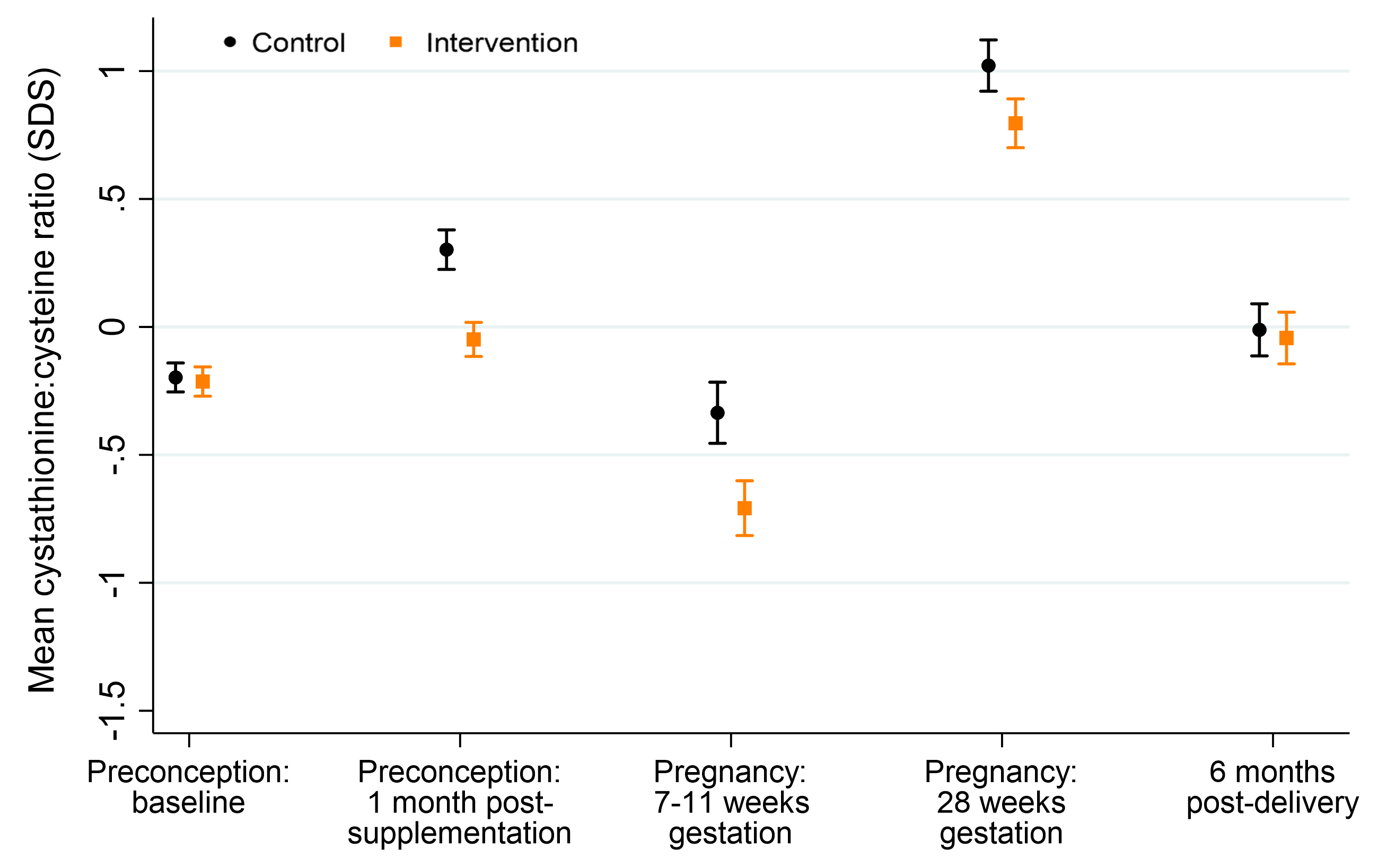

Supplement: S3 Fig — Footnote to S3 Fig: n = 851/864, 707/747, 305/329, 288/293, 251/261 for preconception baseline, preconception 1 month post-supplementation, early pregnancy, late pregnancy, and 6 months postdelivery, respectively. (TIF) [file pmed.1004260.s007.tif]

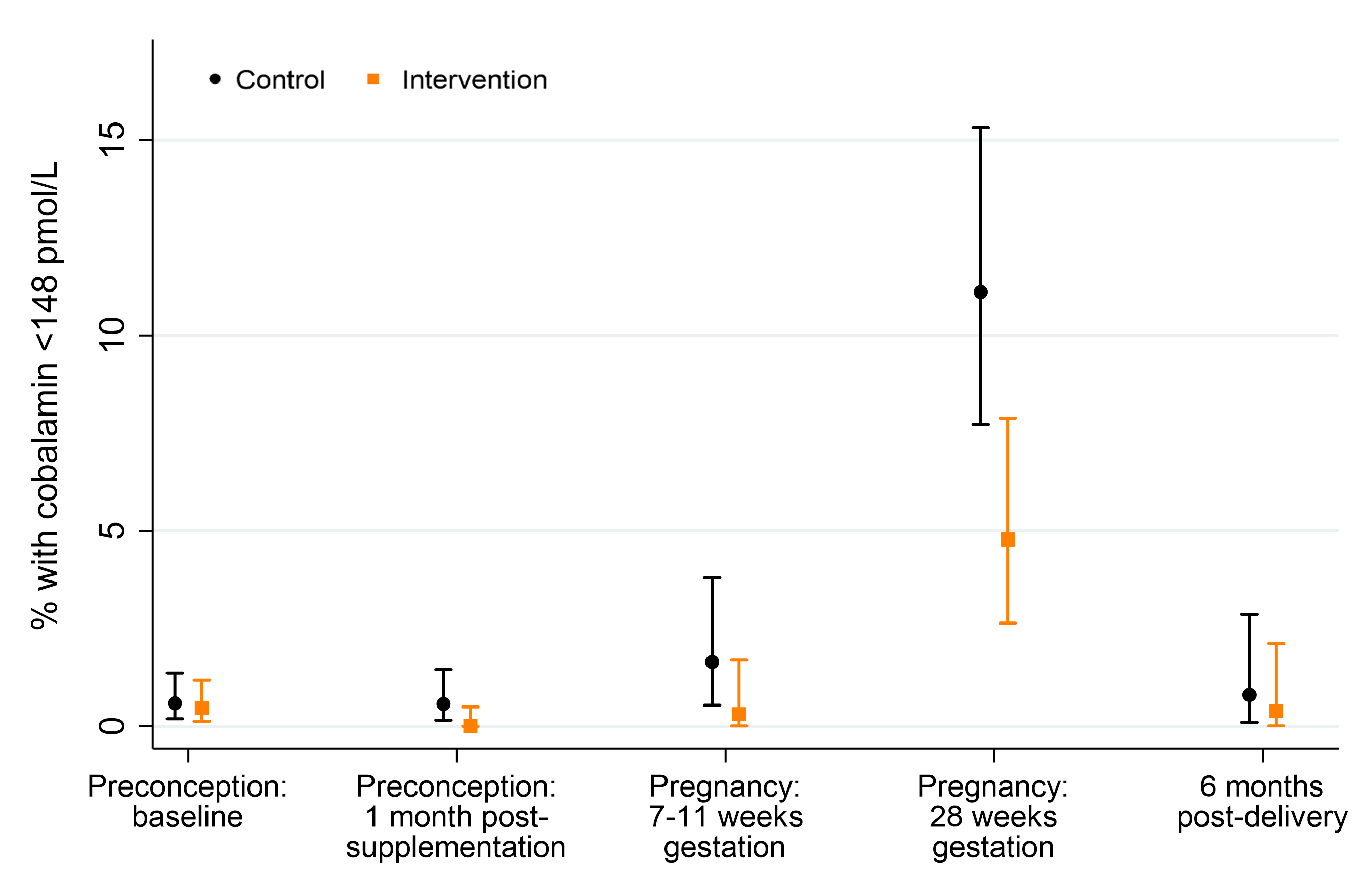

Supplement: S4 Fig — Footnote to S4 Fig: n = 853/864, 704/746, 304/327, 288/293, 250/261 for preconception baseline, preconception 1 month post-supplementation, early pregnancy, late pregnancy, and 6 months postdelivery, respectively. (TIF) [file pmed.1004260.s008.tif]
