# Supplementary material for: Maternal B-vitamin and vitamin D status before, during, and after pregnancy and the influence of supplementation preconception and during pregnancy: Prespecified secondary analysis of the NiPPeR double-blind randomized controlled trial
Source: PLoS Med. 2023 Dec 5;20(12):e1004260. doi: 10.1371/journal.pmed.1004260 (PMC10697591; doi:10.1371/journal.pmed.1004260)
Supplement: S1 Statistical Analyses Plan — (PDF) [file pmed.1004260.s009.pdf]

NiPPeR – Maternal Micronutrients

Statistical Analyses Plan

Table of Contents

1 INTRODUCTION 1

1.1 Foreword 1

1.2 Background and rationale 1

1.3 Effects of the intervention 2

1.3.1 Main objective 2

1.3.2 Additional objectives 2

2 METHODS 3

2.1 Study design 3

3 STATISTICAL METHODS 3

3.1 Longitudinal patterns and effects of the intervention on supplemented serum micronutrients 3

4 REFERENCES 5

TABLE 1. THRESHOLDS USED FOR DEFINITION OF MICRONUTRIENT DEFICIENCY 7

1 INTRODUCTION

1.1 Foreword

This statistical analysis plan provides an analysis plan for assessing longitudinal patterns and the impact of the NiPPeR intervention on serum micronutrients status and deficiency/insufficiency prior to pregnancy, during pregnancy, and postnatally.

1.2 Background and rationale

Micronutrients include a wide variety of non-energy providing dietary molecules with biological activity, including vitamins, coenzymes and minerals that are required in small quantities to support virtually all metabolic activity (1). Deficiency of vitamins cause recognisable disease, which in most cases resolves when the vitamin is replenished, so that prevention and treatment of deficiency is important. However, even outside of those bioactive molecules considered vitamins, a range of trace elements and other small molecules have been shown to have important health effects. In addition, metabolomic techniques are increasingly identifying small nutritionally related molecules that potentially influence health and metabolism.

Adequate levels of micronutrients are required to enable the fetus to develop and mature into a healthy neonate (1). A number of micronutrients have been shown to be beneficial as a supplement, to be taken through the pregnancy period; supplementation of folic acid is routine, and iron deficiency is screened for and treated when identified. In some countries such as New Zealand, supplementation with iodine is also routine.

The NiPPeR Study (Nutritional Intervention Preconception and During Pregnancy to Maintain Healthy Glucose Metabolism and Offspring Health) was a large clinical trial investigating nutritional effects on pregnancy and infant outcomes (2). Along with particular micronutrients, the intervention drink contained myo-inositol and probiotics, which were hypothesised to enhance alternate pathways of insulin action, resulting in healthy glucose tolerance during pregnancy (2). The intervention drink was enriched with certain micronutrients, namely vitamin D, riboflavin (B2), vitamin B6, vitamin B12, and zinc. Both the intervention drink and the control drink contained folic acid, iodine, calcium, beta-carotene and iron, so that they met the dietary supplementation typically provided as part of standard pregnancy care.

There is evidence of varying strength that the micronutrients common to both the control and treatment supplement have important effects on maternal or offspring health. Folic acid supplementation reduces the risk of neural tube defects (3), and may also reduce the risk of autism spectrum disorder, and improve cognitive and motor function in the offspring (4). Interestingly, a systematic review found that folic acid supplementation reduced the risk of having small-for-gestational-age (SGA) babies, but only if supplementation was started preconceptionally (5). This observation underscores the potential for greater benefit of supplements started in the preconceptional period. Iodine supplementation is also routinely recommended as it reduces the risk of cretinism and improves motor functions in regions where iodine deficiency is common (6). However, there is insufficient evidence exploring effects on neurodevelopment in children from regions with mild-moderate deficiency (6). Iron deficiency is routinely screened for during pregnancy; while supplementation of those found to have low iron stores appears to prevent iron deficiency anaemia in the mother, the effects on pregnancy and infant outcomes are inconsistent (7). Calcium supplementation at a high dose (>1 g/day) may reduce the risk of pre-eclampsia and preterm birth, particularly in those consuming low calcium diets (8). Calcium supplementation at lower doses (i.e. <1 g/day), more relevant to the NiPPeR treatments, may also reduce pre-eclampsia, hypertension, and NICU admission (8). However, vitamin A or carotenoid supplementation in low-income countries did not affect pregnancy outcomes, except in HIV-positive women where it protected against low birth weight (9).

The additional micronutrients present in the NiPPeR intervention (but absent from the control treatment) may also have important effects on maternal and offspring outcomes. Evidence for vitamin D

supplementation during pregnancy is generally of relatively low quality (10), but it may increase weight, length, and head circumference at birth and reduce the risk of low birth weight (10, 11), as well as reduce the risk of wheezing by 3 years of age (10). Evidence that riboflavin (vitamin B2) supplementation is beneficial is limited, but in anaemic pregnant women, it enhanced the effects of iron and folate supplementation (12), and supplementation of pregnant and lactating riboflavin-deficient mice enhanced weight gain, pup weight, and zinc absorption (13). Limited evidence has also suggested that pyridoxine (vitamin B6) may play a role in the prevention of pre-eclampsia, preterm birth, nausea in pregnancy, low apgar scores at birth, and the development of oro-facial and cardiovascular malformations (14). However, while a systematic review has shown that B6 supplementation in pregnancy may prevent dental decay in the offspring, there was insufficient evidence for other effects (14). An individual patient data meta-analysis showed that lower B12 levels were associated with preterm birth, and B12 deficiency was associated with lower birth weight (15), but a recent study on supplementation of pregnant women in India did not show any effect on neurophysiologic outcomes in 6-year-old offspring (16). B12 insufficiency has been associated with gestational diabetes in the setting of high folate status (17). Lastly, zinc deficiency has been associated with fetal loss, congenital malformations, intra-uterine growth retardation, reduced birth weight, prolonged labour, and both pre- and post-term deliveries (18, 19); however, currently there is weak evidence that zinc supplementation in pregnancy reduces the risk of preterm birth (20).

Replacing multiple micronutrients is appealing, as supplementation of many nutrients may be synergistic, and where multiple micronutrient deficiencies coexist, correction of just one may be insufficient. Potentially, multiple micronutrient replacement could improve enzymatic processes, signal transduction and transcription processes (21), and improve in placental function through modulation of inflammation, oxidative stress, and vascular function (22, 23). Multi-micronutrient supplements containing various combinations of vitamins A, B1, B2, B3, B6, B12, folic acid, C, D, E, and the minerals iron, selenium, copper, and zinc (24) have been shown in meta-analyses (predominantly in lower- and middle-income countries) to increase birth weight (25, 26), and lower the risk of low birth weight (25, 26) and SGA (27). The most recent Cochrane review of multiple micronutrient supplementation in pregnancy showed reduction in low birth weight, and probable reductions in SGA and preterm birth without any increase in mortality (23).

To have an important biological effect, micronutrients must be adequately absorbed and subsequently increase their respective concentrations in circulation or target tissues. Therefore, it is important that studies of micronutrient supplementation demonstrate such increases, in order to demonstrate a logical pathway for subsequent biological effects, or to help to interpret a lack of effect. In the NiPPeR study, the treatment and control supplements were consumed from the preconceptional period through to birth. Thus, it is important to determine whether the supplement had any effects on micronutrient levels throughout the preconceptional, pregnancy, and post-pregnancy periods.

During the NiPPeR study, micronutrient analysis was performed across these three important periods. Not only were vitamins and minerals relevant to the intervention treatment measured in maternal plasma/serum, but additionally, a range of vitamers, small metabolites, and markers of inflammation were measured. This presents a valuable opportunity to examine the associations between plasma micronutrient status and deficiency/insufficiency in the preconception, pregnancy, and post-pregnancy periods and a range of important domains assessed in NiPPeR.

1.3 Effects of the intervention

1.3.1 Main objective

To assess longitudinal patterns in concentrations and the potential effects of the NiPPeR treatment intervention on maternal plasma/serum concentrations status and deficiency/insufficiency of the micronutrients it included (i.e. vitamins D, B2, B6, and B12, as well as zinc) during the preconception, pregnancy, and post-delivery periods.

1.3.2 Additional objectives

To evaluate the potential effects of the NiPPeR treatment intervention on the circulating plasma concentrations of a range of other vitamers and small metabolites not included in the supplement, examining time points in the preconception, pregnancy, and post-delivery periods, focusing initially on those of most

relevance based on previous evidence, but then including an exploratory approach on other vitamers and metabolites.

2 METHODS

2.1 Study design

The NiPPeR study was a multi-centre, double-blinded randomised controlled trial that recruited women planning to conceive across three sites in the UK, Singapore, and New Zealand (2). The study's primary aim was to investigate the effect of a nutritional drink consumed twice daily during preconception and pregnancy on gestational glycemia. The control drink contained standard amounts of micronutrients that are part of routine pregnancy care (folic acid, β-carotene, iron, calcium, and iodine). In addition to containing these routine nutrients, the intervention drink was also enriched with vitamin D, zinc, riboflavin (B2), vitamin B6, and vitamin B12, myo-inositol, and probiotics (2). The study commenced in July 2015 and completed up to 12-month post-delivery visits to date, with ongoing 2- and 3-year post-delivery follow-ups.

Using a targeted method based on liquid chromatography-tandem mass spectrometry (Bevital, Bergen, Norway)(28), we measured plasma concentrations of vitamins present in the control and intervention groups, related vitamers and metabolites selected as those that reflect vitamin status: homocysteine (reflecting 1-carbon status and other physiological states, and an indicator of folate and B-vitamin deficiency), riboflavin, flavin mononucleotide (reflecting riboflavin status), pyridoxal 5-phosphate (vitamin B6), 3-hydroxykynurenine (HK), kynurenic acid (KA), anthranilic acid (AA), 3- xanthurenic acid (XA), hydroxyanthranilic acid (HAA), cystathionine, cysteine, methylmalonic acid and 25-hydroxyvitamin D3. Plasma folate and cobalamin (vitamin B12) were measured by microbiological assay, using a microtiter plate format on a robotic workstation employing a chloramphenicol-resistant strain of Lactobacillus casei (folate) and a colistin sulphate-resistant strain of Lactobacillus leichmannii (cobalamin)(Bevital, Bergen, Norway). Detailed quality control data for all analytes has been described previously, documenting coefficients of variation <10% (29). Values below the assay limit of detection were set to half the limit of detection value. Any hemolysis was visually graded from zero to 4+; anthranilic acid, 3-hydroxyanthranilic acid and 3-hydroxykynurenine values were set to missing for samples with a hemolysis score ≥ 2 (approximately equivalent to Hb 250 mg/dL), and those for folate set to missing for samples with 4+ hemolysis (approximately equivalent to Hb 1000 mg/dL). Each analyte was checked for outliers (both statistically and clinically), and implausible values were set to missing.

3 STATISTICAL METHODS

3.1 Longitudinal patterns and effects of the intervention on supplemented serum micronutrients

Hypothesis: NiPPeR's active treatment (containing vitamin D, B2, B6, B12, zinc, myo-inositol and probiotics) would affect circulating (serum/plasma) concentrations of these micronutrients

Aims: To evaluate longitudinal patterns and the potential effects of the NiPPeR intervention on the circulating concentration and deficiency/insufficiency of micronutrients that are provided in the NiPPeR intervention in the preconception, gestational, and post-delivery periods.

Outcomes: D vitamers – 25-OH D2, 25-OH D3
B12 (cobalamin), methylmalonic acid
B2 vitamers – riboflavin, flavin mononucleotide
B6 vitamers –pyridoxal 5'-phosphate, kynurenine metabolites, cystathionine/cysteine ratio
Folate (present in both treatment and control supplements), homocysteine
(note other micronutrients not measured at this stage)

Time points: Vitamers at baseline (pre-randomization; T1 in Figure 1), preconception (21–42 days after randomisation; T2), 7 weeks (T3) and 28 weeks (T5) of gestation, and 6 months post-delivery (T6).

Key predictors: NiPPeR intervention (treatment vs control)

Other key dependent co-variates: Study site, ethnicity; confirmatory analyses will also examine the effects of any co-variates not fully balanced across control and intervention groups.

Figure 1. Timing of serum samples collected for analysis of micronutrients.

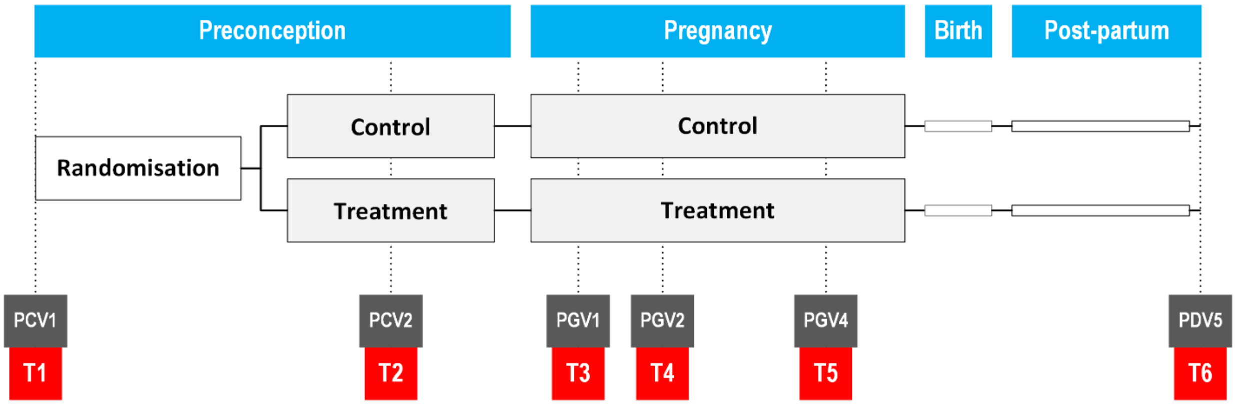

Data will be analysed on an intention-to-treat basis to address the main objective, i.e. to assess the effects of the NiPPeR intervention (i.e. treatment vs control) on plasma levels and deficiency/insufficiency of the supplemented micronutrients. Compliance with the study protocol was assessed by sachet counting, and for the primary trial analyses good compliance was defined *a priori* as ≥60% of the sachets taken. Notably, from the 585 participants recruited to NiPPeR who provided a primary outcome, only 20 appear to have had a reported compliance <60% (0.5%); thus we expect to exclude very few participants.

Potential differences in circulating micronutrient concentrations between the intervention groups will be assessed at each of the time points in cross-sectional analyses. The main analyses will examine each time point and nutrient individually. Thus, between-group comparisons will be assessed on a per-visit basis after both randomisation and treatment initiation: T2 (preconception), T3 (early pregnancy – first trimester), T5 (late pregnancy – third trimester), and T6 (6 months postnatally).

The distribution of micronutrient data will be examined, and, where appropriate, data will be log-transformed to approximate a normal distribution; in such cases, for reporting purposes model estimates will be back-transformed, and the estimated marginal medians with respective inter-quartile ranges will be shown. Analyses of the effect of the intervention on micronutrient deficiency/insufficiency will be undertaken using the cut points indicated in Table 1.

All tests will be two-tailed and carried out using Stata (StataCorp, College Station, TX, USA) or other statistical software as appropriate. There will be no imputation of missing data. Where a value was below the detection limit, its value will be set as half of the lower limit of detection. Statistical significance level will be set at $p<0.05$.

4 References

1. Gernand AD, Schulze KJ, Stewart CP, West KP, Christian P. Micronutrient deficiencies in pregnancy worldwide: health effects and prevention. Nature Reviews Endocrinology. 2016;12(5):274-89.
2. Godfrey KM, Cutfield W, Chan S-Y, Baker PN, Chong Y-S, Group NS. Nutritional intervention preconception and during pregnancy to maintain healthy glucose metabolism and offspring health (“NiPPeR”): study protocol for a randomised controlled trial. Trials. 2017;18(1):131.
3. Viswanathan M, Treiman KA, Kish-Doto J, Middleton JC, Coker-Schwimmer EJL, Nicholson WK. Folic Acid Supplementation for the Prevention of Neural Tube Defects: An Updated Evidence Report and Systematic Review for the US Preventive Services Task Force. JAMA. 2017;317(2):190-203.
4. Gao Y, Sheng C, Xie R-h, Sun W, Asztalos E, Moddemann D, et al. New perspective on impact of folic acid supplementation during pregnancy on neurodevelopment/autism in the offspring children–a systematic review. PLoS ONE. 2016;11(11):e0165626.
5. Hodgetts V, Morris R, Francis A, Gardosi J, Ismail K. Effectiveness of folic acid supplementation in pregnancy on reducing the risk of small-for-gestational age neonates: a population study, systematic review and meta-analysis. BJOG. 2015;122(4):478-90.
6. Zhou SJ, Anderson AJ, Gibson RA, Makrides M. Effect of iodine supplementation in pregnancy on child development and other clinical outcomes: a systematic review of randomized controlled trials. The American Journal of Clinical Nutrition. 2013;98(5):1241-54.
7. Cantor AG, Bougatsos C, Dana T, Blazina I, McDonagh M. Routine iron supplementation and screening for iron deficiency anemia in pregnancy: a systematic review for the US Preventive Services Task Force. Ann Intern Med. 2015;162(8):566-76.
8. Hofmeyr GJ, Lawrie TA, Atallah Á, Torloni MR. Calcium supplementation during pregnancy for preventing hypertensive disorders and related problems. Cochrane Database Syst Rev. 2018(10).
9. Thorne-Lyman AL, Fawzi WW. Vitamin A and Carotenoids During Pregnancy and Maternal, Neonatal and Infant Health Outcomes: a Systematic Review and Meta-Analysis. Paediatr Perinat Epidemiol. 2012;26(s1):36-54.
10. Roth DE, Leung M, Mesfin E, Qamar H, Watterworth J, Papp E. Vitamin D supplementation during pregnancy: state of the evidence from a systematic review of randomised trials. BMJ. 2017;359.
11. Maugeri A, Barchitta M, Blanco I, Agodi A. Effects of vitamin D supplementation during pregnancy on birth size: a systematic review and meta-analysis of randomized controlled trials. Nutrients. 2019;11(2):442.
12. Suprapto B, Widardo, Suhanantyo. Effect of low-dosage vitamin A and riboflavin on iron-folate supplementation in anaemic pregnant women. Asia Pac J Clin Nutr. 2002;11(4):263-7.
13. Agte VV, Paknikar KM, Chiplonkar SA. Effect of riboflavin supplementation on zinc and iron absorption and growth performance in mice. Biol Trace Elem Res. 1998;65(2):109.
14. Salam RA, Zuberi NF, Bhutta ZA. Pyridoxine (vitamin B6) supplementation during pregnancy or labour for maternal and neonatal outcomes. Cochrane Database Syst Rev. 2015(6).
15. Rogne T, Tielemans MJ, Chong MF-F, Yajnik CS, Krishnaveni GV, Poston L, et al. Maternal vitamin B12 in pregnancy and risk of preterm birth and low birth weight: A systematic review and individual participant data meta-analysis. Am J Epidemiol. 2017;185(3):212.
16. Srinivasan K, Thomas S, Anand S, Jayachandra M, Thomas T, Strand TA, et al. Vitamin B-12 Supplementation during Pregnancy and Early Lactation Does Not Affect Neurophysiologic Outcomes in Children Aged 6 Years. The Journal of Nutrition. 2020.

17. Kouroglou E, Anagnostis P, Daponte A, Bargiota A. Vitamin B12 insufficiency is associated with increased risk of gestational diabetes mellitus: a systematic review and meta-analysis. Springer; 2019.
18. Jameson S. Effects of zinc deficiency in human reproduction. Acta Med Scand. 1976(Suppl. 593).
19. Hambidge K, Neldner K, Walravens P. Zinc, acrodermatitis enteropathica, and congenital malformations. The Lancet. 1975;305(7906):577-8.
20. Chaffee BW, King JC. Effect of Zinc Supplementation on Pregnancy and Infant Outcomes: A Systematic Review. Paediatr Perinat Epidemiol. 2012;26(s1):118-37.
21. McArdle HJ, Ashworth CJ. Micronutrients in fetal growth and development. Br Med Bull. 1999;55(3):499-510.
22. Richard K, Holland O, Landers K, Vanderlelie JJ, Hofstee P, Cuffe JS, et al. Effects of maternal micronutrient supplementation on placental function. Placenta. 2017;54:38-44.
23. Keats EC, Haider BA, Tam E, Bhutta ZA. Multiple-micronutrient supplementation for women during pregnancy. Cochrane Database Syst Rev. 2019(3).
24. Pense J, Neher JO, Kelsberg G, Safranek S. Micronutrient Supplementation During Pregnancy. Am Fam Physician. 2015;92(3):222-3.
25. Shah PS, Ohlsson A. Effects of prenatal multimicronutrient supplementation on pregnancy outcomes: a meta-analysis. CMAJ. 2009;180(12):E99-E108.
26. Fall CH, Fisher DJ, Osmond C, Margetts BM. Multiple micronutrient supplementation during pregnancy in low-income countries: a meta-analysis of effects on birth size and length of gestation. Food Nutr Bull. 2009;30(4_suppl4):S533-S46.
27. Haider BA, Yakoob MY, Bhutta ZA. Effect of multiple micronutrient supplementation during pregnancy on maternal and birth outcomes. BMC Public Health. 2011;11(S3):S19
28. Midttun Ø, Hustad S, Ueland PM. Quantitative profiling of biomarkers related to B-vitamin status, tryptophan metabolism and inflammation in human plasma by liquid chromatography/tandem mass spectrometry. Rapid communications in mass spectrometry : RCM. 2009;23(9):1371-9.
29. Ulvik A, McCann A, Midttun Ø, Meyer K, Godfrey KM, Ueland PM. Quantifying Precision Loss in Targeted Metabolomics Based on Mass Spectrometry and Nonmatching Internal Standards. Analytical chemistry. 2021;93(21):7616-24..

TABLE 1. THRESHOLDS USED FOR DEFINITION OF MICRONUTRIENT DEFICIENCY

Micronutrient	Vitamin	Cut off	Description	Source
25-OH D3	D3	<50 nmol/L	deficient	Widely used in the published literature
25-OH D3	D3	<75 nmol/L	insufficient	Widely used in the published literature
Serum retinol	A	< 0.7 µmol/L	deficient	Bevital website
Riboflavin	B2	< 5 nmol/L	deficient	See explanatory note (1) below
Folate		< 6.8 nmol/L	deficient	See explanatory note (2) below
Folate		< 13.6 nmol/L	marginal	See explanatory note (2) below
Cobalamin	B12	< 100 pmol/L	severely deficient	See explanatory note (3) below
Cobalamin	B12	< 148 pmol/l	deficient	See explanatory note (3) below
Cobalamin	B12	< 221 pmol/L	depletion	See explanatory note (3) below
Pyridoxal 5'-phosphate	B6	< 20 nmol/L	deficient	See explanatory note (4) below

1. Riboflavin

Plasma/serum riboflavin reflects recent dietary intake and EFSA have indicated that there is no established cut-off value for plasma concentration to assess riboflavin deficiency and/or adequacy [1].

In the absence of such an established threshold, for initial analyses we will use a level of plasma riboflavin <5 nmol/L to indicate deficient riboflavin status as in previous SWS analyses.

1. https://efsa.onlinelibrary.wiley.com/doi/full/10.2903/j.efsa.2017.4919

2. Folate

In the literature there is some inconsistency in relation to definitions of folate deficiency or marginal status assessed using serum/plasma folate concentrations. We will define folate deficiency as plasma/serum folate concentrations **<3 ng/ml** (<6.8 nmol/L) [1] and marginal folate status as plasma/serum folate **<6 ng/ml** [2,3]. NB: some authors have used slightly different plasma folate cut-points for folate deficiency (<7 nmol/L [4,5]) and marginal folate status (7-14 nmol/L [6]).

For initial analyses we will use levels of plasma folate <6.8 nmol/L and <13.6 nmol/L to indicate and deficient and marginal folate status, respectively.

1. Quinlivan EP, Gregory JF. Effect of food fortification on folic acid intake in the United States. Am J Clin Nutr 2003;77:221–5.
2. Oner N, Vatansever U, Karasalihoğlu S, Ekuklu G, Celtik C, Biner B. The prevalence of folic acid deficiency among adolescent girls living in Edirne, Turkey. J Adolesc Health. 2006 May;38(5):599-606.
3. Reiter LA, Boylan LM, Driskell J, Moak S. Vitamin B-12 and folate intakes and plasma levels of black adolescent females. J Am Diet Assoc. 1987 Aug;87(8):1065-7.
4. Steenweg-de Graaff J, Roza SJ, Steegers EA, Hofman A, Verhulst FC, Jaddoe VW, Tiemeier H. Maternal folate status in early pregnancy and child emotional and behavioral problems: the Generation R Study. Am J Clin Nutr. 2012 Jun;95(6):1413-21
5. Simpson JL, Bailey LB, Pietrzik K, Shane B, Holzgreve W. Micronutrients and women of reproductive potential: required dietary intake and consequences of dietary deficiency or excess. Part I--Folate, Vitamin B12, Vitamin B6. J Matern Fetal Neonatal Med. 2010 Dec;23(12):1323-43.]
6. Chen KJ, Shaw NS, Pan WH, Lin BF. Evaluation of folate status by serum and erythrocyte folate levels and dietary folate intake in Taiwanese schoolchildren. Asia Pac J Clin Nutr. 2007;16 Suppl 2:572-8.

3. Vitamin B12

The recommended cut-offs for diagnosing vitamin B12 deficiency and depletion are <148pmol/l (**<200pg/ml**) and <221 pmol/l (**<300 pg/ml**), respectively, in plasma or serum [1,2]. In the Pune Maternal Nutrition Study 70% of mothers had B12 insufficiency during pregnancy (plasma concentration <150 pmol/l) and 31% were severely deficient (<100 pmol/l; <135.5 pg/ml)[3].

For initial analyses we will use a level of plasma/serum B12 <100 pmol/L to indicate severely deficient B12 status, <148 pmol/L to indicate deficient B12 status & <221 pmol/L to indicate depleted B12 status.

1. Simpson JL, Bailey LB, Pietrzik K, Shane B, Holzgreve W. Micronutrients and women of reproductive potential: required dietary intake and consequences of dietary deficiency or excess. Part I--Folate, Vitamin B12, Vitamin B6. J Matern Fetal Neonatal Med. 2010 Dec;23(12):1323-43.
2. Institute of Medicine and Committee (IOM). Dietary reference intake: folate, other B vitamins and choline. Washington, DC: National Academy Press; 1998.
3. Yajnik C et al. Vitamin B12 and folate concentrations during pregnancy and insulin resistance in the offspring. Diabetologia 2008;51:29-38.

4. Vitamin B6

Vitamin B6 status is usually assessed by plasma PLP levels. Exported from liver as a PLP-albumin complex, plasma PLP is considered a reflection of hepatic B6 levels and stores [1,2]. Plasma PLP level of <20 nmol/l is considered to reflect adverse vitamin status in the adult [3] although some support a threshold of 30 nmol/l [4] for assessing sufficiency. Plasma levels of other B6 vitamers are sometimes measured, but these tend to fluctuate more than PLP levels and are influenced by recent dietary intake [5]. PLP is very susceptible to degradation (either light-catalysed, or phosphatase-catalysed), so it is important to bear in mind potential storage or collection effects. In Sauberlich's book on 'Assessment of vitamin status', ed 2, 1999 p. 87: Low PLP is reported……during pregnancy (Reitman & Frankel, Am J Clin Path 28, 56 (1957); Karmen, Clin Invest 34, 131 (1955). Pyridoxal (PL) is simultaneously raised, however, so the sum of PLP + pyridoxal is constant (Van den Berg et al.,Int J Vit Nutr Res 48, 12 (1978); Barnard et al., J Nutr 117, 1303 (1987). Thus it would appear that phosphatase activity is probably raised during pregnancy, so the preparation and storage of the samples becomes even more critical than for non-pregnancy samples.

For initial analyses we will use a level of plasma/serum PLP <20 nmol/L to indicate deficient B6 status

1. Lumeng L, Ryan MP, Li TK. Validation of the diagnostic value of plasma pyridoxal 50-phosphate measurements in vitamin B6 nutrition of the rat. J Nutr 1978;108:545–553.
2. Li A, Lumeng L, Aronoff GR, Li T-K. Relationship between body store of vitamin B6 and plasma pyridoxal-P clearance: metabolic balance studies in humans. J Lab Clin Med 1985;106:491–497.
3. Coburn SP, Lewis DL, Fink WJ, Mahuren JD, Schaltenbrand WE, Costill DL. Human vitamin B6 pools estimated through muscle biopsies. Am J Clin Nutr 1988;48:291–294.
4. Leklem JE. Vitamin B-6: a status report. J Nutr 1990; 120:1503–1507.
5. Contractor SF, Shane B. Estimation of vitamin B6 compounds in human blood and urine. Clin Chim Acta 1968;21:71–77.
Institute of Medicine. Vitamin B6. Dietary reference intakes for thiamin, riboflavin, niacin, vitamin B6, folate, vitamin B12, pantothenic acid, biotin, and choline. Washington, DC: National Academy Press; 1998, pp. 150–195.
