# Supplementary material for: Maternal B-vitamin and vitamin D status before, during, and after pregnancy and the influence of supplementation preconception and during pregnancy: Prespecified secondary analysis of the NiPPeR double-blind randomized controlled trial
Source: PLoS Med. 2023 Dec 5;20(12):e1004260. doi: 10.1371/journal.pmed.1004260 (PMC10697591; doi:10.1371/journal.pmed.1004260)
Supplement: S1 Study protocol — (PDF) [file pmed.1004260.s010.pdf]

Nutritional Intervention Preconception and During Pregnancy to Maintain Healthy Glucose Metabolism and Offspring Health ("NiPPeR"): Study protocol for a randomised controlled trial

Keith M Godfrey[a,b*] kmg@mrc.soton.ac.uk
Wayne Cutfield[c,d] w.cutfield@auckland.ac.nz
Shiao-Yng Chan[e,f] obgchan@nus.edu.sg
Philip N Baker[c,g] philip.baker@leicester.ac.uk
Yap Seng Chong[e,f] yap_seng_chong@nuhs.edu.sg
NiPPeR Study Group

a NIHR Southampton Biomedical Research Centre, University Hospital Southampton, NHS Foundation Trust, Southampton, UK

b MRC Lifecourse Epidemiology Unit, University of Southampton, Southampton UK

c Liggins Institute, University of Auckland, Auckland, New Zealand

d A Better Start, New Zealand National Science Challenge, Auckland, New Zealand

e Department of Obstetrics and Gynaecology, Yong Loo Lin School of Medicine, National University of Singapore and National University Health System, Singapore

f Singapore Institute for Clinical Sciences, Agency for Science, Technology and Research, Singapore

g College of Medicine, Biological Sciences and Psychology, University of Leicester, Leicester, UK

***** Chief Investigator
Professor Keith Godfrey kmg@mrc.soton.ac.uk
MRC Lifecourse Epidemiology Unit
(University of Southampton)
Southampton General Hospital, Mailpoint 95
Southampton SO16 6YD United Kingdom

V1.11 7/10/2016

Abstract

Background

Improved maternal nutrition and glycaemic control before and during pregnancy is thought to benefit the health of the mother, with consequent benefits for infant body composition and later obesity risk. Maternal insulin resistance and glycaemia around conception and in early pregnancy may be key determinants of maternal physiology and placental function, affecting fetal nutrient supply and maternal-feto-placental communications throughout gestation, with implications for later postnatal health.

Methods

This double blind randomized controlled trial will recruit up to 1800 women, aged 18-38 years, who are planning a pregnancy in the United Kingdom (UK), Singapore and New Zealand, with a view to studying 600 pregnancies. The primary outcome is maternal glucose tolerance at 28 weeks' gestation following an oral glucose tolerance test. Secondary outcomes include metabolic, molecular and health-related outcomes in the mother and offspring, notably infant body composition. Participants will be randomly allocated to receive a twice-daily control nutritional drink, enriched with standard micronutrients, or a twice-daily intervention nutritional drink enriched with additional micronutrients, myo-inositol and probiotics both demonstrated previously to assist in maintaining healthy glucose metabolism during pregnancy. Myo-inositol is a nutrient that enhances cellular glucose uptake. The additional micronutrients seek to address deficiencies of some B-group vitamins and vitamin D that are both common during pregnancy and that have been associated with maternal dysglycaemia, epigenetic changes and greater offspring adiposity. Women who conceive within a year of starting the nutritional drinks will be followed through pregnancy and studied with their infants at six time points during the first year

of life. Blood, urine/stool, hair and cheek swabs will be collected from the mothers for genetic, epigenetic, hormone, nutrient and metabolite measurements, and assessments of the mother's body composition, anthropometry, health, diet and lifestyle will be made. Infants will also undergo hair, cheek swab, urine and stool sampling for similar biological measurements; infant body composition will be assessed and feeding recorded.

Discussion

There is an increasing focus on the need to optimise maternal nutrition starting prior to conception. This trial will provide evidence on the potential for nutritional interventions beginning prior to conception to promote healthy maternal and offspring outcomes.

Trial registration

This is an academic-led study by the EpiGen Global Research Consortium [ClinicalTrials.gov NCT02509988, Universal Trial Number U1111-1171-8056; 16/7/2015].

Keywords:

Preconception; Pregnancy; Randomised trial; Nutrition; Glucose metabolism; Metabolic diseases; Hyperglycemia; Body composition

Background

There is now considerable concern about the maintenance of healthy glucose metabolism during pregnancy. This has arisen by extrapolation from the increasing number of women who develop type 2 diabetes during their reproductive years [1,2]. Epidemiological studies show that children born to mothers with type 1 or 2 diabetes also have a greater susceptibility to diabetes and obesity in later life [3,4]. That this risk is related to intra-uterine exposure to hyperglycaemia is shown by the observation that, among siblings, the risk of diabetes is higher in those born after the mother was diagnosed with diabetes [5]. These observations have been extended recently, as offspring exposed even to mild hyperglycaemia during pregnancy have increased adiposity and are at increased risk of later diabetes and cardiometabolic disease [6,7]. Through transgenerational perpetuation of the cycle of 'diabetes begetting diabetes', these factors are driving further escalation of the epidemic of non-communicable diseases [8,9].

The rising levels of maternal adiposity and obesity are of particular concern in both developed populations and those undergoing rapid socio-economic transitions [1,10,11]. Maternal obesity is associated with increased risk of short-term adverse pregnancy outcomes as well as longer term impact on offspring health [12], which have been postulated to be partly mediated by greater maternal insulin resistance and higher glycaemia. Both with and without clinically-recognised pregnancy complications, evidence shows that a child of a mother with higher glycaemia *per se* may suffer from exposure to a suboptimal environment *in utero*, reducing the likelihood of a healthy body composition in the offspring and predisposing to increased childhood adiposity [13,14]. Feeding pregnant rodents a high fat diet gives rise to maternal obesity and hyperglycaemia, and offspring who become overweight demonstrate abnormalities similar to the

human metabolic syndrome; these are associated with epigenetic changes such as altered DNA methylation at specific genetic loci implicated in metabolic functions [15].

Pregnancy represents a state of relative maternal insulin resistance, which helps promote the transfer of nutrients such as glucose, fatty acids and amino acids to the fetus [16]. Placental nutrient transfer is determined by the concentration gradient, blood flow and the operation of active and facilitated transporters [17]. However, in contrast to amino acids, there is no upper limit to placental transfer of glucose and consequent fetal adipose accretion as maternal blood glucose levels rise [13]; this may be viewed as adaptive, as, in the neonatal period, relative adiposity provides metabolic reserves for thermogenesis and critical organs in the event of inadequate maternal care [18]. However, excessive materno-placental glucose transfer is associated with fetal hyperinsulinemia and macrosomia [19,20] and an increased risk of fatal obstructed labour, suggesting that the levels of glucose exposure of the fetus that are often now experienced are novel in evolutionary terms [21].

Gestational diabetes mellitus (GDM) can be envisaged as the more extreme outcome of physiological processes, when maternal insulin resistance is accentuated by the woman's own developmental, genetic and environmental circumstances: for example, women who themselves had a lower birth weight [22] or carry genetic variants associated with type 2 diabetes [23,24] are at increased risk of GDM. Established risk factors for developing GDM include pre-pregnancy obesity [25], excessive gestational weight gain [26], advanced maternal age [27] and a previous pregnancy with GDM [28]. These factors are now increasingly common in women during their reproductive years with the evolutionary mismatched situation of over-nutrition and low levels of physical activity contributing not only to the rise in GDM but to the increasing prevalence of

obesity and diabetes in their children, perpetuating a vicious cycle of disease. Such changes in growth potential and metabolic status may be mediated by inheritable epigenetic alterations occurring *in utero* [29]. For example, in Canadian first nation peoples, up to 30% of the incidence of type 2 diabetes has its origin in GDM in the previous generation [30]. Higher blood glucose levels in pregnancy carry risk of cardiovascular disease for both the mother as well as the child, a risk which increases with each pregnancy [31].

These findings have significant long-term implications for global public health. Now more than ever, effective strategies for maintaining healthy maternal glucose metabolism in pregnancy are needed. Such strategies would benefit both the mother in terms of a healthy pregnancy and her own metabolic health, and the offspring in terms of promoting healthy body composition and wellbeing.

There is now data indicating that deficiency or low levels of certain micronutrients (vitamins B6, B12 and D, riboflavin) is extremely prevalent in pregnant women and has lasting effects on the offspring's risk of obesity, acting through epigenetic processes [32-34]. Evidence from South Asian pregnant women supports a role for the combination of maternal vitamin B12 deficiency and folate sufficiency in promoting offspring adiposity, most likely mediated through impaired maternal glucose tolerance during pregnancy [35,36]. Meta-analysis of observational studies strongly points to a role for maternal vitamin D deficiency in GDM [37], and additional vitamin D in pregnant women with GDM has been shown to have beneficial effects on glycaemia and total and LDL-cholesterol concentrations [38]. Low zinc intake and status has also been linked with maternal glycaemia [39], and we propose that maternal glucose tolerance may be on the causal pathway linking maternal micronutrient deficiency to offspring adiposity. Importantly, among the pregnant women that we studied in Southampton and Singapore there was a low prevalence of deficiency

in folate and iron, the two most common micronutrients currently targeted for supplementation in pregnancy, and neither was associated with altered epigenetic adiposity biomarkers or with the child's adiposity.

Dietary myo-inositol is found in free form but can also be generated by microbial action in the gastrointestinal tract from food sources of phosphatidylinositol and phytic acid and its salts [40]. Myo-inositol is considered non-essential for mammals because it is synthesized de novo from glucose 6-phosphate in the kidney and other tissues [41,42]. Abnormalities in its metabolism have been associated with insulin-resistance and its depletion has been frequently observed in tissues affected by diabetic microvascular and neurological complications in animal models and human subjects [43]. Our current understanding of the molecular pathways of insulin action led to the hypothesis that the nutritionally derived myo-inositol may increase insulin sensitivity by making available more phosphatidylinositol and potentially inositol glycan secondary messengers [44,45]. An increasing number of publications suggest that myo-inositol may reduce insulin resistance during pregnancy [46-49].

Recent studies suggest that specific bacteria may positively influence cardiometabolic parameters, possibly through their interaction with the host and the effect of microbial-derived metabolites. There is now substantial evidence implicating a role for the gut microbiome in affecting glucose metabolism [50], and probiotics may modulate glucose tolerance through balancing gut microbiota, normalizing increased intestinal permeability and lowering systemic and local low-grade inflammation [51]. There is preliminary evidence that a combination of probiotic strains

during pregnancy may promote the maintenance of healthy glucose metabolism during pregnancy [52].

Taken together, there is strong support for new intervention studies commencing before pregnancy to provide myo-inositol and probiotics, and to improve maternal vitamin B6, vitamin B12, vitamin D, and zinc status, aimed at optimising maternal glycaemia and glucose supply to the feto-placental unit to promote healthy offspring growth and body composition.

Aim

This double blind randomized controlled trial in groups of women from different ethnic groups in the UK, Singapore and New Zealand is designed to examine the hypothesis that, compared with standard supplementation, a nutritional drink that contains myo-inositol, probiotics and additional micronutrients, commencing before conception and continuing during pregnancy, will assist in the maintenance of healthy glucose metabolism in the mother and promote offspring health.

Methods/Design

Trial design

Increasing evidence points to the preconception period and early pregnancy as a critical time when impaired maternal glucose tolerance may lead to biological alterations in the placenta and fetus that result in increased postnatal adiposity in the offspring [53]. As a consequence of this important evidence, our trial uniquely will focus on recruitment before conception and intervention both before and during pregnancy. Substantial experimental evidence from animal studies indicates that preconception is a critical period in the lifecourse for interventions to reduce later risk of metabolic dysregulation in the offspring. In humans, large cohort studies have demonstrated that preconception is a time when factors contributing to later ill-health begin to

operate, as poor maternal and paternal diet and smoking before conception impact on development and long-term health of the offspring; to date, however, there are no population-based trials of preconception nutrition in developed communities.

The flow of the trial is shown in the SPIRIT Figure. Extensive biosampling and detailed phenotyping are embedded in the study with longitudinal assessments at multiple time-points starting from the preconception phase throughout pregnancy and into the first year post-delivery. The biosampling and phenotyping will enable detailed mechanistic insights and characterisation of potential new interventions for investigation in future studies. Following informed consent at the first preconception visit, a baseline standard 75 g oral glucose tolerance test will be conducted, nutritional status, lifestyle, mood, body anthropometry and metabolic phenotype ascertained and biosampling undertaken, followed by randomisation to the intervention or control drink. At the second preconception visit a month later, further biosampling will be undertaken and body composition assessed by DXA (dual-energy X-ray absorptiometry) scanning. Regular in-person and phone contact will be made with participants to resupply control/intervention drinks, and to encourage retention and compliance during the preconception phase. Participants who become pregnant within a year of commencing the intervention or control drink will be seen around 7, 12, 20, 28 and 34 weeks of pregnancy for further phenotyping, biosampling and ultrasound scans assessing fetal growth and development. At 28 weeks gestation, a standard 75 g oral glucose tolerance test will be repeated to ascertain the primary outcome. Normal antenatal care will be permitted during the trial. The fathers will be interviewed to ascertain paternal lifestyle and mood, their anthropometry measured and paternal biosamples collected. At birth, offspring cord blood, umbilical cord and placental samples will be collected. Neonatal body composition is assessed by anthropometry, air displacement plethysmography (PEA POD) and, in a subsample, by DXA scanning. Both breast and formula-fed infants will be followed up when the infant is aged 1, 3 and

6 weeks, and 3, 6 and 12 months; infant feeding will be assessed in detail, biosamples collected, and growth and wellbeing ascertained. Breast milk samples will be collected from a subset of participants in early infancy for nutrient and metabolic analysis. A maternal oral glucose tolerance test will be repeated again at 6 months post-partum and repeat biosamples collected. The site visits will be completed at the research and hospital facilities of the three sites in Auckland (University of Auckland, Auckland, Waitemata and Counties Manukau District Health Boards and clinics, New Zealand), Singapore (National University Hospital and National University Health System Investigational Medicine Unit) and Southampton (National Institute for Health Research Wellcome Trust Southampton Clinical Research Facility and Princess Anne Hospital, University Hospital Southampton, UK).

Recruitment

Recruitment will be via self-referral of interested women who hear about the study via one or more of the following: a) local site advertisements in social (e.g. Facebook) and general (e.g. radio, local newspapers, magazines, posters) media, b) information brochures given to women engaging in community groups such as religious, culture-based or special-interest groups, c) information brochures given to women identified through or attending primary medical care, family planning or hospital clinics (for this group, eligible women may be contacted by a research nurse if they give permission to the clinic to pass on their contact details for this purpose).

Inclusion criteria are women who meet the following:

- Aged 18-38 years.
- Living in Southampton, Singapore or Auckland.
- In Southampton and Auckland, planning to have future maternity care in Southampton and Auckland respectively.

- In Singapore, willing to deliver at the National University Hospital.
- Women planning to conceive within 6 months (but conception up to 12 months after phenotyping will still be included).
- In Singapore only women of Chinese, Malay and Indian ethnicity, or of mixed Chinese/Malay/Indian ethnicity will be included.
- Able to provide written, informed consent.

Exclusion criteria are:

- Pregnant or lactating at recruitment (women who are currently breastfeeding will be excluded, but no washout period from the end of breastfeeding will be required before study start).
- Assisted fertility apart from those taking clomiphene or letrozole alone.
- Women with pre-existing type 1 or type 2diabetes (fasting plasma glucose concentration ≥ 7.0 mmol/l or post OGTT two hour plasma glucose concentration ≥ 11.1 mmol/l).
- Oral or implanted contraception currently or in the last month, or with an intra-uterine contraceptive device in situ.
- Metformin or systemic steroids currently or in the last month.
- Anticonvulsant medication currently or in the last month.
- Treatment for HIV, Hepatitis B or C currently or in the last month.
- Known serious food allergy.

Withdrawal criteria are:

- The participant wishes to discontinue participation in the study.
- The participant is unwilling or unable to comply with the protocol (including attendance at study visits, having study measures and biosampling).

- An overall uptake level of intervention/control nutritional drink of less than 60% evidenced by sachet counting.
- The participant is pregnant before or at preconception visit 2.
- The participant suffers a miscarriage (pregnancy loss before 24 weeks gestation) or ectopic pregnancy. If the participant suffers a first trimester pregnancy loss and wishes to re-join the study, she will be re-characterised as at the first baseline visit a month or more after a negative pregnancy test and will be assigned the nutritional drink with the same randomisation code as before.
- The participant presents with a multiple pregnancy (twins or other multiples).
- The infant dies in the perinatal period (for post-birth secondary outcomes).
- The participant suffers an adverse reaction which is deemed by the Investigator to be causally related to the intervention.
- The participant is withdrawn at the discretion of the Investigator for medical reasons.

For participants who withdraw during the pregnancy phase of the study, consent will be obtained to follow up on key outcome measures from their medical records to enable comparison of the characteristics of withdrawn and studied participants.

Randomisation procedure

At preconception clinic visit 1 all eligible participants will be randomised via the electronic study database to either the nutritional drink or the control drink. This database will assign each participant the appropriate code number that is consistent with either the intervention nutritional drink arm or the control drink arm. Randomisation will be stratified by site to ensure balanced allocation of participants across the two arms at each of the three sites, with further stratification

by ethnicity. Investigational products will be blinded by the manufacturer with non-speaking codes that do not allow deduction of the identity of intervention or control drinks. Investigators, staff performing the assessments, and data analysts will remain blind to the identity of the allocation from the time of randomisation until either the participant is unblinded or database lock of the primary outcome occurs. If emergency unblinding is necessary, the process for this will be documented in the study Safety Monitoring Plan.

The intervention

The Nutritional Intervention Preconception and During Pregnancy to Maintain Healthy Glucose Metabolism and Offspring Health (NiPPeR) intervention comprises: i) a micronutrient enriched nutritional drink containing myo-inositol, vitamin D, riboflavin, vitamin B6, vitamin B12 and zinc together with standard folic acid, iodine, calcium, β-carotene and iron; the quantities proposed are either standard amounts (myo-inositol [54]), enhanced amounts that are available in over the counter products (vitamins B6, B12, riboflavin), recommended daily allowance amounts in UK for pregnant women (vitamin D, zinc, folic acid, iodine) or minimal amounts for micronutrients linked with potential detrimental effects at higher doses (iron, β-carotene, calcium), and ii) probiotics (containing Lactobacillus rhamnosus NCC 4007 (CGMCC 1.3724) also known as LPR and Bifidobacterium animalis sp. lactis NCC 2818 (CNCM I-3446) also known as Bl818) [52]. The intervention group will be compared with a control group who receives a drink containing standard amounts of micronutrients that are part of routine pregnancy care (including folic acid, β-carotene, iron, calcium and iodine). The intervention is formulated as a powder in sachets to be made up in water immediately prior to consumption, with similar sensory characteristics for both the intervention and control drinks. The constituents of the intervention and control drinks are shown in Table 1, including the rationale for the amounts included.

This trial uses established nutritional elements for which tolerability is well established. Confirmation that the trial is not a Clinical Trial of an Investigational Medicinal Product has been secured from MedSafe (New Zealand), Medicines and Healthcare products Regulatory Agency, MHRA (UK) and the Health Sciences Authority (Singapore).

Outcome measurements

The primary analysis will adjust for site, ethnicity and preconception glycemia to account for potential imbalance between treatment arms amongst pregnancies which reach 28 weeks gestation, examining for differences in means between the control and intervention groups for the primary endpoint, specifically the fasting and/or 60 minute and/or 2 hour glucose concentrations following an oral 75g glucose tolerance test at 28 weeks gestation. Maternal glucose metabolism at 28 weeks' gestation has been chosen as the primary outcome as there is evidence that maintaining normal carbohydrate metabolism during pregnancy is associated with a healthier body composition, a reduced risk of obesity and potentially promotion of allergic/respiratory health in the children [55], alongside the recognised pregnancy benefits for the mother.

Secondary maternal outcomes of the initial phase of the NiPPeR study are:

1. Maintenance of a healthy pregnancy, including normal duration of gestation (≥37.0 weeks gestation), absence of GDM (defined using the International Association of the Diabetes and Pregnancy Study Groups criteria: glucose cut-off values of ≥5.1 mmol/L for fasting plasma glucose, and/or ≥10.0 mmol/L for 1-hour and/or ≥8.5 mmol/L for 2-hour post load), change in fasting glucose and OGTT glucose area under the curve from preconception baseline to 28 weeks gestation, maternal wellbeing/mood, absence of excessive nausea and vomiting, adequate pregnancy weight gain (Institute of Medicine criteria) and vaginal delivery rates.

2. Reduction in maternal micronutrient insufficiency, specifically less riboflavin, vitamin B6, vitamin B12, zinc and vitamin D insufficiency, before and during pregnancy

3. Alteration in gut microbiota consistent with enhanced wellbeing.

4. Alteration in maternal metabolomic and epigenetic biomarkers consistent with improved maternal and/or offspring wellbeing.

5. Enhancement of breast milk micronutrient content, altered immunological factors, epigenetic and metabolomic profiles (subsample), and maintenance of healthy lactogenesis.

Secondary offspring outcomes of the initial phase of the NiPPeR study are:

1. Neonatal adiposity measured by PEA POD.

2. Birthweight 2,500-4,000 g, size for gestational age at birth and customised birthweight centile.

3. Reduced adiposity gain during infancy, analysed taking account of infant feeding.

4. Reduction in cord blood C-peptide as a marker of overall glycaemia during gestation.

5. Promotion of offspring wellbeing and healthy cardiometabolic risk factors, including visceral adiposity and markers of insulin resistance, during infancy.

6. Alteration in offspring metabolomic and epigenetic biomarkers in perinatal samples, consistent with improved infant metabolic and allergic wellbeing.

7. Alteration in gut microbiota to a microbiota associated with infant metabolic and allergic wellbeing.

Data and biosample collection

Study data will be collected by trained research staff using an access controlled web based database (MedSciNet, Stockholm) managed with support from the data management staff of the MRC Lifecourse Epidemiology Unit and the Singapore Institute for Clinical Sciences. The study database will not hold personal information, which will be stored separately at each institution with access limited to study co-ordinators. Data from the web based database will be downloaded via a dedicated computer and stored securely on the MRC Lifecourse Epidemiology Unit's servers in the UK. Data extracts will be provided for analysis by the study researchers treating data from all three sites as a single study. All data will be kept in accordance with the UK Data Protection Act, and applicable regulations and guidance of each country and institution.

Biological samples will be collected and processed using standardised consumables, equipment and protocols across the three sites and will be being stored in accordance with the UK Human Tissue Act or equivalent at each institution in appropriately regulated biobanks. The study database allows management of the samples. All analysis will be carried out on anonymised data and samples. Accredited laboratories will be used for measurement of the primary outcome of plasma glucose concentrations.

Study management and governance

This double blind randomized controlled trial is led by investigators from the EpiGen Global Research Consortium, an academic research consortium comprising from the University of Southampton (MRC Lifecourse Epidemiology Unit and Institute of Developmental Sciences), University of Auckland (Liggins Institute), the Growth, Development, and Metabolism programme of the Singapore Institute for Clinical Sciences (an operating unit of A*STAR) and the National University of Singapore (Translational and Clinical Research Flagship Programme, `Developmental

pathways to metabolic disease'), together with the Singapore National University Health System. Scientists from the Nestlé Research Center (Nestec) provided advice on aspects of the intervention formulation.

The UK sponsor of the project is the University of Southampton; the New Zealand sponsor of the project is Auckland UniServices Limited; the Singapore sponsor of the project is the National University Hospital Singapore. The sponsors are indemnified for any harms arising from trial participation and will approve protocol amendments for which ethics approval has been secured, alongside update of trial registry entries. Trial oversight will be provided by an Independent Data Monitoring and Safety Committee. The day to day running of the study will be through the Trial Management Group, consisting of the Principal Investigators and Clinical Trial Operations Director, who will be responsible for all decisions on the study management and delivery.

Monitoring will be carried out several times per year by an external, independent monitor at each site, following the risk-based monitoring plan established for the study, overseen by the Study Sponsor. Safety reporting will be in accordance with the study Safety Monitoring Plan and all events will be recorded in the study database. An Independent Data Monitoring and Safety Committee has been established for the trial. This committee is independent from the sponsor and competing interests and will meet annually and oversee all ethical and safety issues in accordance with current regulations and MRC guidelines for Data Monitoring Committees. The Committee charter is available from the Clinical Trial Operations Director, who will coordinate and review activity across sites.

Statistical analysis

The primary analysis will be according to the intention to treat principle. Additionally, *a priori* sensitivity analyses will be undertaken, omitting participants withdrawn as a consequence of reluctance to continue with the trial, conception after taking the nutritional drink for <21 days, less than 60% uptake with the intervention (evidenced by sachet counting), not conceiving after 12 months of participation or not achieving a pregnancy >28 weeks. Further sensitivity analyses using a 'per protocol' or an 'as treated' analysis will be performed if deemed appropriate by the trial statisticians. For analysis of the primary endpoint only: if exploratory analysis reveals the presence of outliers, as identified by independent experts and/or the trial statisticians, sensitivity analysis will be performed excluding these outliers. Analyses will be specified in the study statistical analysis plan finalised by the Trial Management Group before unblinding the data. The influence of missing data will be examined using multiple imputation techniques. There are no formal planned interim analyses of the primary outcome, but progress reports on all data issues will be presented to the Independent Data Monitoring and Safety Committee, who will agree their charter at their first meeting. Analyses of the baseline phenotypic data that do not require unblinding will be undertaken. We will build prognostic models using baseline covariates on the primary outcome of maternal glucose tolerance in pregnancy. Planned subgroup analyses will include stratification by ethnicity across study sites.

Power calculations

By studying up to 900 pre-pregnant participants in each of the intervention and control groups, a total of 600 pregnancies and 500 live births is conservatively anticipated, following attrition from miscarriage, ectopic pregnancy, perinatal demise, multiple pregnancy, voluntary participant withdrawal, inability to comply with the protocol, withdrawal for medical reasons at the discretion of the investigator and loss-to-follow-up. A study of 250 pregnancies with 28 week OGTT data in

each group has 89% power to detect a 0.1 mmol/L reduction in fasting plasma glucose and 84% power to detect a 0.3 mmol/L reduction in 2 hour plasma glucose at the 5% level of significance. In the Hyperglycemia and Adverse Pregnancy Outcomes (HAPO) study such changes in glucose concentrations were associated with >10% changes in the odds of macrosomia and sum of neonatal skinfolds >90th centile and with a >20% change in cord C-peptide >90th centile [6]. As a further illustration of statistical power, using the distribution of 60 minute plasma glucose in the HAPO study, a study of 250 participants in each of the two groups has 80% power to detect a difference of ≥0.43 mmol/L in 60 minute glucose at $P<0.05$. A study of 300 pregnancies in each arm will have an 80% power to detect a reduction in the mean plasma glucose of 0.12, 0.45 and 0.34 mmol/L at fasting, 60 minutes and 2 hours respectively with an alpha of 0.017 taking into account multiple testing.

Discussion

This double blind randomized controlled trial in groups of women from different ethnic groups in the UK, Singapore and New Zealand is designed to examine the hypothesis that a nutritional drink, commencing before conception and continuing during pregnancy, will assist in the maintenance of healthy glucose metabolism in the mother and promote offspring health. Improved maternal nutrition and glycaemic control before and during pregnancy has benefits for the health of the mother and her offspring, including healthy offspring body composition, and decreased risks of childhood obesity and allergies. The intervention group will receive a nutritional drink enriched with micronutrients, myo-inositol and probiotics, and the control group will receive a drink enriched with standard micronutrients. The potential for adverse effects of the intervention is low as the probiotic and myo-inositol are thought to exert their main effects through physiological modulation of maternal metabolism rather than through direct effects on the fetus and, while the

amounts of micronutrients in the nutritional drink are sufficient to rectify maternal deficiency, they do not exceed UK, Singapore and New Zealand safe upper limits.

The trial commences preconception as studies by Catalano *et al* [53] suggest that a major part of the risk of macrosomia originates in early pregnancy/pre-pregnancy and adverse pregnancy outcomes are associated with poor maternal nutrition at conception; maternal insulin resistance and hyperglycaemia in the very earliest stages of pregnancy alter placental anatomy and physiology in ways that persistently affect transplacental fetal nutrient supply and fetal fat accretion, as well as bilateral maternal-feto-placental cross-talk, with consequences for later post-natal health. As a consequence, intervention commencing in established pregnancy can only partially influence fetal growth and development. The earlier maternal glycaemia is optimised and micronutrient deficiencies prevented, the greater the likelihood of maintaining fetal and postnatal health and wellbeing. Many influential governmental and non-governmental organizations are now stressing the importance of optimising preconception nutrition in general terms but as yet, other than folic acid to prevent neural tube defects, there are few preconception interventions that are recognized as promoting health benefits for the mother or offspring, and none, apart from folic acid, has a robust evidence base.

In the initial phase of the NiPPeR study a broad range of maternal nutritional assessments, potential epigenetic mechanisms and secondary measures relating to pregnancy outcomes and infant growth, body composition and wellbeing will be characterised and are detailed in this protocol.

The extensive biosampling and detailed phenotyping embedded in the study before, during and after pregnancy will provide an important discovery pipeline for the development of novel

biomarkers of maternal and offspring wellbeing, and lead to new interventions, and future guidelines to promote healthy human growth and development. A range of biological samples collected at multiple time points before, during and after pregnancy in the mother and offspring enables a systems biology approach to understanding the complex interaction of factors that determine maternal and infant wellbeing. Both individually and collectively, the control and intervention arms will provide extensive information that will deliver new knowledge on how maternal nutrition and metabolic state can promote offspring health. The research will also benefit from insights arising from other studies by the EpiGen Global Research Consortium in the UK, Singapore and New Zealand. The ethnicities of the participants in the study will allow broad extrapolation of the findings, and enable subsequent smaller scale studies in other jurisdictions such as China and India as appropriate. The partners have extensive experience of following up prospective mother-offspring cohorts, maternal, obstetrical, fetal and infant medicine and health care, and detailed characterisation of a comprehensive set of health and wellbeing outcomes through infancy and childhood will be undertaken. The data collected will allow determination of the contributions of nutritional and lifestyle factors, socioeconomic status, ethnicity, genetics, transcriptomics, epigenomics, metabolomics and metagenomics to maintaining healthy glucose metabolism in pregnancy and promoting healthy growth, body composition and wellbeing in the offspring.

Trial status

Recruitment for the trial commenced on 3/8/2015; more than half of the participants have been recruited within the following 12 months and recruitment is ongoing in October 2016. Participants have already progressed through the randomisation and pregnancy phases of the study and initial deliveries have occurred in all three sites.

List of abbreviations

GDM: Gestational diabetes mellitus, **HAPO:** Hyperglycemia and Adverse Pregnancy Outcomes, **NiPPeR Study**: Nutritional Intervention Preconception and During Pregnancy to Maintain Healthy Glucose Metabolism and Offspring Health Study, **UK:** United Kingdom

Declarations

Ethics approval and consent to participate

After independent full external peer review the study protocol and subsequent amendments have been approved by the research ethics committees at each of the three study sites (Southampton: Health Research Authority NRES Committee South Central Research Ethics Committee (REC), reference 15/SC/0142), the Health and Disability Ethics Committee (HDEC) (New Zealand) reference 15/NTA/21 and the National Healthcare Group Domain Specific Review Board (NHG DSRB) (Singapore) reference 2015/00205). The trial is an academic led study registered at ClinicalTrials.gov (NCT02509988), Universal Trial Number U1111-1171-8056. It has received intramural/infrastructure funding support at each of the three sites (UK Medical Research Council (MC_UU_12011/4); Singapore National Medical Research Council (NMRC/TCR/012-NUHS/2014); Gravida (National Centre for Growth and Development, New Zealand, no reference number)), with co-funding from Nestec Ltd (RDCU000485), who have formulated the trial intervention.

Information sheets are provided to potential participants ahead of them being approached for

consent by research staff. This study is being conducted in compliance with the protocol, Good Clinical Practice and the applicable regulatory requirements.

Consent for publication

Not applicable

Availability of data and material

A Trial Consultative Panel comprising senior representatives from the academic institutions undertaking the study and the industry partner has been set up and will consider associated studies requesting access to data and materials. This manuscript does not contain any data. Datasets during and/or analysed during the study will be available from the corresponding author on reasonable request. The results of the study will be submitted for publication in peer reviewed journals as soon as possible after analysis. Authorship will be in line with international guidelines (http://www.icmje.org/recommendations/browse/roles-and-responsibilities/defining-the-role-of-authors-and-contributors.html).

Competing interests

KMG has received reimbursement for speaking at conferences sponsored by companies selling nutritional products. KMG, WSC, CYS and SYC are part of an academic consortium that has received research funding from Abbott Nutrition, Nestec and Danone.

Funding statement

Public good funding for this investigator-led study is through the UK Medical Research Council (as part of an MRC award to the MRC Lifecourse Epidemiology Unit), the Singapore Government (as part of the Growth, Development and Metabolism Programme of the Singapore Institute for

Clinical Sciences) and the New Zealand Government (as part of the Gravida, Centre of Research Excellence: Growth and Development). Funding for provision of the intervention and control drinks and to cover aspects of the fieldwork for the study has been provided by Nestec SA under a Research Agreement with the University of Southampton, Auckland UniServices Ltd, Singapore Institute for Clinical Sciences, National University Hospital Singapore PTE Ltd., National University of Singapore.

Author’s roles

The concept for the study and its design was originated by KMG, CYS and PNB, with important inputs into the study design, protocol, standard operating procedures and delivery of the programme by SYC, WSC and the individuals in the NiPPeR Study Group. As Chief Investigator, KMG leads research planning and delivery across all three sites. KMG, WSC, SYC, PNB and CYS are Principal Investigators on the study Trial Management Group, which manages study delivery at the three sites, data analysis and interpretation and manuscript preparation. The manuscript was drafted by KMG, WSC, SYC, PNB and CYS. All authors have contributed to the draft protocol, approved the final manuscript and gave consent for its publication.

Acknowledgements

We would like to thank the participants for their enthusiastic involvement in the study.

References

1. Ferrara A. Increasing prevalence of gestational diabetes mellitus: a public health perspective. Diabetes Care 2007; 30 Suppl 2:S141-146.

2. Reece EA, Leguizamon G, Wiznitzer A. Gestational diabetes: the need for a common ground. Lancet 2009; 373:1789-1797.

3. Poston L. Developmental programming and diabetes - The human experience and insight from animal models. Best Pract Res Clin Endocrinol Metab 2010; 24:541-552.

4. Simeoni U, Barker DJ. Offspring of diabetic pregnancy: long-term outcomes. Semin Fetal Neonatal Med 2009; 14:119-124.

5. Dabelea D, Hanson RL, Lindsay RS, Pettitt DJ, Imperatore G, Gabir MM, Roumain J, Bennett PH, Knowler WC. Intrauterine exposure to diabetes conveys risks for type 2 diabetes and obesity: a study of discordant sibships. Diabetes 2000; 49:2208-2211.

6. Hyperglycemia and Adverse Pregnancy Outcome (HAPO) Study: associations with neonatal anthropometrics. Diabetes 2009; 58:453-459.

7. Tam WH, Ma RC, Yang X, Li AM, Ko GT, Kong AP, Lao TT, Chan MH, Lam CW, Chan JC. Glucose intolerance and cardiometabolic risk in adolescents exposed to maternal gestational diabetes: a 15-year follow-up study. Diabetes Care 2010; 33:1382-1384.

8. Ma RC, Chan JC. Pregnancy and diabetes scenario around the world: China. Int J Gynaecol Obstet 2009;104 Suppl 1:S42-5.

9. Yajnik CS. Fetal programming of diabetes: still so much to learn! Diabetes Care 2010; 33:1146-1148.

10. Ramachandran A, Ma RC, Snehalatha C: Diabetes in Asia. Lancet 2010; 375:408-418.

11. Chan JC, Malik V, Jia W, Kadowaki T, Yajnik CS, Yoon KH, Hu FB. Diabetes in Asia: epidemiology, risk factors, and pathophysiology. JAMA 2009; 301:2129-2140.

12. Godfrey KM, Reynolds RM, Prescott SL, Nyirenda M, Jaddoe VWV, Eriksson JG, Broekman BFP. The influence of maternal obesity on the long-term health of the offspring. The Lancet Diabetes & Endocrinology 2017;5:53-64.

13. Aris IM, Soh SE, Tint MT, Liang S, Chinnadurai A, Saw SM, Rajadurai VS, Kwek K, Meaney MJ, Godfrey KM, Gluckman PD, Yap FK, Chong YS, Lee YS. Effect of maternal glycemia on neonatal adiposity in a multiethnic Asian birth cohort. J Clin Endocrinol Metab. 2014;99:240-7.

14. Zhao P, Liu E, Qiao Y, Katzmarzyk PT, Chaput JP, Fogelholm M, Johnson WD, Kuriyan R, Kurpad A, Lambert EV, Maher C, Maia JA, Matsudo V, Olds T, Onywera V, Sarmiento OL, Standage M, Tremblay MS, Tudor-Locke C, Hu G; ISCOLE Research Group. Maternal gestational diabetes and childhood obesity at age 9-11: results of a multinational study. Diabetologia. 2016;59:2339-48.

15. Dudley KJ, Sloboda DM, Connor KL, Beltrand J, Vickers MH. Offspring of mothers fed a high fat diet display hepatic cell cycle inhibition and associated changes in gene expression and DNA methylation. PLoS One 2011; 6:e21662.

16. Lain KY, Catalano PM. Metabolic changes in pregnancy. Clin Obstet Gynecol 2007; 50:938-948.

17. Lewis RM, Demmelmair H, Gaillard R, Godfrey KM, Hauguel–de Mouzon S, Huppertz B, Larque E, Saffery R, Symonds ME, Desoye G. The placental exposome: placental determinants of fetal adiposity and postnatal body composition. Ann Nutr Metab 2013;63:208-215.

18. Kuzawa CW. Fetal origins of developmental plasticity: are fetal cues reliable predictors of future nutritional environments? Am J Hum Biol 2005; 17:5-21.

19. Susa JB, Schwartz R. Effects of hyperinsulinemia in the primate fetus. Diabetes 1985; 34 Suppl 2:36-41.

20. de Santis MS, Taricco E, Radaelli T, Spada E, Rigano S, Ferrazzi E, Milani S, Cetin I. Growth of fetal lean mass and fetal fat mass in gestational diabetes. Ultrasound Obstet Gynecol 2010; 36:328-337.

21. Godfrey KM, Haugen G, Kiserud T, Inskip HM, Cooper C, Harvey NCW, Crozier SR, Robinson SM, Davies L, the Southampton Women's Survey Study Group, Hanson MA. Fetal liver blood flow distribution: role in human developmental strategy to prioritize fat deposition versus brain development. PLoS One 2012;7:e41759.

22. Egeland GM, Skjaerven R, Irgens LM. Birth characteristics of women who develop gestational diabetes: population based study. BMJ 2000; 321:546-547.

23. Lauenborg J, Grarup N, Damm P, Borch-Johnsen K, Jorgensen T, Pedersen O, Hansen T. Common type 2 diabetes risk gene variants associate with gestational diabetes. J Clin Endocrinol Metab 2009;94:145-150.

24. Konig M, Shuldiner AR. The genetic interface between gestational diabetes and type 2 diabetes. J Matern Fetal Neonatal Med 2011, 25(1):36-40.

25. Graves E, Hill DJ, Evers S, et al. The impact of abnormal glucose tolerance and obesity on fetal growth. J Diabetes Res 2015; 2015:847674.

26. Gibson KS, Waters TP, Catalano PM. Maternal weight gain in women who develop gestational diabetes mellitus. Obstet Gynecol 2012; 119:560-565.

27. Solomon CG, Willett WC, Carey VJ, et al. A prospective study of pregravid determinants of gestational diabetes mellitus. JAMA 1997; 278:1078-1083.

28. Schwartz N, Nachum Z, Green MS. The prevalence of gestational diabetes mellitus recurrence - effect of ethnicity and parity: a meta-analysis. Am J Obstet Gynecol 2015; 213:310-317.

29. Godfrey KM, Costello PM, Lillycrop KA. The developmental environment, epigenetic biomarkers and long-term health. J Devel Origins Health Dis 2015;6: 399–406.

30. Dowse GK, Zimmet PZ, Finch CF, Collins VR: Decline in incidence of epidemic glucose intolerance in Nauruans: implications for the "thrifty genotype". Am J Epidemiol 1991, 133:1093-1104.

31. Sattar N, Greer IA: Pregnancy complications and maternal cardiovascular risk: opportunities for intervention and screening? BMJ 2002; 325:157-160.

32. Crozier SR, Harvey NC, Inskip HM, Godfrey KM, Cooper C, Robinson SM, SWS Study Group. Maternal vitamin D status in pregnancy is associated with adiposity in the offspring. Findings from the Southampton Women's Survey. Am J Clin Nutrition 2012;96:57–63.

33. Godfrey KM, Sheppard A, Gluckman PD, Lillycrop KA, Burdge GC, McLean C, Rodford J, Slater-Jefferies JL, Garratt E, Crozier SR, Emerald BS, Gale CR, Inskip HM, Cooper C, Hanson MA.

Epigenetic gene promoter methylation at birth is associated with child's later adiposity. Diabetes 2011;60:1528-34.

34. Childs C, Titcombe P, Crozier S, Barton S, Harvey N, Cooper C, Inskip H, Godfrey K. Low B-vitamin status during pregnancy is associated with greater offspring adiposity in childhood. J Devel Origins Health Dis 2015; 6 (suppl 2): S36.

35. Yajnik CS, Deshpande SS, Jackson AA, Refsum H, Rao S, Fisher DJ, Bhat DS, Naik SS, Coyaji KJ, Joglekar CV, Joshi N, Lubree HG, Deshpande VU, Rege SS, Fall CH. Vitamin B12 and folate concentrations during pregnancy and insulin resistance in the offspring: the Pune Maternal Nutrition Study. Diabetologia 2008;51:29-38.

36. Krishnaveni GV, Hill JC, Veena SR, Bhat DS, Wills AK, Karat CL, Yajnik CS, Fall CH. Low plasma vitamin B12 in pregnancy is associated with gestational 'diabesity' and later diabetes. Diabetologia 2009;52:2350-8.

37. Lu M, Xu Y, Lv L, Zhang M. Association between vitamin D status and the risk of gestational diabetes mellitus: a meta-analysis. Arch Gynecol Obstet 2016; 293:959-66.

38. Asemi Z, Hashemi T, Karamali M, Samimi M, Esmaillzadeh A. Effects of vitamin D supplementation on glucose metabolism, lipid concentrations, inflammation, and oxidative stress in gestational diabetes: a double-blind randomized controlled clinical trial. Am J Clin Nutr 2013 ;98:1425-32.

39. Bo S Lezo A, Menato G, Gallo ML, Bardelli C, Signorile A, Berutti C, Massobrio M, Pagano GF. Gestational hyperglycemia, zinc, selenium, and antioxidant vitamins. Nutrition 2005; 21: 186-91.

40. Schlemmer U, Frolich W, Prieto RM, et al. Phytate in foods and significance for humans: food sources, intake, processing, bioavailability, protective role and analysis. Mol Nutr Food Res 2009;53 Suppl 2; S330-375.

41. Clements RS, Diethelm AG. The metabolism of myo-inositol by the human kidney. J Lab Clin Med 1979;93: 210-219.

42. Hauser G, Finelli VN. The biosynthesis of free and phosphatide myo-inositol from glucose by mammalian tissue slices. J Biol Chem 1963;238: 3224-3228.

43. Croze ML, Soulage CO. Potential role and therapeutic interests of myo-inositol in metabolic diseases. Biochimie 2013;95: 1811-1827.

44. Coustan DR. Can a dietary supplement prevent gestational diabetes mellitus? Diabetes Care 2013;36:777-779.

45. Croze ML. Soulage CO. Potential role and therapeutic interests of myo-inositol in metabolic diseases. Biochimie 2013;95: 1811-1827.

46. D'Anna R, Scilipoti A, Giordano D, Caruso C, Cannata ML, Interdonato ML, Corrado F, Di Benedetto A. myo-Inositol supplementation and onset of gestational diabetes mellitus in pregnant women with a family history of type 2 diabetes: a prospective, randomized, placebo-controlled study. Diabetes Care 2013;36:854-7.

47. Corrado F, D'Anna R, Di Vieste G, Giordano D, Pintaudi B, Santamaria A, Di Benedetto A. The effect of myoinositol supplementation on insulin resistance in patients with gestational diabetes. Diabet Med 2011;28:972-5.

48. D'Anna R, Di Benedetto V, Rizzo P, Raffone E, Interdonato ML, Corrado F, Di Benedetto A. Myo-inositol may prevent gestational diabetes in PCOS women. Gynecol Endocrinol 2012;28:440-2.

49. Matarrelli B, Vitacolonna E, D'angelo M, Pavone G, Mattei PA, Liberati M, Celentano C. Effect of dietary myo-inositol supplementation in pregnancy on the incidence of maternal gestational diabetes mellitus and fetal outcomes: a randomized controlled trial. J Matern Fetal Neonatal Med 2013;26:967-72.

50. Cani PD, Geurts L, Matamoros S, Plovier H, Duparc T. Glucose metabolism: Focus on gut microbiota, the endocannabinoid system and beyond. Diabetes Metab. 2014; 40:246-57.

51. Isolauri E, Rautava S, Collado MC, Salminen S. Role of probiotics in reducing the risk of gestational diabetes. Diabetes Obesity Metab 2015;17:713-719.

52. Luoto R, Laitinen K, Nermes M, Isolauri E. Impact of maternal probiotic-supplemented dietary counselling on pregnancy outcome and prenatal and postnatal growth: a double-blind, placebo-controlled study. Br J Nutr 2010;103:1792-9.

53. Catalano P, deMouzon SH. Maternal obesity and metabolic risk to the offspring: why lifestyle interventions may have not achieved the desired outcomes. Int J Obes 2015;39:642-9.

54. Norwegian Scientific Committee for Food Safety. http://www.english.vkm.no/dav/dcbbd8d80c.pdf

55. Kumar R, Ouyang F, Story RE, Pongracic JA, Hong X, Wang G, Pearson C, Ortiz K, Bauchner H, Wang X. Gestational diabetes, atopic dermatitis, and allergen sensitization in early childhood. J Allergy Clin Immunol 2009;124:1031-8.

56. Lawlor DA, Wills AK, Fraser A, Sayers A, Fraser WD, Tobias JH. Association of maternal vitamin D status during pregnancy with bone-mineral content in offspring: a prospective cohort study. Lancet. 2013;381:2176-83.

57. http://www.cdc.gov/preconception/documents/clinical-content_womensnutritionfactsheet3.pdf

Table 1. Constituents of the intervention and control drinks

Intervention group	Daily amount	Rationale
Myo-inositol	4 g	Improves glucose metabolism and preliminary data suggest may maintain healthy glucose metabolism in pregnancy; dose safely used in pregnancy.
Vitamin D3	400 IU	Deficiency highly prevalent and linked with glucose metabolism in pregnancy and offspring postnatal adiposity gain; dose sufficient to reduce insufficiency while avoiding potential concerns re adverse effects at high doses. Omission from control group supported by a Lancet study [56].
Vitamin B6	2.6 mg	Deficiency highly prevalent and linked with glucose metabolism in pregnancy and offspring postnatal adiposity gain [33]; dose sufficient to rectify deficiency and present in current over the counter products (e.g. Elevit). Omission from control group supported by usual clinical practice.
Vitamin B12	5.2 µg	Deficiency highly prevalent and linked with glucose metabolism in pregnancy and offspring postnatal adiposity gain; dose sufficient to rectify deficiency and less than that in current over the counter products (e.g. Elevit). Omission from control group supported by usual clinical practice.
Riboflavin	1.8 mg	Low intake highly prevalent and linked with offspring postnatal adiposity gain [34]; dose sufficient to rectify deficiency and present in current over the counter products (e.g. Elevit). Omission from control group supported by usual clinical practice.
Zinc	10 mg	Deficiency highly prevalent and linked with offspring postnatal adiposity gain [unpublished]; dose sufficient to rectify deficiency and present in current over the counter products (e.g. Elevit). Omission from control group supported by usual clinical practice.
β-carotene	720 µg (15% of vitamin A requirements, as retinol equivalents)	Required in pregnancy in some jurisdictions.
Folic acid	400 µg	Standard pre-conception recommendation
Iron	12 mg	Iron is routinely prescribed and taken before/during pregnancy, though without convincing evidence of benefit; low dose included to lessen likelihood of additionally receiving a high dose iron product, which has been linked with glucose metabolism in pregnancy
Calcium	150 mg	A low dose of calcium is commonly taken before/during pregnancy; provision of this will lessen the likelihood of additional products being taken
Iodine	150 µg	Standard pre-conception recommendation
Probiotic		Taking a combination of two probiotics has been linked with maintenance of healthy glucose metabolism in pregnancy. Probiotic capsule containing > 1x10^9 cfu each of *Lactobacillus rhamnosus* NCC 4007 (CGMCC 1.3724) also known as LPR and *Bifidobacterium animalis sp. lactis* NCC 2818 (CNCM I-3446) also known as Bl818.
**Intervention and Control group**		
Folic acid	400 µg	Standard pre-conception recommendation
Iron	12 mg	Iron is routinely prescribed and taken before/during pregnancy, though without convincing evidence of benefit; low dose included to lessen likelihood of additionally receiving a high dose iron product, which has been linked with glucose metabolism in pregnancy.
Calcium	150 mg	A low dose of calcium is commonly taken before/during pregnancy; provision of this will lessen the likelihood of additional products being taken
Iodine	150 µg	Standard pre-conception recommendation [57]
β-carotene	720 µg (15% of vitamin A requirements, as retinol equivalents)	Required in pregnancy in some jurisdictions.

SPIRIT Figure: Trial Schema

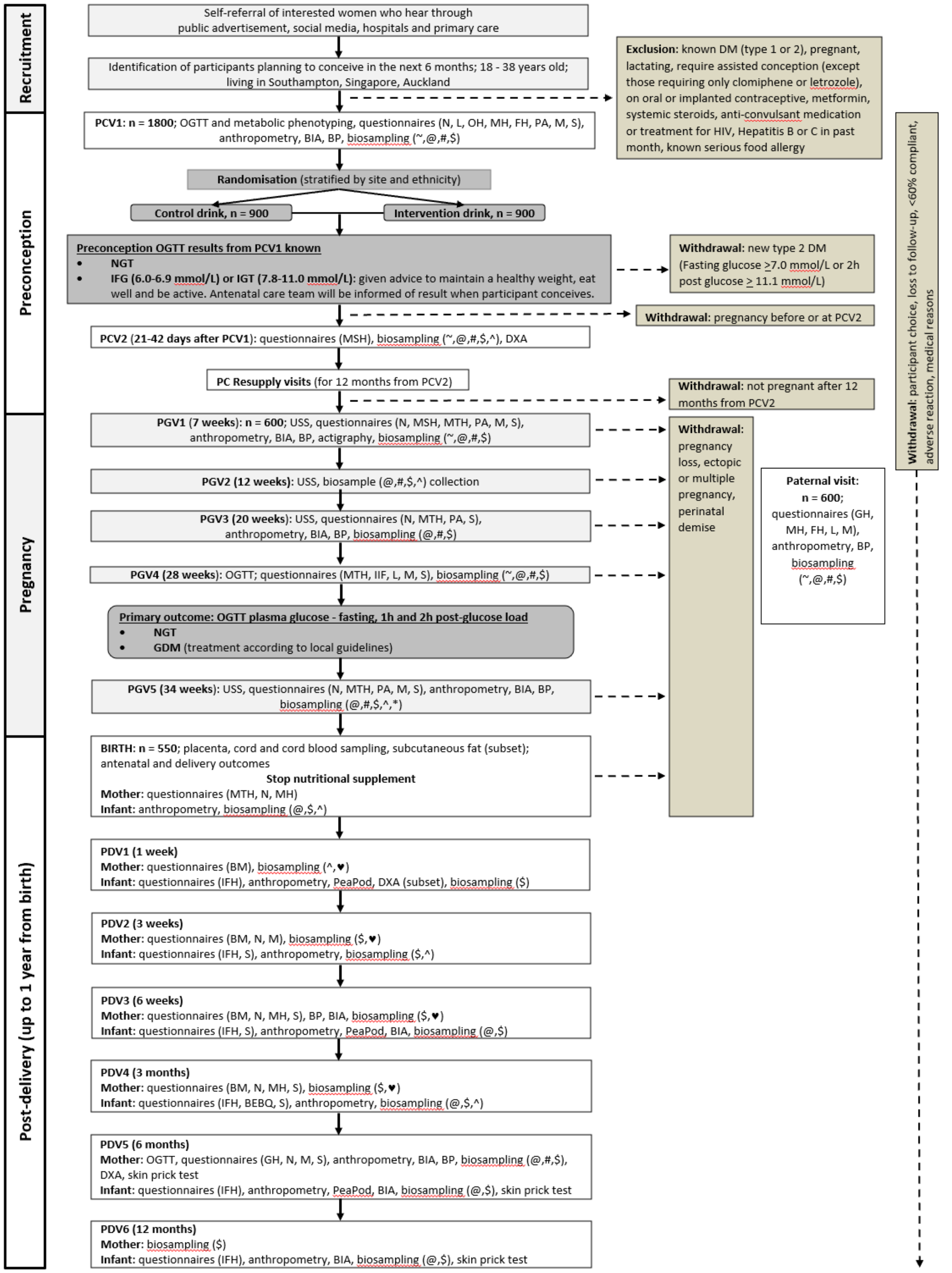

Abbreviations: PCV, Preconception visit; PC, Preconception; PGV, Pregnancy visit; PDV, Post-delivery visit; BIA, Bioelectrical impedance analysis; BP, blood pressure; DM, diabetes mellitus; DXA, dual-energy X-ray absorptiometry; GDM, gestational diabetes; HIV, human immunodeficiency virus; IFG, impaired fasting

glucose; IGT, impaired glucose tolerance; NGT, normal glucose tolerance; OGTT, oral glucose tolerance test; USS, ultrasound scan.

Questionnaires: BEBQ, baby eating behaviour; BM, breast milk; FH, family history; GH, general health; IFH, infant feeding and health; IIF, intentions for infant feeding; L, lifestyle; M, mood (Edinburgh Postnatal Depression Scale, State-Trait Anxiety Inventory); MH, medical history; MSH, menstrual history; MTH, maternal health; N, nutrition/diet; OH, obstetric history; PA, physical activity; S, sleep.

Biosampling: # = blood, ♥ = breast milk, $ = buccal swabs, * = epithelial swabs, @ = hair, ^ = stool, ~ = urine
